# Integrated analysis of root microbiomes of soybean and wheat from agricultural fields

**DOI:** 10.1038/srep28084

**Published:** 2016-06-17

**Authors:** Nicolás Rascovan, Belén Carbonetto, Diego Perrig, Marisa Díaz, Wilter Canciani, Matías Abalo, Julieta Alloati, Gustavo González-Anta, Martín P. Vazquez

**Affiliations:** 1 Instituto de Agrobiotecnología de Rosario (INDEAR), Ocampo 210 bis (2000), Predio CCT Rosario, Santa Fe, Argentina; 2 Rizobacter Argentina S. A, Argentina, Avda. Pte. Dr. Arturo Frondizi No. 1150-Calle No. 1, Parque Industrial, CP B2702HDA-Pergamino (Bs.As), Argentina

## Abstract

Root associated bacteria are critical for plant growth and health. Understanding the composition and role of root microbiota is crucial toward agricultural practices that are less dependent on chemical fertilization, which has known negative effects on the environment and human health. Here we analyzed the root-associated microbiomes of soybean and wheat under agricultural field conditions. We took samples from 11 different production fields across a large geographic area. We used 16S rRNA pyrosequencing to explore root microbial communities and also obtained 2,007 bacterial isolates from rhizospheres, which were tested for the presence of plant growth promoting (PGP) traits *in-vitro*. We observed that pH and nitrate content correlated with beta diversity variability of rhizospheric bacterial communities despite the variable field conditions. We described the dominant bacterial groups associated to roots from both crops at a large geographic scale and we found that a high proportion of them (60–70%) showed more than 97% similarity to bacteria from the isolated collection. Moreover, we observed that 55% of the screened isolates presented PGP activities *in vitro*. These results are a significant step forward in understanding crop-associated microbiomes and suggest that new directions can be taken to promote crop growth and health by modulating root microbiomes.

Agriculture is essential to supply the food demands of a growing human population. Since the “green revolution”, most worldwide crop production relies on the use of chemical fertilizers and pesticides^1^,^2^. However, agrochemicals have several negative effects to the environment and human health^3^,^4^. These chemicals mostly pollute the environment due to wasteful application and inefficient crop assimilation^5^. Therefore, current agricultural practices need to significantly change in order to reduce chemical use but retaining good harvest yields. In the last few decades biofertilizers based on specific strains of plant growth promoting (PGP) bacteria have been introduced as an alternative that reduces the use of chemicals and improves plant nutrient utilization^6^. Once applied, biofertilizers must interact with the rhizospheric soil (*i.e*., the soil closely attached to roots) microbiome and the root-associated microbes (*i.e*., endophytes and those lining in rhizoplane) to successfully colonize roots and to provide benefits to the plant^7^,^8^,^9^,^10^,^11^. In order to gain insights into this interaction we first need to know the complexity and diversity of native microbial communities associated to roots and the rhizosphere^11^,^12^,^13^.

The analysis of complex plant-associated microbiomes has been historically limited by technical constraints. By using high-throughput sequencing techniques we can now study plant-associated microbial communities with unprecedented detail. Such was the case of the recently revealed root-associated and rhizospheric soil microbiomes of *Arabidopsis thaliana* and *Hordeum vulgare* (domesticated barley)^14^,^15^,^16^,^17^. Other recent studies have focused on rhizospheric soil microbiota of soybean (*Glycine max*) in Amazon field environments and corn (*Zea mays)* in production fields in the United States^18^,^19^. Regardless of these foundational works, we know little about root microbiomes from crops and further research on root and rhizospheric soil microbiomes of commercial crops and non-model plants is currently highly required^12^,^20^,^21^. For instance, no research to-date has explored root-associated microbiomes of wheat (*Triticum aestivum*) and soybean in field conditions using high-throughput sequencing techniques, despite the fact that these are the fourth and ninth most produced crops in the world^22^. Estimated production for 2015/2016 is 733 million tons for wheat and 320 million tons for soybean^23^,^24^. Soybean is leguminous (order Fabales, family Leguminosae), a group of plants with the ability of developing complex symbiotic associations with nitrogen fixing rhizobacteria at root-nodules. Legumes are often considered to be the major nitrogen-fixing systems, as they may derive up to 90% of their nitrogen from N_2_^25^,^26^. On the other hand, wheat is a pasture that belongs to the order Poales, family Poaceae. Pastures are known to be important sinks for atmospheric CO_2_, and wheat, in particular, can transfer about 30% of assimilated CO_2_ into the soil through rhizodeposition^27^. Both crops are especially relevant since they can be efficiently alternated in crop rotations systems, where wheat is produced during winter and soybean during summer, providing complementary ecosystem services. Nevertheless, a deeper comprehension of the ecology and biology of their root microbiomes could significantly contribute in developing new agronomic/biotechnological tools to promote crop health and growth^11^,^12^,^28^.

Moreover, it has been proposed that bacterial strains are expected to perform better at root colonization and growth promotion if they are applied under similar conditions to those in their native environment^29^,^30^. Therefore, in order to improve agronomic tools based on the application of bacteria, we should first understand the variability of the root-associated microbiomes in different environments and identify the root-associated native species. Moreover the isolation and identification of native strains will be necessary to test the hypothesis of a better performance or these strains as PGP in native-like soil environments.

Here we present the first integrated comprehensive analyses of soybean and wheat root-associated and rhizospheric soil microbiomes from agricultural production fields distributed across an extensive geographic area. We analyzed crop microbiomes of 90 samples from 11 different locations using deep 16s rRNA gene amplicon pyrosequencing and massive bacterial isolation (2,007 isolates). In addition, we characterized plant growth promoting traits *in vitro* on the collection of isolates. By combining these strategies, we provide a novel perspective to the understanding of crop-associated microbiomes and propose that it might be possible to modulate the composition of root microbiomes to improve crop health and growth in the near future.

## Methods

### Site description and sampling

Samples were taken in experimental fields from 11 different locations in Argentine Pampas (Supplementary Fig. S1, Supplementary Table S1). Soils in Argentine Pampas are classified as Argiudolls and Hapludolls. Mean annual temperature ranges between 14 °C and 20 °C and mean annual rainfall ranges between 600 and 1,200 mm. Wheat (*Triticum aestivum*, cultivar Cadenza plants and associated rhizospheric soil were sampled in six locations between August and October 2012. Soybean (*Glycine max*, cultivar Wiliams 82) plants and associated rhizospheric soil were sampled in eight different locations (three were shared with wheat sampling sites) between January and February 2013. We sampled six replicated plots in each location. Each sampled replicate included 10 plants, which were carefully removed from the soil and sent immediately to the lab within the following hours. Bulk soil was also taken from plots to evaluate chemical and physical properties. Sampled experimental plots were subjected to continuous crop cultivation under no-tillage systems (i.e. minimal soil disturbance, permanent soil cover, rotations and fertilization). Nitrogen and phosphorus fertilizers were regularly used in sampled locations.

### Soil chemical and physical measurements

Soil organic carbon was determined by wet digestion and organic matter was estimated^31^. Nitrate-nitrogen was analyzed by 2 M KCl extraction and the phenol disulfonic acid method^32^. Extractable phosphorus was determined by the Bray method^33^. The pH was measured in a soil:water ratio of 1:2.5. Salinity was estimated by the determination of electrical conductivity^34^. Nitrogen was determined by Kjeldahl method^35^.

### Sample preparation

All samples were processed few hours after collection. Rhizospheric soil of five adjacent plants was combined as an individual sample. Roots were shaken to remove loose soil. Remaining attached soil (i.e. rhizospheric soil) was collected using sterile brushes. A total of 0.25 g of rhizospheric soil was used for DNA extraction with PowerSoil DNA Isolation Kit following the manufacturer’s instructions (MO BIO Laboratories, Inc.).

For Root-associated samples, roots from five plants were washed twice with sterilized water. Approximately 1 g of root tissue was pooled and washed in 15 ml 70% ethanol for 30 seconds. After washing with sterilized water, the roots were washed with 5% NaClO for 30 minutes and then washed with sterilized water. Roots were homogenized in liquid nitrogen with mortar and pestle and used for DNA extraction with PowerSoil DNA Isolation Kit (MO BIO Laboratories, Inc).

### Bacteria culture and isolation

The homogenized roots from root-associated sample preparation were diluted in 10 ml of sterilized water. 1 ml of this dilution was used for serial dilutions up to 10^−3^. A volume of 0.1 ml of root-derived dilution 10^−3^ was finally plated in petri dishes with nutrient broth agar. We used three different culturing approaches to isolate bacteria from the rhizospheric soil fraction. Roots with rhizospheric soil from five plants were shaken in 500 ml sterilized water during 1 hour. 1 ml of this dilution was used for serial dilutions up to 10^−3^. 1 ml aliquots of the last dilution were heated at 80 °C during 20 minutes and then plated in petri dishes with LB-Agar medium for isolation of sporulating bacteria^36^. 1 ml aliquots of the 10^−3^ dilution were plated in petri dishes with Cetrimide-Agar broth^37^ and NFB-agar broth^38^ to increase the odds of isolating of *Pseudomas spp*. and nitrogen fixing bacteria respectively. Petri dishes were incubated at 28 °C for 24 h. Colonies were picked and grown in 800 μl nutrient broth at 28 °C for an additional 24 h. Isolated strains were then stored at −80 °C in 20% glycerol. Isolates were screened for the presence of PGP-associated traits using previously described methods. Screeening included indole-acetic acid (IAA) production^39^, ACC deaminase activity^40^,^41^, nitrogen fixation^42^,^43^ and phosphorous solubilization using the Pikovskaya’s agar medium^44^. See supplementary text and Supplementary Fig. S2 for further information.

### 16S rRNA library preparation and sequencing

For pyrotag libraries the V4 hyper variable region of the 16s rRNA gene was amplified using the F515–R806 primers^45^. Samples were amplified using two rounds of PCR, following the procedures detailed in Rascovan *et al*.^46^. The first round was performed to amplify the V4 hyper variable region of the 16s rRNA (30 cycles) and a second round was performed to add barcodes (10 cycles). Duplicated reactions were performed in both rounds of PCR to reduce PCR biases. All amplicons were cleaned using Ampure DNA capture beads (Agencourt- Beckman Coulter, Inc.) and pooled in equimolar concentrations before sequencing on a Genome Sequencer FLX (454-Roche Applied Sciences) using Titanium Chemistry according to the manufacturer’s instructions.

### Pyrotags quality filtering and sequence analysis

Sequences were processed using the QIIME v1.9 analysis pipeline^47^. The SFF files were demultiplexed and quality filtered using the ampliconnoise.py script (which included PyroNoise and SeqNoises processing). This script also eliminated chimeras. A total of 254,345 quality-filtered reads were obtained with an average of 1,360 reads per sample (min = 95, max = 6,311). Samples with less than 400 reads were discarded. Phylotypes (OTUs) were clustered at 97% similarity using UCLUST with optimal parameters. Representative sequences were aligned against the Greengenes core set using PyNAST. The alignment was filtered to remove common gaps and a phylogenetic tree constructed de novo using FastTree. Rarefaction curves for richness were calculated by sub-sampling the OTU tables at different depths (200, 400, 600 and 800 sequences per sample) and counting the resulting number of phylotypes. Each sub-sampling was repeated 10 times. A total of 48 wheat samples (6 replicates per sampled site) and 42 soybean samples (3 replicates per sampled site) were used to build the plots. For Beta diversity analysis we used weighted UniFrac (WU) distance using 400 sequences per sample in order to include as many replicates as possible in the analysis^48^,^49^. Sequences were taxonomically classified using BLAST best hit against the NCBI 16S Microbial database. All scripts, commands and QIIME mapping and parameter files used for the bioinformatics analyses are listed at the end of Supplementary Information.

### Taxonomic classification of isolates

Taxonomic classification of isolates was done by colony PCR amplification of V4 hyper variable region of the 16S rRNA gene using 515F–806R primers^45^. Sanger sequencing of amplification products was performed by Macrogen Inc. (Seoul, Korea). BLASTN of the raw Sanger sequences against the OTU representative sequences calculated for the pyrotag dataset was done in order to trim the Sanger reads up to the pyrotag amplicon length (290 bp) (*i.e*. by only keeping the aligned region). Sequences with alignment lengths below 180bp were discarded. Filtered and trimmed sequences were taxonomically classified using BLASTN against the NCBI 16S Microbial database (Supplementary Table S2). Sequences with more than 90% similarity to best hit were classified up to the genus level.

### Pyrotag datasets vs. isolate collection

We used BLASTN to determine the similarities between the bacterial composition observed by pyrosequencing and in the isolates collection. We compared all RA pyrotag reads vs. Sanger 16 rRNA sequences obtained from rhizospheric isolates (both amplified with same primers) and quantified the proportion of reads with more than 97% similarity to best hit in the isolate collection. These sequences were considered as shared OTU-97% between both datasets. Sampling locations were not considered in the analysis

### Numerical analyses

Frequency distributions of pairwise weighted UniFrac distances were calculated 100 times for each data set (i.e. soybean RA, soybean RS, wheat RA and wheat RS microbiomes, see supplementary text for the scripts used in the analysis). All statistical calculations were carried out with R packages ‘stats’, ‘BiodiversityR’, ‘nparcomp’ and ‘Vegan’, unless stated. The Kolmogorov–Smirnov test was used to assess the difference between pairs of distributions. Kruskal-Wallis and Tukey non-parametric tests were used to compare OTU richness between sampled sites. Weighted UniFrac distances were visualized with principal coordinates analysis (PCoA). BIOENV analysis was performed to elucidate which soil properties (Supplementary Table S1) correlated with community patterns^50^. The correlations were evaluated for significance with Mantel test. We also used Mantel test to evaluate the correlation between weighted UniFrac distance matrices and Euclidean distance matrices of geographic locations of sampling sites (Supplementary Table S1). envfit() function in R was used to plot soil continue environmental variables in PCoA plots. Additionally, factorfit() R function was used to evaluate the correlation of community assemblies and soil type. Pearson correlation tests of taxa relative abundances with soil properties were performed with QIIME script otu_category_significance.py, and R scripts.

### Data Access

Amplicon sequencing raw datasets are available in the NCBI SRA database under the accession numbers: SRR3330055, SRR3330056 and SRR3330057 under the NCBI Bioproject PRJNA317286. Sanger sequences for the 16 rRNA gene obtained from isolates are available in the Supplementary Information section.

## Results

### Variation in OTU richness

**Figure 1:**
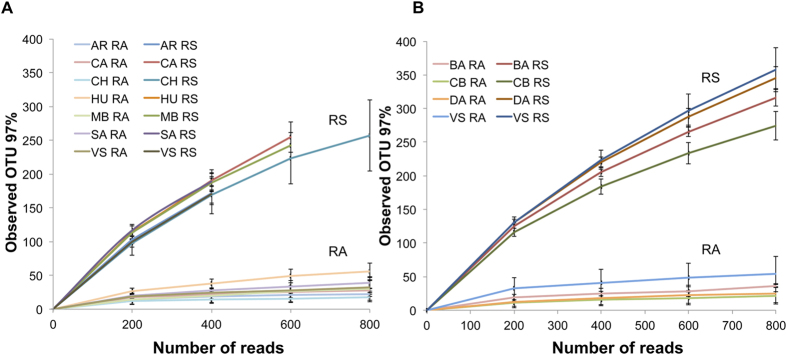
Alpha diversity from root microbiomes. Root-associated (RA) and rhizospheric soil (RS) microbiomes richness is expressed as number of observed operational taxonomic units (OTUs) at 97% similarity for soybean (**A**) and wheat (**B**). Error bars represent the standard deviation between replicates. AR, Aranguren; CA, Carmen de Areco; CH, Chilibroste; HU, Hughes; MB, monte Buey; SA, San Agustin; VS, Villa Saboya; BA, Balcarce; CB, Corral de Bustos; DA, Daireaux; SJ, San Jorge.

We collected samples of soybean and wheat from both the rhizospheric soil (RS) and the root-associated (RA) fractions across and extensive agricultural area (Supplementary Fig. S1 and Supplementary Table S1). As already described by Bulgarelli *et al*.^17^ we define RS fraction as the soil particles firmly attached to roots and the RA compartment as the root tissue depleted of soil particles by sequential washing treatments (which contains endophytes and may also have remaining bacteria from the rhizoplane). We characterized the bacterial communities in both fractions using 16S rRNA amplicon pyrosequencing. Bacterial alpha diversity, measured as OTU richness, was estimated for RA and RS microbiomes (Fig. 1). Rarefaction curves of RA microbiomes tended to an asymptote indicating that the sequencing depth was enough for an exhaustive exploration of the bacterial communities present in this fraction (Fig. 1). On the contrary, rarefaction curves from RS microbial communities did not saturate indicating that richness might be significantly higher than the observed (Fig. 1). Moreover, since results from RS samples were affected by an unexpected technical bias resulting in a low abundance of Proteobacteria sequences (see Supplementary text), richness in RS samples may be higher than shown here.

Microbial communities of soybean RS and wheat RS presented nearly ten times higher richness than RA microbial communities in all sampled sites. This difference may probably be higher if we take into account the bias mentioned above (Fig. 1). The differences in richness between RS and RA datasets were similar for all samples. This result could be explained by different carrying capacities at the rhizospheric soil and the root-associated microenvironments.

Estimated richness of wheat RS and soybean RS microbiomes differed between sampling sites (Kruskal-Wallis Test and Tukey non-parametric test p < 0.05, Supplementary Fig. S4A,C, and Supplementary Table S4). In addition, we observed differences in OTU richness in the soybean RA fraction between sampled sites but we did not find differences between sampled sites for the wheat RA fraction. (Kruskal-Wallis Test and Tukey non-parametric test p < 0.05, Supplementary Fig. S4B,D and Supplementary Table S4).

### Taxonomic characterization

The exploration of the taxonomic composition of the wheat RA and soybean RA microbiomes revealed that 98% of pyrotag reads obtained from soybean, and 79% from wheat, showed more than 97% similarity to the best Blast hit against the NCBI 16S database (Supplementary Fig. S3). This result indicates that the analyzed RA fraction is mainly dominated by bacteria closely related to previously characterized species.

**Figure 2:**
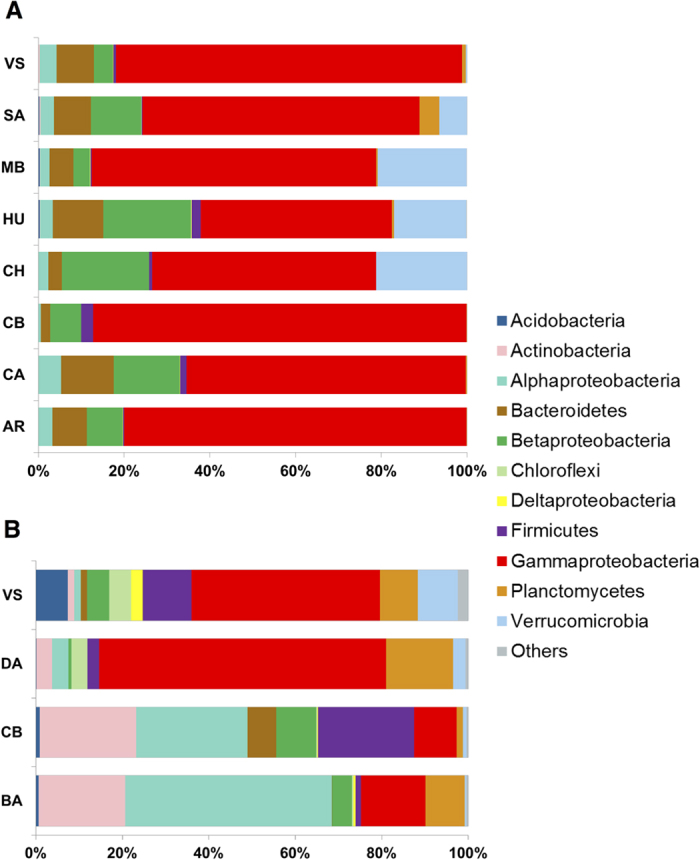
Bacterial taxonomic composition of the root-associated fraction of soybean and wheat microbiomes. Relative taxa abundance in soybean root-associated microbiomes (**A**) and wheat root-associated microbiomes (**B**). Sampled sites are represented along the vertical axis: AR: Aranguren,CA: Carmen de Areco,CH: Chilibroste,HU: Hughes,MB: Monte Buey, SA: San Agustin,VS: Villa Saboya, BA: Balcarce,CB: Corral de Bustos,DA: Daireaux, SJ: San Jorge.

We found that at the phylum level, soybean RA microbiomes showed less variation in the taxonomic composition between sites than wheat RA microbiomes (Fig. 2). Proteobacteria was the most abundant phylum in both crops. Most Proteobacteria reads found in the soybean RA dataset were classified as Gammaproteobacteria (Fig. 2A). This group was also the most abundant in wheat RA microbiomes of two of the sampled sites (Villa Saboya and Daireaux) while in the other two sites (Corral de Bustos and Balcarce) Alphaproteobacteria class was dominant (Fig. 2B). Planctomycetes, Firmicutes and Verrucomicrobia were also abundant in wheat and soybean RA microbiomes. Abundances of the mentioned taxa differed between sampled sites. Interestingly, a high abundance of poorly characterized bacteria (i.e., Plactomycetes and Verrucomicrobia) was observed in both crops.

The genus *Klebsiella* was the most abundant and ubiquitous in soybean RA microbiomes (45% ± 18% total reads in all samples, Supplementary Fig. S5). The genus *Pseudomonas* was also highly abundant and was found in all samples (Supplementary Fig. S5). *Stenotrophomonas* and *Rhizobium* were less abundant but ubiquitous while *Acinetobacter, Chryseobacterium*, *Acidovorax, Achromobacter*, *Agrobacterium* and *Burkholderia* were as well among the most abundant genera, although their presence and abundance were more variable across samples. Contrasting the results observed for soybean roots, no genus was consistently dominant in wheat RA microbiomes (Supplementary Fig. S6). The genera that showed the most ubiquitous presence were *Chthoniobacter*, (Verrucomicrobia phylum) and *Pseudomonas*, found in 96% and 86% of the samples respectively. In addition, *Blastocatella, Bacillus*, *Singulisphaera*, *Rhizobium* and *Klebsiella* were also among the most dominant genera (Supplementary Fig. S6).

### Variation in beta diversity

**Figure 3:**
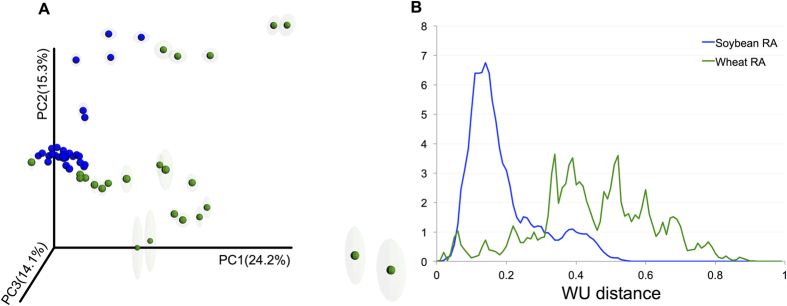
Beta diversity analysis in root microbiomes. (**A**) Principal coordinate analysis of based on OTU 97% and weighted unifrac distances of all root-associated (RA) from wheat (green) and soybean (blue). (**B**) Distribution of the weighted UniFrac (WU) pairwise distances between all samples from RA datasets based on OTU-97% communities.

We observed significant differences between wheat and soybean RA communities based on weighted UniFrac distances in a PCoA analysis (Fig. 3A, ANOSIM R = 0.4, p = 0.001). We further explored the distribution of pairwise weighted UniFrac distances and found that wheat RA and soybean RA communities showed significantly different profiles (Fig. 3B, Kolmogorov–Smirnov test, D = 0.733, p-value < 0.0001). While the distances between soybean samples were among the lowest values in the distribution, the distances between wheat sample pairs were among the highest. These results together with taxonomic composition results, suggest that the soybean RA microbiome is less variable than the wheat counterpart.

**Figure 4:**
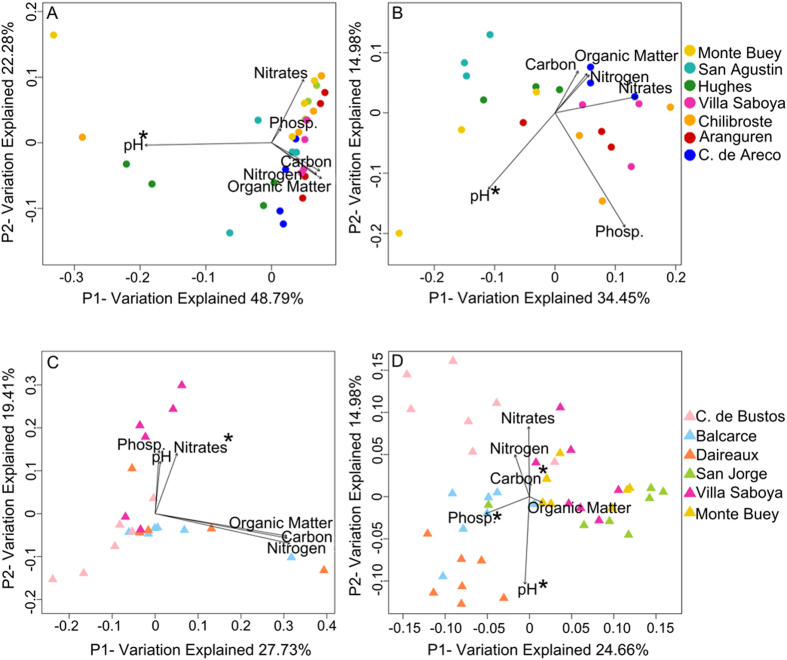
Correlation between soil properties and root community strucutre. Principal Coordinate Analysis visualization based on weighted UniFrac distances of soybean root-associated microbiomes (**A**), soybean rhizospheric soil microbiomes (**B**), wheat root-associated microbiomes (**C**) and wheat rhizospheric soil microbiomes (**D**). Vectors show fitted values of soil properties. Asterisk (*) indicates soil properties that correlate with the microbial community structure (BIOENV and Mantel Test, p ≤ 0.05).

It was previously shown that sampling location and geochemical and physical variables may affect RS bacterial communities of soybean and wheat^18^,^51^. Moreover, it was proposed that these environmental properties also affect RA bacterial communities in *Arabidopsis thaliana* and barley^14^,^17^. However, the latter has not yet been evaluated for RA microbiomes of soybean and wheat. In order to asses if any of the measured soil properties could potentially be associated with the phylogenetic variation between microbiomes, we used Clarke and Ainsworth’s BIOENV analysis^50^. The results showed that pH was the variable that best correlated with soybean RA community assemblies (Fig. 4A,B, Mantel Test p ≤ 0.05). Moreover, we analyzed the correlation of pH with taxa relative abundances and found a positive correlation with class Bacilli within the RA fraction (Supplementary Fig. S7, Pearson correlation r = 0.60, p ≤ 0.05). When we analyzed correlations at the genus level, we found that relative abundances of *Paenibacillus* (Pearson correlation r = 0.60, p ≤ 0.05), *Bradyrhizobium* (Pearson correlation r = 0.61, p ≤ 0.05) and *Variovorax* (Pearson correlation r = 0.57, p ≤ 0.05) also correlated with pH (Supplementary Fig. S8). pH was previously described as one of the most influential factors on soil microbial community assemblages^52^. Although the correlation found between soybean RA microbiomes and soil pH does not prove any direct relationship between both observations, it could indicate that this property might be playing also a role at shaping RA communities at field conditions.

Wheat RA microbiome structure correlated with the levels of soil nitrates (Fig. 4C, Mantel Test p ≤ 0.05). However, we found no correlation of nitrates with relative abundances of individual taxa, suggesting that wheat RA microbiomes response to nitrates might occur at a community level rather than at the level of particular taxa. In addition, microbial community assemblies of wheat RA samples were significantly structured by soil type (Supplementary Fig. 10).

We also analyzed the effects of geography on microbiomes assemblies of soybean and wheat. Mantel test detected no correlation between weighted UniFrac distances and geographic distances.

### Culturing the root microbiome

**Figure 5:**
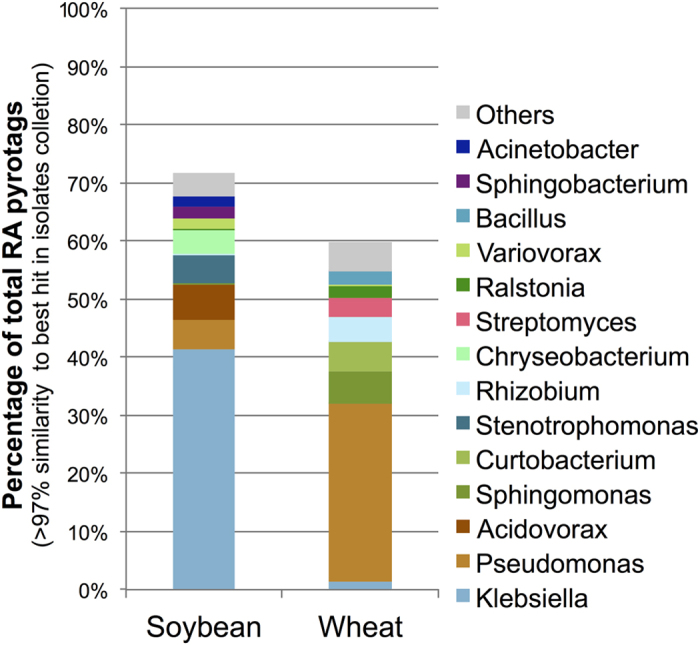
Comparison of metagenomic and cultured bacteria datasets. Root-associated pyrotag reads and Sanger 16 rRNA sequences from rhizospheric isolates (Both amplified with F515-R806 primers) were compared by BlastN. The figure represents the percentage of all root-associated (RA) pyrotags that showed >97% similarity to at least one 16S rRNA sequence of the cultured bacteria. Based on the definition and biological basis of clustering in OTU-97%, we considered these hits as sequences from closely related species, often presenting similar phenotypes, metabolisms and life styles.

The improvement of biotechnological tools to manipulate crop microbiomes will require the isolation of microbial strains (*e.g*.: to be later applied as biofertilizers in native-like soil environments). For this reason, we decided to explore the culturable fraction of the root microbiome using different selective growth media. Since the ultimate goal of this effort was screening for plant growth promoting bacteria, the media used for RS samples were specifically selected for this purpose. The same samples used to extract metagenomic DNA from RA and RS fractions were used as inoculum for bacterial isolation and culturing. We obtained a total of 1,047 isolates from wheat samples and 960 from soybean samples (Supplementary Tables S2 and S3). We amplified and sequenced the V4 hyper variable region of the 16s rRNA genes (*i.e*. the same region used for amplicon sequencing) from each isolate and generated a 16S dataset from isolates. When we compared all pyrotags sequences against the isolate collection, we found that nearly 70% of the reads from soybean RA, and ~60% from wheat RA, got a BlastN hit against an isolated bacteria with more than 97% similarity (Fig. 5). In microbial ecology, sequences with >97% similarity are treated as operational taxonomic units (OTU-97%) and are commonly considered as “close related” species, which may present similar biological properties and potentially play similar roles in microbial communities and the environment^53^.

The taxonomic classification of the isolated collection revealed that we obtained representative strains of a total of 100 different genera (considering only Blast best hits ≥ 90% similarity). We obtained a high proportion of *Pseudomonas* and *Bacillus* genera (representing more than 20% of the full dataset, Supplementary Table S2). The next most represented genera were *Variovorax, Burkholderia* and *Stenotrophomonas* accounting for 5% and 2% of total isolates. Although the isolation of these genera was expected due to the culturing strategies and selective media used in this study, here we generated a collection of isolates that it is composed by strains that are naturally adapted to diverse field conditions and were obtained from rhizospheres. We therefore hypothesize that if these strains were inoculated in field conditions, they would likely interact with crop roots and persist in the environment.

Among the RA isolates, *Pseudomonas* (30%), *Klebsiella* (7%), *Chryseobacterium* (7%), *Variovorax* (5%), *Stenotrophomonas* (5%), *Burkholderia* (4%), *Delftia* (3%), *Acidovorax* (3%), *Pantoea* (2%), *Bacillus* (2%), *Ochrobactrum* (2%) and *Enterobacter* (2%) were more frequently found in soybean isolates (Supplementary Table S2). On the other hand, *Pseudomonas* (58%), *Variovorax* (7%), *Bacillus* (6%), *Stenotrophomonas* (4%), *Herbaspirillum* (3%), *Acidovorax* (2%), *Burkholderia* (2%), *Acinetobacter* (2%) and *Delftia* (1%) were the most abundant genera among the wheat RA isolates.

Our results from both, environmental pyrotags and isolates data, consistently showed that *Acidovorax*, *Burkholderia*, *Chryseobacterium*, *Klebsiella*, *Pseudomonas* and *Stenotrophomonas* were the most ubiquitous and abundant genera associated to soybean roots. Moreover, *Pseudomonas*, *Burkholderia* and *Bacillus* showed a similar pattern in wheat RA microbiomes.

### Plant growth promoting phenotypes in isolated bacteria

Most of the identified taxa in the RA microbiomes are known to contain representative strains with plant growth promoting (PGP) traits. For instance, some *Pseudomonas spp*., *Arthrobacter spp*. and *Bacillus spp*. were shown to produce phyto-stimulation by the release of 1-aminocyclopropane-1-carboxylate (ACC) deaminase that lowers plant hormone ethylene levels^54^,^55^. Moreover, some strains of *Pseudomonas fluorescens*, *Bacillus spp*. and *Rhizobium spp*. were reported to produce cytokines that increase root surface area of crop plants through enhanced formation of lateral and adventitious roots^56^. Plant growth is also promoted by indole-acetic acid (IAA). Some strains of *Rhizobium spp., Bradyrhizobium spp*., *Pseudomonas putida* and *Klebsiella pneumonia, are known to* produce IAA^56^,^57^,^58^. Plant growth promotion can also result from biofertilization, *i.e*., increments in mineral availability as a consequence of microbial activity. Nitrogen fixation and phosphorus solubilization are major metabolisms participating in this process. *Rhizobium spp*. are known nitrogen fixing bacteria in legumes while *Achromobacter spp*. and *Bacillus spp*. have shown to solubilize phosphate^55^.

**Figure 6:**
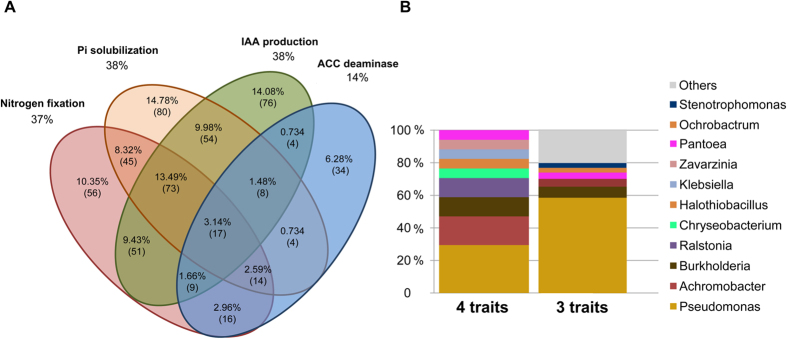
Isolated strains with plant growth promoting (PGP) traits. (**A**) Venn-Diagram showing the isolates that presented each of the possible combinations for the four different PGP evaluated traits. Values are presented in relative abundance (percentage) and as absolute number of isolates (in parenthesis). (**B**) Bars indicate the relative abundance of genera among isolates, which presented four and three different PGP-associated traits *in vitro*.

We then screened the isolates collection for traits that are commonly associated to PGP activities. We screened for two traits associated to phytostimulation: indole-acetic acid (IAA) production and ACC deaminase activity; and two biofertilization traits: nitrogen fixation and phosphorous solubilization^56^,^57^. We evaluated these traits *in vitro* on 757 bacterial isolates. Results showed that 38% of isolates were able to produce IAA, 37% were able to fix nitrogen, 38% could solubilize inorganic phosphorus and 14% had ACC deaminase activity (Fig. 6). Although some of the media used were intended to enrich isolates in certain bacterial groups or properties (*e.g*., sporulating or nitrogen fixing bacteria), for each of the evaluated PGP traits, we were able to obtain several different genera, spanning across groups with different physiological properties and metabolisms^59^.

About 3% of the evaluated isolates presented all four phenotypes. From those, 29% were identified as *Pseudomonas* and 18% as *Achromobacter*. The rest of these isolates were classified as *Burkholderia, Chryseobacterium, Halothiobacillus, Klebsiella, Pantoea, Ralstonia* and *Zavarzinia*. Isolates that showed three of the analyzed traits represented 14% of total isolates, and 59% of them were identified as *Pseudomonas*. The next most abundant genera among this group were *Burkholderia* (6.8%) and *Achromobacter* (4.8%) (Fig. 6B). About 55% of the analyzed isolates presented at least one of the analyzed PGP traits. In summary, we showed that a high proportion of the obtained isolates could potentially be able to improve plant growth *in vivo* by using one or more of the observed PGP traits. We finally analyzed the distribution of isolates with PGP traits by crop and compartment. We observed that bacteria with ACC deaminase, N fixation and IAA production where evenly found among the RA and RS isolates, while phosphate solubilizing bacteria were more abundant in RS isolates (Supplementary Table S5). In the analysis by crop, we found that isolates with ACC deaminase activity were more represented in the soybean collection. Finally, most phosphate solubilizing isolates were found in the wheat RS fraction.

## Discussion

In this work we combined metagenomic approaches with culture methods to characterize the composition and assembly of the root-associated and rhizospheric soil microbiomes of soybean and wheat in agricultural production fields across a large and heterogeneous agricultural area. We showed that diversity variation of these microbiomes correlated with changes in soil environment and that the composition of the RA fraction was more variable in wheat plants than in soybean plants. We also generated a collection of isolates obtained from both crops in production fields and showed that a high proportion of these isolates are promising candidates to be tested as plant growth promoting bacteria in the field.

### Variation of root microbial diversity under field conditions

Alpha and beta diversity analyses revealed that root microbiomes were variable between sampled sites. Sequencing depth was modest in this study and therefore we focused in the characterization of the most abundant and dominant bacteria. Future efforts using other technologies with higher sequencing depth than pyrosequencing will contribute to identify low-abundant and rare species. However, several works have shown that comparative diversity analyses are not significantly affected by sequencing depth^48^,^49^. Moreover, alpha diversity results showed that the RA microbiomes were sufficiently sampled in this study since rarefaction curves were asymptotic in these samples. We also revealed at least a 10-fold difference in OTU richness between RS and RA microbiomes. These results confirm that the same patterns previously observed in *Arabidopsis thaliana* in laboratory controlled conditions are also true in commercial crops in the field^16^,^17^.

As already reported for Arabidopsis and barley^15^,^16^,^17^, we showed that RA microbiomes were highly variable between different plants of the same crop, but the highest differences were found between crops (Fig. 3A). In addition, beta community diversity of the wheat RA fraction was more variable between sampling locations than for the soybean RA fraction (Fig. 3A,B). Although we cannot certainly determine the causes for the variation in wheat RA microbiomes, we can hypothesize that it may be related to root architecture. Wheat root system is flexible, it does not have a vascular cambium, and must lengthen roots to generate new xylem and phloem tissues for water and sugar transport. Wheat may develop up to seven primary roots. Moreover, wheat embryonic and nodal root systems develop over time to re-colonize the soil profile and differ in their responses to soil stimuli. In contrast, soybean has vascular cambia and its root system is much simpler and has a single primary axial root. It also known that soil characteristics and nutrient availability influence root development (Rich and Watt^60^ provide a complete description of this topic)^60^. We thus propose that the differences in soil physical structures and chemical compositions impacted in crops root architecture, with a more significant effect on wheat root development, which resulted in a higher variability of root systems in wheat plants than soybean plants, and this may be therefore reflected in the diversity of the RA microbiota. Since we did not measured root architecture traits in this study, this hypothesis should be properly tested in the future on field conditions.

Large-scale geographic and local edaphic factors are key drivers shaping bulk soil microbiota, which ultimately impart an effect on root microbiota^61^,^62^. These factors can also influence plant physiology and plant–root microbiota interactions. Several works have focused on the influence of soil characteristics on root microorganisms in different cultivars and native plants^17^,^51^,^63^,^64^,^65^,^66^. Here we showed that under field conditions, variation in beta-diversity in soybean RA microbiomes correlated with changes in soil pH. We observed a correlation of pH with the abundance of RA bacteria with known PGP activity such as *Paenibacillus*, *Bradyrhizobium* and *Variovorax*^55^,^56^,^57^,^67^,^68^. Moreover, we observed that soil pH also correlated with soybean and wheat RS microbial community diversity in agreement with previous studies^51^,^69^. It has already been shown that pH is an important driver in bulk soil communities^52^,^70^. These communities are the reservoir for root-associated and soil rhizospheric communities, and therefore changes in their structure and composition are likely to affect root-colonizing microbiota. Bulgarelli et.al have shown that Arabidopsis root endophyte microbiota is influenced by soil characteristics and hypothesized that this is the reflex of the effect of soil environment on native bulk soil microbiota, which later colonize roots^17^. We also adhere to this hypothesis and proposed that bulk soil pH may have a significant effect in soybean root microbiome assemblages in the field. It worth noting that rizhosphere pH is not only modified by plant exudates or local microbiota but it is also affected by bulk soil pH^71^. Furthermore, the buffer capacity of bulk soil is known to regulate rizhosphere pH.

In addition, we observed that the variation in the community structure of wheat RA microbiomes was related with the variation in soil nitrate levels and soil type. The role of soil nitrate in root architecture has been deeply studied^72^. Several studies performed in Arabidopsis thaliana described the mechanism that senses the presence of exogenous nitrate and induces lateral root elongation^73^,^74^. This response to soil nitrate levels has also been described in wheat^75^. It has also been proposed that root architecture can affect root microbiota an viceversa^76^,^77^,^78^. We hypothesize that the correlation observed between wheat RA microbiota diversity and soil nitrate level may be related to changes in the architecture of root systems. Furthermore, the observed correlation between RA community assemblies and soil type in wheat is in agreement with the observed differences in the taxonomic composition. CB and BA root-associated microbiomes have a similar taxonomic profile with Alphaproteobacteria and Actinobacteria as the most dominant groups. The soil classification in these sites is Typic Argiudoll. In contrast, VS and DA communities present taxonomic profiles with Gammaproteobacteria and Planctomycetes as dominant groups. The soil in these sites is classified as Entic Hapludoll. Similar results were previously observed in Arabidopsis RA microbiomes depending on the soil type (loam v. sandy). Moreover, although Actinobacteria was enriched in Arabidopsis RA microbiomes, the abundance of this phylum was shown to affected by soil type, in agreement with our results. Although further work will be needed to better understand the complex interactions between RA microbial communities and soil characteristics, here we showed the first evidences of such association in the field.

### Plant growth promoting bacteria in crop microbiomes

Root microbiomes of crops may be manipulated and optimized in the near future to promote crop productivity^12^,^28^. This goal could be achieved by introducing single microbial strains or even synthetic microbiomes adjusted for each crop and growth condition (e.g., climate, soil properties, season, crop genotype). In any of these cases, culturing of root-interacting microbial strains will be a necessary first step. Moreover, a major obstacle of the current commercially used PGP bacterial strains is that survival rate in the environment is very low, probably because is rapidly replaced by indigenous species^29^,^30^. Some authors suggest that strains that are naturally adapted to a certain environmental condition would have a better fitness and higher survival rate in that environment^29^,^30^. Following this criterion, we attempted to obtain an extensive collection of bacterial strains that normally grow in association or close vicinity of soybean and wheat roots in the field. To do so, we not only targeted a wide range of bacterial species (using rich media), but also used selective culturing strategies to obtain indigenous strains with known PGP activities (such as *Pseudomonas*, *Bacillus* and nitrogen fixing bacteria). We obtained a vast collection of 2,007 isolates from wheat and soybean plants growing in different environmental conditions. Moreover, we showed that this collection is likely a good representation of the dominant root bacterial diversity revealed by pyrotag sequencing.

In addition we found that a significant proportion of these isolates showed multiple PGP activities *in vitro*. Most of these strains were classified as *Pseudomonas, Achromobacter, Klebsiella, Stenotrophomonas, Chryseobacterium* and *Bacillus*. It was previously shown that representatives of these genera have beneficial effects on plant growth and health^54^,^56^,^57^,^68^. Most importantly, our pyrotag analyses indicated that these groups were among the most abundant and ubiquitous in the root-associated fraction under field conditions. All together our results suggest that the obtained collection of isolates contain a good representation of the bacterial community that interact with soybean and wheat roots in field conditions. The PGP associated properties found *in-vitro* also suggest that there are several strains in this collection that could potentially enhance crop growth. Future work will be destined to test experimentally the PGP activity of these strains in production field trials.

### Final remarks

Microbiomes are highly complex biological entities and the link between crop microbiomes and crop productivity require further investigation. Here we showed that combining culture independent and culture dependent high-throughput analyses could successfully lead to a better understanding of crop microbiomes and the exploration of novel approaches for favoring crop growth. We identified soil properties that may contribute to root microbiome assemblages in agricultural fields, we characterized the distribution of root-associated bacterial taxa in an environmental scale and we identified the dominant groups that associate with each crop. We determined that a many members of the observed environmental communities are closely related to the isolated strains; we also verified that many of this isolates presented PGP-associated activities *in vitro*. Moreover, we were able to identify specific bacterial strains that not only presented all four evaluated PGP traits, but also were among the dominant taxa under field conditions. All together, these results represent a significant step forward in our understanding of crop microbiomes and open the gate for future efforts in developing better agricultural practices by crop microbiome manipulation in the environment.

## Additional Information

**How to cite this article**: Rascovan, N. *et al*. Integrated analysis of root microbiomes of soybean and wheat from agricultural fields. *Sci. Rep*. **6**, 28084; doi: 10.1038/srep28084 (2016).

## Supplementary Material

Supplementary Information

Supplementary Text
