# Supplementary material for: Integrated analysis of root microbiomes of soybean and wheat from agricultural fields
Source: Sci Rep. 2016 Jun 17;6:28084. doi: 10.1038/srep28084 (PMC4911569; doi:10.1038/srep28084)
Supplement: Supplementary Information [file srep28084-s1.pdf]

## Supplementary information:

# Integrated analysis of root microbiomes of soybean and wheat from agricultural fields

Nicolás Rascovan*, Belén Carbonetto*, Diego Perrig, Marisa Díaz, Wilter Canciani, Matías Abalo, Julieta Alloati, Gustavo González Anta & Martín P. Vazquez.

## Supplementary methods:

### PGP traits detection

*IAA*

Isolates were cultured on nutrient agar medium and overlaid with cellulose membrane and incubated for 48h (Bric et al 1991). Salkowski's reagent was added on the cellulose membrane after 48h of incubation. Pink coloration indicated production of IAA (Supplementary Figure S2A).

*ACC deaminase*

Isolates were screened for their ability to utilize ACC as the sole nitrogen source in DF minimal medium according to the method of Dell'Amico et al. (Dell'Amico et al 2005). Three culture conditions were used: DF minimal medium alone as a negative control; DF minimal medium plus $(NH_4)_2SO_4$ (2g $L^{-1}$) as a positive control; and DF minimal medium plus 3 mmol $L^{-1}$ ACC as the selective medium (Supplementary Figure S2B).

*P solubilazation*

Phosphorous solubilization was detected using the Pikovskaya's agar medium. In brief, positive strains are capable of producing a halo/clear zone due to the release of organic acids into the surrounding medium (Supplementary Figure S2C) (Katznelson et al 1962).

*Nitrogen fixation*

Nitrogen fixation was evaluated in 5ml of N-free semisolid medium in 10 ml vials. The inoculation was done into the center of the medium. In general terms, the principle of this semi-solid medium is that it allows for growth of the bacteria under conditions where their nitrogenase requires protection from O2-mediated denaturation (Dalton and Postgate 1968) *i.e.* in early stages of growth when only a small number of cells are starting to multiply and they thus use little oxygen. In later stages, the bacterial cells within the culture medium migrate closer to the surface where there is sufficient oxygen pressure to support aerobic respiration, but not so much that it damages nitrogenase. In this stage a sub-surface growth pellicle is observed. The presence of absence of the pellicle defines the ability to fix nitrogen (i.e. nitrogenase activity, Supplementary Figure S2D) (Baldani et al 2014).

**F515-R806 primers bias in rhizospheric soil samples**

We observed that 1.5-2.5% reads of the RS fraction were classified as Proteobacteria in rhizospheric soil samples (Supplementary Figure S8). However it has been reported that Proteobacteria usually accounts for over 20% relative abundance in rhizospheric soil communities (Mendes et al 2011, Peiffer et al 2013). Although we have not identified yet the source of this bias, the only methodological difference respect to other published works is the use of a particular TAG sequence (TAG-F 5'-CACGACGTTGTAAAACGAC-3', TAG-R 5'-CAGGAAACAGCTATGACC-3') in the F515- R806 primers (Caporaso et al 2011b). We are aware that this bias was never reported before, but unfortunately, it affected several different pyrosequencing runs, with different sample types (i.e. soil, root tissue, human samples) and only when this combination of primers and TAG was used (data not shown). We hypothesize that this effect was not observed in RA datasets because they are highly dominated by Proteobacteria so even with poor primer hybridization they were successfully amplified. Based on these results, we decided to use RS samples only for diversity analyses, where we considered that the observed richness is equal or most likely lower than it would have been measured with a better Proteobacteria representation.

**Bibliography:**

Baldani JI, Reis VM, Videira SS, Boddey LH, Baldani VLD (2014). The art of isolating nitrogen-fixing bacteria from non-leguminous plants using N-free semi-solid media: a practical guide for microbiologists. *Plant Soil* **384:** 413-431.

Bric JM, Bostock RM, Silverstone SE (1991). Rapid in situ assay for indoleacetic Acid production by bacteria immobilized on a nitrocellulose membrane. *Appl Environ Microbiol* **57:** 535-538.

Caporaso JG, Lauber C, Costello E, Berg-Lyons D, Gonzalez A, Stombaugh J *et al* (2011a). Moving pictures of the human microbiome. *Genome Biology* **12:** R50.

Caporaso JG, Lauber CL, Walters WA, Berg-Lyons D, Lozupone CA, Turnbaugh PJ *et al* (2011b). Global patterns of 16S rRNA diversity at a depth of millions of sequences per sample. *Proceedings of the National Academy of Sciences of the United States of America* **108 Suppl 1:** 4516-4522.

Caporaso JG, Lauber CL, Walters WA, BergLyons D, Huntley J (2012). Ultra-high-throughput microbial community analysis on the Illumina HiSeq and MiSeq platforms. *ISME J*: 1621-1624.

Dalton H, Postgate JR (1968). Effect of oxygen on growth of Azotobacter chroococcum in batch and continuous cultures. *Journal of general microbiology* **54:** 463-473.

Dell'Amico E, Cavalca L, Andreoni V (2005). Analysis of rhizobacterial communities in perennial Graminaceae from polluted water meadow soil, and screening of metal-resistant, potentially plant growth-promoting bacteria. *FEMS Microbiol Ecol* **52:** 153-162.

He Y, Zhou B-J, Deng G-H, Jiang X-T, Zhang H, Zhou H-W (2013). Comparison of microbial diversity determined with the same variable tag sequence extracted from two different PCR amplicons. *BMC Microbiology* **13:** 208.

Katznelson H, Peterson EA, Rouatt JW (1962). Phosphate-dissolving microorganisms on seed and in the root zone of plants. *Canadian Journal of Botany* **40:** 1181-1186.

Mendes R, Kruijt M, de Bruijn I, Dekkers E, van der Voort M, Schneider JHM *et al* (2011). Deciphering the Rhizosphere Microbiome for Disease-Suppressive Bacteria. *Science* **332:** 1097-1100.

Peiffer JA, Spor A, Koren O, Jin Z, Tringe SG, Dangl JL *et al* (2013). Diversity and heritability of the maize rhizosphere microbiome under field conditions. *Proceedings of the National Academy of Sciences* **110:** 6548-6553.

Rascovan N, Carbonetto B, Revale S, Reinert M, Alvarez R, Godeas A *et al* (2013). The PAMPA datasets: a metagenomic survey of microbial communities in Argentinean pampean soils. *Microbiome* **1:** 21.

**Supplementary figures:**

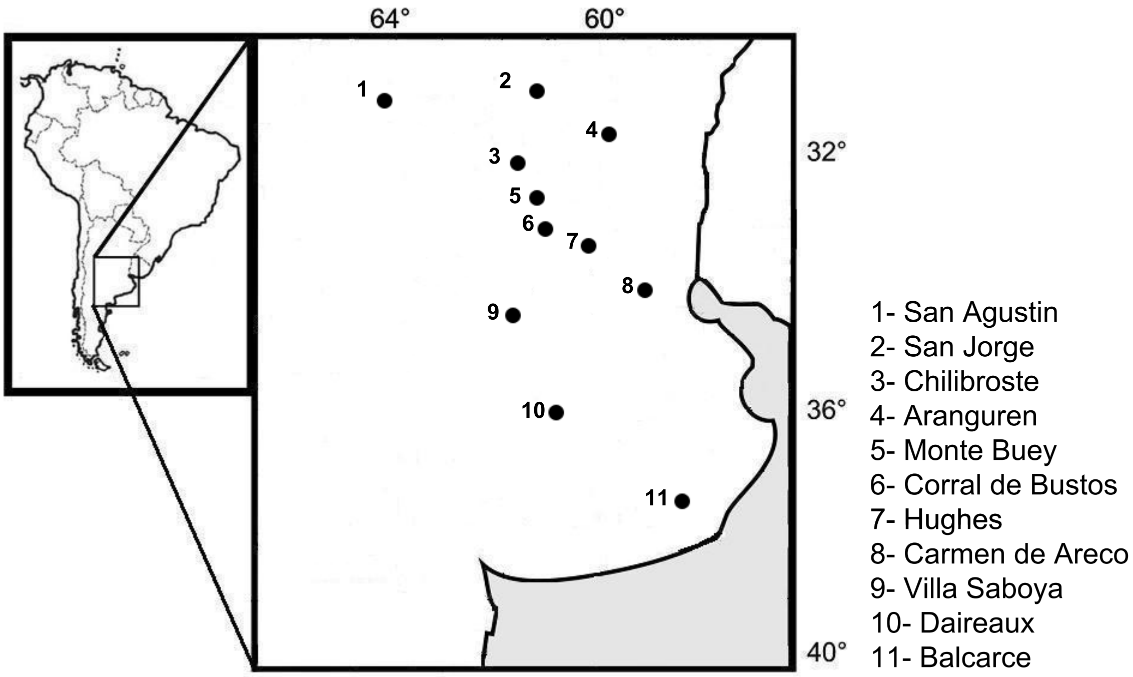


**Supplementary Figure S1: Sampling locations.** Samples were taken in experimental fields from 11 different locations in Argentine Pampas. The GIS coordinates to build the maps were obtained from DIVA-GIS service (http://www.diva-gis.org/) and imported to gvGIS software (Version: 2.2.0-2313) for visualization (http://www.gvsig.com/). The obtained PDF figures for each map were edited in Adobe Photoshop cs5 (https://www.adobe.com) for final figure formatting.

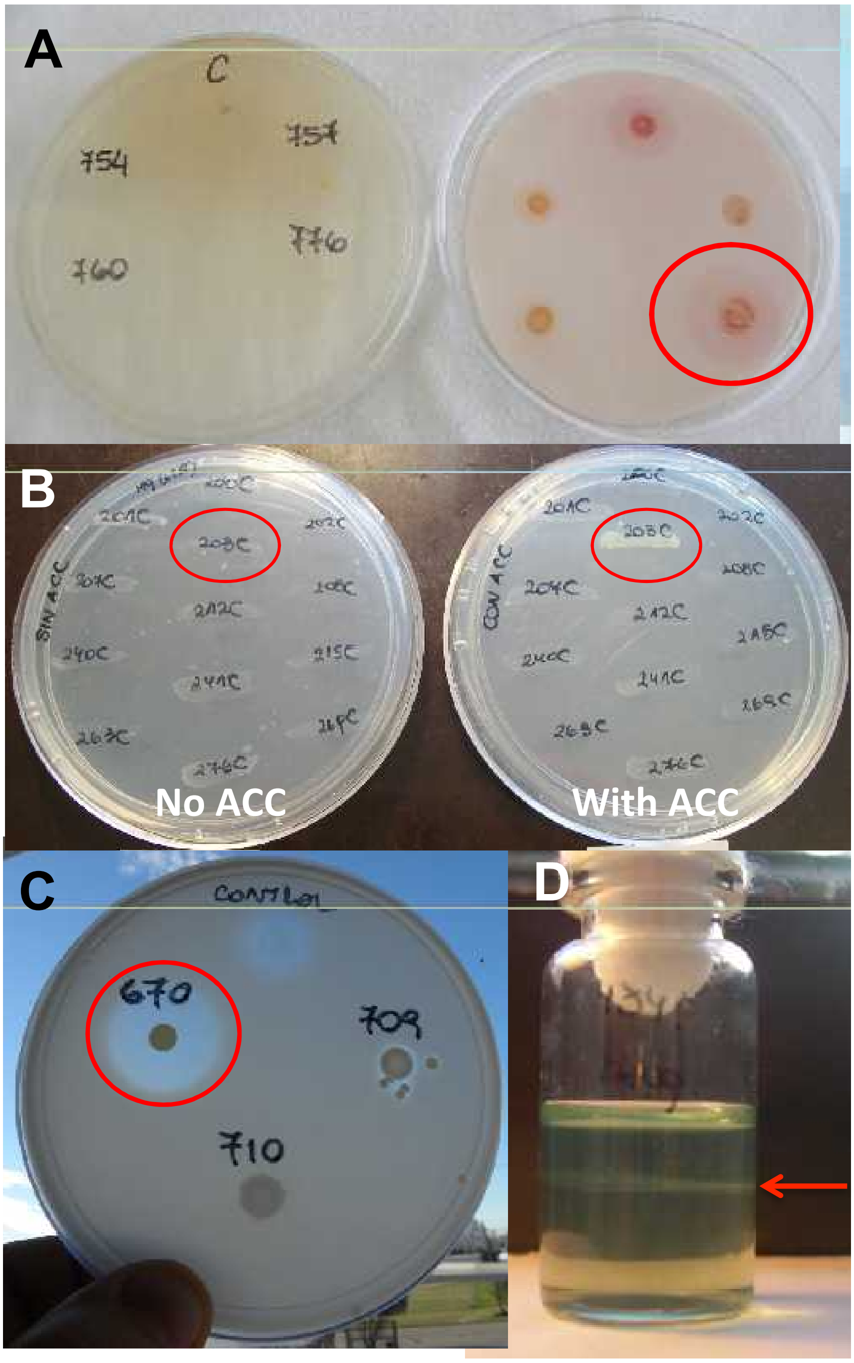


**Supplementary Figure S2: Examples of isolates with positive activities for the four PGP traits**. A) Pink coloration indicates production of IAA. B) Growth on ACC selective medium indicates that ACC can be used as a source of nitrogen. C) Strains that are capable of producing a halo/clear zone in the Pikovskaya's agar medium, are able to solubilize P due to the release of organic acids into the surrounding medium D) Since nitrogenase is sensitive to oxygen, the presence of a pellicle at the sub-surface (aerobic region but with lower oxygen pressure) of the N-free semisolid medium indicate the ability to fix nitrogen.

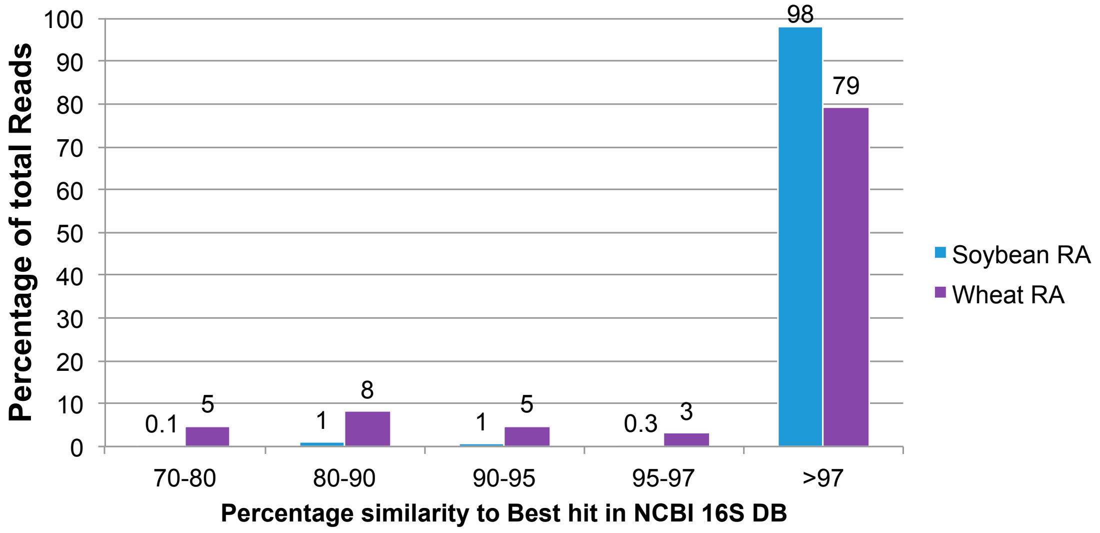


**Supplementary Figure S3: Percentage similarity of best blast hits of pyrotag RA data.** All root-associated soil pyrotags were blasted against NCBI 16S rRNA database and then grouped according to percentage similarity intervals to best hit.

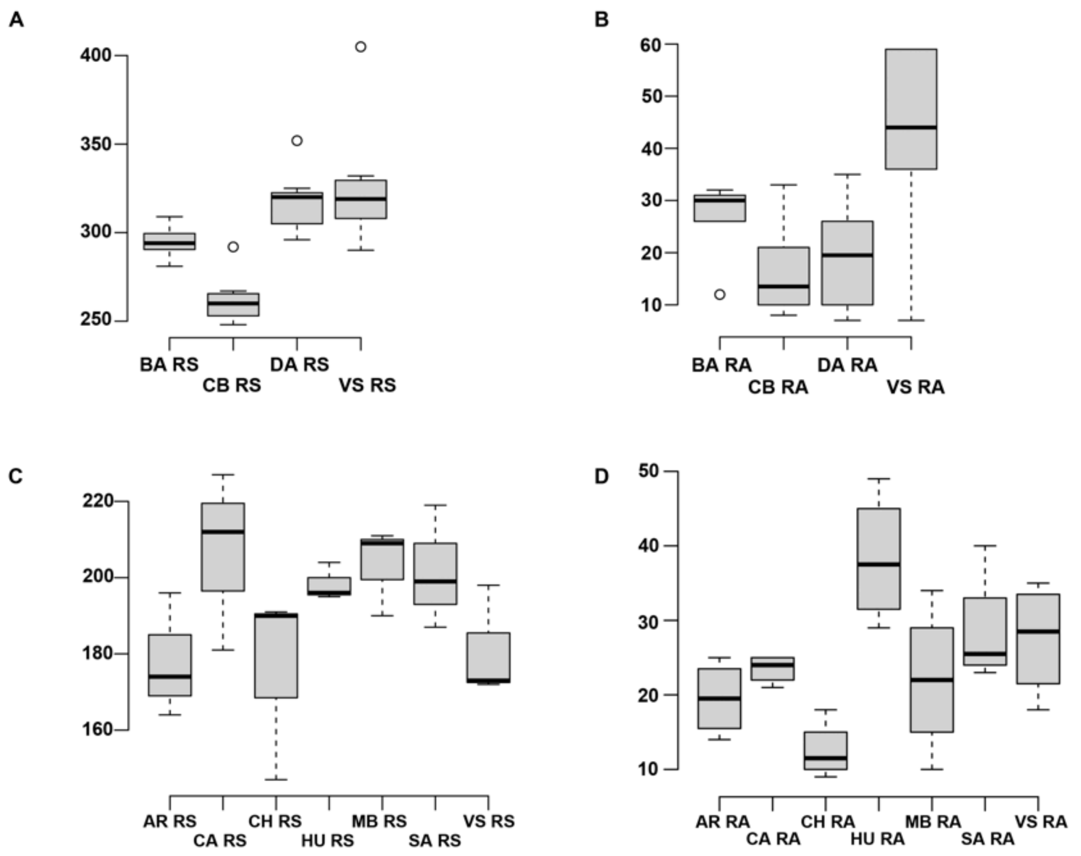


**Supplementary Figure S4: Comparison of alpha diversity between sampling sites.** Boxplots represent OTU richness. Kruskal-Wallis Test was used to detect differences in wheat RS mirobiomes (A), wheat RA microbiomes (B), soybean RS mirobiomes (C) and soybean RA microbiomes (D). Sampling sites: AR: Aranguren, CA: Carmen de Areco, CH: Chilibroste, HU: Huges, MB: Monte Buey, SA: San Agustin, VS: Villa Saboya, BA: Balcarce, CB: Corral de Bustos, DA: Daireaux. Significant differences were found for soybean RS and RA microbiomes and for wheat RS microbiomes ($p<0.05$).

# Soybean root-associated bacteria observed by pyrotags

| Site | | | | | | | | | | | | | | | | | | | | | | | | | | | | | | | Taxonomy | | | | | |
|---|---|---|---|---|---|---|---|---|---|---|---|---|---|---|---|---|---|---|---|---|---|---|---|---|---|---|---|---|---|---|---|---|---|---|---|---|
| Aranguren | | | | Carmen de Areco | | | | Corral de Bustos | | | Chiribroste | | | | Huges | | | | Monte Buey | | | | San Agustin | | | | Villa Saboya | | | | Domain | Phylum | Class | Order | Family | Genus |
| 49.61 | 39.80 | 62.09 | 76.52 | 22.58 | 31.66 | 31.17 | 42.51 | 45.12 | 39.66 | 28.94 | 78.63 | 67.18 | 12.14 | 45.99 | 38.98 | 28.49 | 31.32 | 51.07 | 46.83 | 18.56 | 15.50 | 73.81 | 62.37 | 59.40 | 63.57 | 38.62 | 50.55 | 60.04 | 45.10 | 64.42 | Bacteria | Proteobacteria | Gammaproteobacteria | Enterobacteriales | Enterobacteriaceae | Klebsiella |
| 8.23 | 4.44 | 2.90 | 1.01 | 25.58 | 18.06 | 36.35 | 12.97 | 45.86 | 39.66 | 37.73 | 17.44 | 3.63 | 3.54 | 5.32 | 5.53 | 0.59 | 10.50 | 4.76 | 49.63 | 8.51 | 58.96 | 9.91 | 4.04 | 1.20 | 0.76 | 4.96 | 15.03 | 20.15 | 22.90 | 1.14 | Bacteria | Proteobacteria | Gammaproteobacteria | Pseudomonadales | Pseudomonadaceae | Pseudomonas |
| 11.53 | 28.37 | 20.37 | 14.57 | 4.02 | 7.65 | | 1.20 | 3.41 | 0.41 | 3.92 | 0.79 | 5.23 | 1.74 | 10.23 | 2.01 | 1.12 | 0.31 | 0.64 | 0.83 | 0.85 | 3.43 | 0.18 | 5.60 | 12.60 | 5.10 | 3.22 | 5.37 | 5.50 | 9.37 | 9.96 | Bacteria | Proteobacteria | Gammaproteobacteria | Xanthomonadales | Xanthomonadaceae | Stenotrophomonas |
| 1.18 | 1.05 | 0.57 | 1.01 | 0.17 | 0.47 | 4.66 | 0.07 | 0.09 | 0.68 | 0.63 | 0.50 | 0.07 | 0.48 | 0.13 | 0.56 | 0.56 | 0.34 | 0.18 | 0.64 | 0.26 | 0.35 | | 0.24 | 0.40 | 0.13 | 1.22 | 1.41 | 0.45 | 0.80 | 0.71 | Bacteria | Proteobacteria | Alphaproteobacteria | Rhizobiales | Rhizobiaceae | Rhizobium |
| 3.08 | 0.23 | 3.32 | | 7.44 | | 0.46 | 12.90 | 0.46 | 2.64 | 9.73 | | 10.99 | 0.17 | | 0.42 | 0.92 | 0.65 | 0.11 | | 1.71 | 0.03 | 0.53 | | 2.90 | 0.64 | 0.22 | 1.25 | 0.24 | 3.83 | 0.24 | Bacteria | Proteobacteria | Gammaproteobacteria | Pseudomonadales | Moraxellaceae | Acinetobacter |
| 7.22 | | | 0.20 | 8.13 | 9.88 | 7.35 | 0.67 | | | 5.33 | | 5.42 | 4.24 | | 10.86 | 3.47 | 7.11 | 7.34 | | 0.76 | 11.83 | 1.24 | 1.27 | 2.40 | 6.11 | 2.66 | 6.18 | 3.30 | 1.26 | 3.90 | Bacteria | Bacteroidetes | Flavobacteriia | Flavobacteriales | Flavobacteriaceae | Chryseobacterium |
| 5.88 | 1.05 | 2.62 | | 13.60 | 12.92 | 9.71 | 16.78 | | 1.97 | 0.94 | | 1.72 | 21.66 | 33.96 | 0.92 | 10.51 | 0.22 | 9.63 | 0.05 | 0.36 | 0.14 | 8.67 | | | | 0.07 | | 0.79 | 0.06 | | Bacteria | Proteobacteria | Betaproteobacteria | Burkholderiales | Comamonadaceae | Acidovorax |
| 2.35 | 9.48 | 2.62 | | | 0.95 | 0.72 | | | | 0.16 | | 2.39 | 0.72 | 0.41 | 0.92 | 0.06 | 2.38 | 0.32 | 0.05 | | | 0.88 | 0.10 | 0.30 | 0.51 | | 2.51 | 1.79 | | 6.89 | Bacteria | Proteobacteria | Betaproteobacteria | Burkholderiales | Alcaligenaceae | Achromobacter |
| 0.11 | 1.35 | 1.13 | | 0.09 | | 0.07 | 0.13 | | | 0.16 | 0.14 | 0.07 | 0.29 | 0.03 | 0.18 | | | | 0.92 | 0.46 | 0.59 | | 0.10 | 0.20 | | 0.11 | 0.04 | | 0.34 | 0.31 | Bacteria | Proteobacteria | Alphaproteobacteria | Rhizobiales | Rhizobiaceae | Agrobacterium |
| 0.17 | 0.30 | | 0.20 | | 0.07 | 0.07 | | | 0.07 | | | | | 0.13 | 0.11 | 1.51 | 0.17 | 1.00 | 0.09 | 0.03 | 0.07 | 1.06 | | 0.20 | 0.13 | | 0.12 | 0.03 | 0.37 | 0.31 | Bacteria | Proteobacteria | Betaproteobacteria | Burkholderiales | Burkholderiaceae | Burkholderia |
| 0.11 | | 0.14 | | 0.09 | 0.14 | | | | 0.07 | | 0.14 | 0.04 | 0.02 | | 0.04 | 0.31 | 0.02 | 0.29 | | 0.07 | 0.42 | | 0.05 | 0.30 | 0.13 | 0.11 | 0.16 | | 0.06 | 0.04 | Bacteria | Proteobacteria | Alphaproteobacteria | Rhizobiales | Brucellaceae | Ochrobactrum |
| 6.94 | 9.78 | | 0.20 | 6.33 | 11.71 | 2.95 | 0.27 | | | 0.24 | | | | | 2.43 | 1.48 | 0.96 | 1.04 | | 0.85 | | | 0.58 | 2.40 | | 2.77 | 6.34 | 3.09 | 5.91 | 3.50 | Bacteria | Bacteroidetes | Sphingobacteriia | Sphingobacteriales | Sphingobacteriaceae | Sphingobacterium |
| | | | | | | 0.07 | | | | | 0.07 | | 47.37 | | 0.70 | 24.52 | 31.08 | 0.57 | 0.14 | 57.80 | 0.21 | 0.53 | 10.37 | 0.40 | 0.13 | 7.18 | 0.12 | 0.07 | 0.52 | 0.04 | Bacteria | Verrucomicrobia | Spartobacteria | Unclassified order | Unclassified family | Chthoniobacter |
| | | 0.21 | | 0.86 | 0.34 | 1.12 | 0.60 | 0.18 | 0.47 | | 0.50 | 0.49 | 0.32 | | 0.07 | 0.06 | 0.05 | 0.04 | | 0.07 | | | 0.05 | | | 0.07 | | 0.03 | | 0.51 | Bacteria | Firmicutes | Bacilli | Bacillales | Paenibacillaceae | Paenibacillus |
| 0.39 | | 0.07 | | 0.43 | 0.14 | 2.89 | 0.74 | 0.46 | | 2.98 | 1.50 | | | 2.61 | | 1.15 | | | | | | | 0.54 | 0.20 | 4.20 | 0.74 | | 0.93 | 0.12 | 1.26 | Bacteria | Proteobacteria | Gammaproteobacteria | Xanthomonadales | Xanthomonadaceae | Dyella |
| 0.06 | | 0.42 | | 0.86 | 0.07 | 0.52 | 0.87 | 1.10 | 3.93 | 1.25 | | 0.41 | 0.21 | 0.63 | 0.11 | 0.11 | | 0.11 | | | 0.14 | 0.88 | | | | | | | | 0.04 | Bacteria | Firmicutes | Clostridia | Clostridiales | Lachnospiraceae | Lachnoclostridium |
| 0.06 | | | | 0.60 | 0.27 | | 1.00 | | | | | | 0.12 | 0.06 | 0.63 | 0.78 | 0.17 | 0.68 | | 0.07 | 0.80 | | 0.15 | | 0.76 | 1.33 | 0.08 | | 0.12 | 0.12 | Bacteria | Bacteroidetes | Sphingobacteriia | Sphingobacteriales | Sphingobacteriaceae | Mucilaginibacter |
| 0.34 | 0.90 | | | | | 0.07 | | | | | | | 0.62 | | 1.90 | 0.50 | 0.10 | 2.08 | | 0.16 | 0.07 | | 0.05 | 0.10 | | 0.85 | 0.97 | 1.17 | 0.52 | 0.47 | Bacteria | Proteobacteria | Alphaproteobacteria | Sphingomonadales | Sphingomonadaceae | Novosphingobium |
| 0.34 | 0.23 | 0.14 | | 0.26 | 0.20 | | | | | | | | 1.40 | | 0.46 | 0.31 | 0.31 | | | 0.07 | 0.21 | | | | 0.51 | 0.07 | 0.24 | 0.24 | 0.43 | 1.65 | Bacteria | Proteobacteria | Alphaproteobacteria | Caulobacterales | Caulobacteraceae | Caulobacter |
| | | 0.21 | 0.40 | 0.17 | 0.14 | 0.13 | 0.20 | | | | 0.04 | | | | | 0.03 | 0.58 | 0.07 | | | 0.03 | 0.35 | 0.34 | | 0.51 | 0.44 | 0.40 | | 2.05 | | Bacteria | Planctomycetes | Planctomycetia | Planctomycetales | Planctomycetaceae | Singulisphaera |
| 0.50 | 0.15 | 0.35 | | 0.17 | | 0.07 | 0.40 | 0.09 | | | | | 0.05 | | 0.18 | 0.03 | 0.17 | | | 0.03 | | | 0.29 | | 0.38 | 0.67 | 0.69 | | 0.03 | | Bacteria | Bacteroidetes | Sphingobacteriia | Sphingobacteriales | Chitinophagaceae | Chitinophaga |
| | 0.90 | | | | 0.20 | | | | 0.14 | | | | 0.19 | | 0.14 | 0.03 | 0.07 | 0.14 | | 0.13 | 0.45 | 0.35 | | | 0.13 | 0.22 | 0.12 | 0.10 | 0.15 | 0.16 | Bacteria | Proteobacteria | Betaproteobacteria | Burkholderiales | Burkholderiaceae | Cupriavidus |
| 0.06 | | | | | | 0.07 | | | | | | 0.04 | | 0.03 | 0.32 | 0.06 | 0.41 | 0.04 | 0.18 | 0.23 | 0.14 | 0.18 | 0.10 | 0.10 | 0.13 | 0.15 | | | 0.24 | | Bacteria | Acidobacteria | unclassified Acidobacteria | Acidobacteria subdivision 4 | Unclassified family | Other |
| 0.34 | 0.08 | | | | | | | | | | | | 0.53 | | 0.14 | 0.39 | 0.12 | 0.29 | | 0.23 | 0.21 | | 0.19 | | 0.89 | 1.33 | 0.48 | 0.10 | 0.15 | 0.47 | Bacteria | Proteobacteria | Alphaproteobacteria | Sphingomonadales | Sphingomonadaceae | Sphingomonas |
| | 0.15 | | | 1.63 | 0.47 | | | | | | | | 0.24 | | 0.32 | 0.06 | 0.02 | | | 0.10 | 0.07 | | 0.19 | | 0.13 | 0.07 | 0.61 | 0.03 | 0.15 | 0.16 | Bacteria | Proteobacteria | Betaproteobacteria | Burkholderiales | Comamonadaceae | Variovorax |
| | | | | | 1.56 | | | 2.39 | 8.27 | 3.92 | | 2.06 | 0.53 | | 0.85 | | 0.79 | 0.36 | | 2.10 | | | 10.52 | 12.70 | 9.68 | 10.17 | 0.32 | | | | Bacteria | Proteobacteria | Betaproteobacteria | Burkholderiales | Comamonadaceae | Delftia |
| 0.34 | 0.53 | 0.21 | 1.42 | 0.17 | 0.20 | | | | | | | 0.15 | 0.36 | | 0.11 | 0.31 | 0.75 | 0.43 | | | | | | 0.10 | | | | 0.07 | | 0.35 | Bacteria | Proteobacteria | Betaproteobacteria | Burkholderiales | Oxalobacteraceae | Herbaspirillum |
| | 0.07 | | | | | 0.07 | | | | | | | | 0.03 | 0.18 | 0.06 | 0.24 | 0.04 | 0.09 | 0.26 | 0.10 | | 0.24 | 0.10 | | 0.11 | | | 0.09 | | Bacteria | Acidobacteria | unclassified Acidobacteria | Acidobacteria subdivision 4 | Unclassified family | Blastocatella |
| 0.22 | | | | 3.42 | 1.76 | | 7.75 | | | | | | 0.02 | | 0.35 | | | 0.07 | | | | | | 0.10 | 0.13 | 0.04 | | 0.58 | 0.03 | 0.08 | Bacteria | Proteobacteria | Alphaproteobacteria | Rhizobiales | Bradyrhizobiaceae | Bradyrhizobium |
| | | | | | | | | 0.09 | 0.07 | 0.08 | 0.07 | | | 0.06 | 0.21 | 1.98 | | | 0.18 | | 0.03 | 0.53 | | | | | | 0.03 | 0.03 | 1.02 | Bacteria | Proteobacteria | Betaproteobacteria | Burkholderiales | Burkholderiaceae | Ralstonia |
| 0.22 | | | 0.40 | 0.51 | | | | | | | 0.07 | 0.04 | 0.70 | | 0.14 | 0.06 | 0.41 | 0.21 | | | | | | | 0.76 | 0.44 | | 0.28 | | | Bacteria | Proteobacteria | Alphaproteobacteria | Rhodospirillales | Rhodospirillaceae | Azospirillum |
| | | | 0.20 | | | | | | 0.07 | | | | | | 0.32 | 0.03 | 0.14 | 0.07 | | 0.43 | | | 0.05 | | | 0.07 | 0.04 | 0.07 | 0.21 | 0.08 | Bacteria | Planctomycetes | Planctomycetia | Planctomycetales | Other | Other |
| 0.17 | | | 0.20 | 0.09 | | | 0.40 | | | | | | | | 0.11 | 0.36 | 0.41 | | | | 0.07 | | | 0.10 | 0.13 | 0.07 | | 0.03 | | | Bacteria | Proteobacteria | Betaproteobacteria | Burkholderiales | Oxalobacteraceae | Duganella |
| | | | | | 0.07 | | | | | | | | 0.10 | | 0.07 | 0.14 | 0.26 | 0.11 | | | 0.14 | | 0.05 | | 0.25 | 0.07 | 0.08 | | 0.12 | | Bacteria | Proteobacteria | Betaproteobacteria | Burkholderiales | Oxalobacteraceae | Massilia |
| | | | | 0.09 | 0.27 | | | | | | | | 0.03 | | 0.18 | | 0.02 | 0.04 | | 0.16 | | | 0.05 | | | 0.04 | 0.20 | | 0.03 | 0.04 | Bacteria | Proteobacteria | Alphaproteobacteria | Rhizobiales | Bradyrhizobiaceae | Bosea |
| 0.06 | 0.08 | | | | 0.14 | | | | | | | | | | 0.07 | 0.03 | 0.02 | 0.04 | | 0.03 | | | 0.19 | | | 0.04 | 0.20 | | | 0.08 | Bacteria | Proteobacteria | Alphaproteobacteria | Rhizobiales | Bradyrhizobiaceae | Tardiphaga |
| | | | | | | 0.07 | | | | | | | | | 0.11 | 0.03 | 0.10 | 0.04 | | 0.13 | 0.03 | 0.18 | | | | 0.11 | 0.04 | | 0.03 | 0.04 | Bacteria | Planctomycetes | Phycisphaerae | Phycisphaerales | Other | Other |
| 0.06 | | | | 0.17 | | | | | | | | | 0.60 | | | | 0.07 | | | 0.59 | 0.07 | | | | 1.02 | 0.04 | 0.36 | | 0.03 | 0.59 | Bacteria | Proteobacteria | Alphaproteobacteria | Sphingomonadales | Sphingomonadaceae | Sphingopyxis |
| | | | | | | 0.07 | 0.07 | | | | | | | | 0.14 | 0.14 | 0.10 | | | | | | 0.10 | | | 9.84 | 0.08 | | 0.12 | 0.04 | Bacteria | Planctomycetes | Planctomycetia | Planctomycetales | Planctomycetaceae | Other |
| | | | | | | | | 0.46 | | 1.57 | | | | 0.06 | 1.23 | 0.03 | | | | | 0.31 | | | 1.40 | 2.42 | | 0.04 | | 1.44 | | Bacteria | Proteobacteria | Gammaproteobacteria | Enterobacteriales | Enterobacteriaceae | Serratia |
| 0.06 | | | | | | | | 0.09 | | | | | | | 0.11 | | 0.07 | | | | 0.03 | 0.35 | 0.19 | | | 0.11 | 0.48 | | 0.12 | | Bacteria | Actinobacteria | Actinobacteridae | Actinomycetales | Micrococcineae | Cellulomonas |
| | | | | | | | | | | | | | | | 0.07 | | 0.10 | 0.04 | 0.14 | 0.13 | | | 0.05 | 0.10 | | | | 0.03 | 0.21 | 0.04 | Bacteria | Proteobacteria | Deltaproteobacteria | Syntrophobacterales | Syntrophaceae | Other |
| | | | | | | | | | | | | | | | 1.34 | 2.29 | 3.30 | 1.15 | | 0.43 | | | 0.83 | 1.30 | | 6.33 | | | | 0.59 | Bacteria | Bacteroidetes | Flavobacteriia | Flavobacteriales | Flavobacteriaceae | Flavobacterium |
| | | | 1.82 | 1.63 | 0.14 | | 0.20 | | 0.27 | | | | | | | | | | | 0.20 | 4.76 | | | | | 0.92 | | | 1.04 | | Bacteria | Proteobacteria | Betaproteobacteria | Burkholderiales | Alcaligenaceae | Bordetella |
| | | | | | | | 0.07 | | | | | | | 0.03 | 0.11 | 0.08 | 0.12 | | | 0.10 | | | 0.05 | 0.10 | | | 0.04 | | | | Archaea | Thaumarchaeota | Nitrososphaerales | Nitrososphaerales | Nitrososphaeraceae | Nitrososphaera |
| 0.11 | 0.45 | 1.06 | 1.21 | | 0.14 | | | | | 0.16 | | | | | | | | | | | | | 0.05 | | 0.25 | | | | | | Bacteria | Proteobacteria | Gammaproteobacteria | Xanthomonadales | Xanthomonadaceae | Xanthomonas |
| | | | | 0.60 | 0.07 | | | | | | | | 0.05 | | | 0.03 | 0.12 | | | | | | 0.10 | | | 0.15 | | | | 0.16 | Bacteria | Proteobacteria | Betaproteobacteria | Methylophilales | Methylophilaceae | Methylophilus |
| | | | | | | | | | | | | | | | 0.04 | 0.03 | 0.07 | | | 0.03 | | | | | | 0.04 | 0.08 | | 0.06 | 0.08 | Bacteria | Proteobacteria | Alphaproteobacteria | Rhizobiales | Phyllobacteriaceae | Mesorhizobium |
| | 0.08 | | | | | | | | | | | | | 0.03 | 0.04 | 0.03 | 0.05 | | | 0.07 | | | | | | | 0.08 | | 0.06 | | Bacteria | Armatimonadetes | Chthonomonadetes | Chthonomonadales | Chthonomonadaceae | Other |
| | | | | | | | | | | 2.20 | | | | | 0.18 | 15.85 | | 0.07 | | | | | | 0.30 | | | 0.44 | 0.45 | | | Bacteria | Proteobacteria | Betaproteobacteria | Burkholderiales | Comamonadaceae | Comamonas |
| | | | | | | | | | | | | | | | 0.14 | | 0.17 | | | 0.07 | 0.07 | | 0.05 | | | | 0.04 | | 0.18 | | Bacteria | Proteobacteria | Alphaproteobacteria | Rhodospirillales | Rhodospirillaceae | Other |
| | | | | | | | | | | | | | | | 0.11 | | 0.02 | | | | | | 0.10 | | | 0.22 | 0.08 | | 0.03 | 0.08 | Bacteria | Proteobacteria | Alphaproteobacteria | Caulobacterales | Caulobacteraceae | Phenylobacterium |
| | | | 0.20 | | | | | | | | | | | | 0.04 | | 0.02 | | 0.09 | 0.03 | | | | | | | | | 0.09 | 0.04 | Bacteria | Firmicutes | Bacilli | Bacillales | Other | Other |
| | | | | | | | | | | | | | | | 0.04 | | 0.02 | | 0.05 | 0.03 | 0.03 | | | | | | 0.04 | | 0.09 | | Bacteria | Firmicutes | Clostridia | Thermoanaerobacterales | Thermoanaerobacteraceae | Other |
| | | | | | | 0.20 | | | | | | | | | 0.88 | | | 0.18 | | 1.48 | | | 0.05 | | | | | | 0.24 | | Bacteria | Bacteroidetes | Sphingobacteriia | Sphingobacteriales | Sphingobacteriaceae | Pedobacter |
| | | | | | | 0.07 | 0.07 | | 0.54 | | 0.07 | | | | | | | | | | | | | | 0.25 | | | 0.10 | | | Bacteria | Firmicutes | Negativicutes | Selenomonadales | Veillonellaceae | Anaerospora |
| | | | | | | 0.07 | | | | | | | | | 0.07 | 0.06 | 0.14 | 0.07 | | | | | | | | 0.26 | | | | | Bacteria | Bacteroidetes | Flavobacteriia | Flavobacteriales | Flavobacteriaceae | Empedobacter |
| | 0.30 | | | | 0.14 | | | | | | | | | | | | | 0.04 | | | | | | | | 0.07 | | | | 0.20 | Bacteria | Proteobacteria | Alphaproteobacteria | Rhizobiales | Rhizobiaceae | Shinella |
| | | | | | | | | | | | | | | | | 0.17 | 0.17 | 0.25 | | 0.10 | 0.03 | | | | | | | | | | Bacteria | Bacteroidetes | Cytophagia | Cytophagales | Cytophagaceae | Dyadobacter |
| | | | | | | | | | | | | | | | 0.04 | | 0.31 | | | | | | 0.05 | 0.10 | | | | | 0.03 | | Bacteria | Verrucomicrobia | Spartobacteria | Unclassified order | Unclassified family | Other |
| | | | | | | | | | | | | | | | | | 0.12 | | | | 0.03 | | 0.05 | | | 0.07 | 0.24 | | | | Bacteria | Proteobacteria | Alphaproteobacteria | Rhizobiales | Hyphomicrobiaceae | Devosia |
| | | | | 0.09 | | | 0.07 | | | | | | | | | | 0.02 | | | | 0.03 | | | 0.20 | | | | | | | Bacteria | Proteobacteria | Alphaproteobacteria | Caulobacterales | Caulobacteraceae | Brevundimonas |
| | | | | | | | | | | | | | | | 0.04 | | 0.02 | | | 0.10 | 0.03 | 0.18 | | | | | | | | | Bacteria | Actinobacteria | Thermoleophilia | Thermoleophilales | Thermoleophilaceae | Other |
| | | | | | | 0.07 | 0.07 | | | | | | | | | 0.08 | | 0.07 | | | | | | | | | | | | 0.04 | Bacteria | Firmicutes | Clostridia | Clostridiales | Clostridiaceae | Clostridium |
| | | | | | | | | | | | | | 0.03 | | | | 0.12 | | | 0.03 | | | 0.10 | | | | | | 0.03 | | Bacteria | Firmicutes | Bacilli | Bacillales | Bacillaceae | Other |
| | | | | | | | | | | | | | | | 0.07 | 0.06 | 0.10 | | | 0.03 | | | | | | 0.04 | | | | | Bacteria | Firmicutes | Bacilli | Bacillales | Listeriaceae | Other |
| | | | | | | | | | | | | | | | 0.04 | | 0.10 | | | | 0.03 | | | | | | 0.08 | | 0.03 | | Bacteria | Firmicutes | Bacilli | Bacillales | Paenibacillaceae | Cohnella |
| | | | | | 0.07 | | | | | | | | | | 0.04 | | 0.02 | | | 0.03 | | | | | | | | 0.03 | | | Bacteria | Firmicutes | Bacilli | Bacillales | Planococcaceae | Other |
| | | | | | | | | | | | | | | | | | 1.23 | 5.05 | | | | | | | | | 0.16 | | 0.43 | | Bacteria | Firmicutes | Negativicutes | Selenomonadales | Veillonellaceae | Sporomusa |
| | | | | | | | | | | | | | | | | 0.14 | 0.05 | | | 0.72 | | | | | | 0.37 | | | | | Bacteria | Proteobacteria | Alphaproteobacteria | Sphingomonadales | Sphingomonadaceae | Sphingobium |
| | | | | | | | | | | | | | | | 0.04 | 0.53 | 0.48 | | | 0.07 | | | | | | | | | | | Bacteria | Proteobacteria | Gammaproteobacteria | Xanthomonadales | Other | Other |
| | | | | | | | | | | | | | | | | | 0.43 | 0.21 | | | | | | | | | | | 0.06 | 0.04 | Bacteria | Firmicutes | Clostridia | Clostridiales | Ruminococcaceae | Ruminiclostridium |
| | | | | | | | | | | | | | | | 0.11 | 0.08 | | 0.04 | | | | | | | | | 0.08 | | | | Bacteria | Proteobacteria | Betaproteobacteria | Rhodocyclales | Rhodocyclaceae | Georgfuchsia |
| | | | | 0.17 | | | | | | | | | | | | | | 0.07 | | 0.03 | | | | | | | | | 0.03 | | Bacteria | Proteobacteria | Gammaproteobacteria | Thiotrichales | Other | Other |
| | | | 0.20 | | | | | | | | | | | | | | 0.02 | | | 0.03 | 0.03 | | | | | | | | | | Bacteria | Verrucomicrobia | Verrucomicrobiae | Verrucomicrobiales | Verrucomicrobiaceae | Other |
| | | | | | | | | | | | | | | | 0.11 | | | | | | | | | 0.10 | | 0.04 | | | 0.03 | | Bacteria | Synergistetes | Synergistia | Synergistales | Other | Other |
| | | | | | | | | | | | | | | | | | 0.05 | | 0.05 | 0.10 | | | | | | | 0.04 | | | | Bacteria | Firmicutes | Clostridia | Clostridiales | Other | Other |
| | | | | | | | | | | | | | | | 0.04 | | 0.05 | | | | 0.03 | | 0.05 | | | | | | | | Bacteria | Planctomycetes | Planctomycetia | Planctomycetales | Planctomycetaceae | Pirellula |
| | | | | | | | | | | | | | | | 0.04 | | | 0.04 | | 0.03 | | | | | | | | | 0.06 | | Bacteria | Planctomycetes | Planctomycetia | Planctomycetales | Planctomycetaceae | Gemmata |
| | | | | | | | | | | | | | | | | | | | | | | | 0.05 | | | 0.04 | | | 0.03 | 0.04 | Bacteria | Actinobacteria | Actinobacteridae | Actinomycetales | Micrococcineae | Cellulosimicrobium |
| | | | | | | | | | | | | | | | 0.04 | | | | 0.05 | 0.03 | 0.03 | | | | | | | | | | Bacteria | Chloroflexi | Chloroflexia | Chloroflexales | Other | Other |
| | | | | | | | | | | | | | | | 0.04 | | 0.02 | | | 0.03 | | | 0.05 | | | | | | | | Bacteria | Verrucomicrobia | Verrucomicrobiae | Verrucomicrobiales | Verrucomicrobiaceae | Prosthecobacter |
| | | | | | | | | | | | | | | | 23.44 | | 0.05 | 10.09 | | | | | | | | | | | | | Bacteria | Proteobacteria | Betaproteobacteria | Rhodocyclales | Rhodocyclaceae | Azospira |
| 0.28 | | 1.13 | | | | | | | | | | | | | | | | 0.14 | | | | | | | | | | | | | Bacteria | Proteobacteria | Alphaproteobacteria | Rhizobiales | Methylocystaceae | Pleomorphomonas |
| | | | | | | | | | | | | | | | | | 0.02 | 0.04 | | | | | | | | 0.26 | | | | | Bacteria | Verrucomicrobia | Opitutae | Opitutales | Opitutaceae | Opitutus |
| | | | | | | | | | | | | | | | 0.04 | | 0.19 | | | | 0.07 | | | | | | | | | | Bacteria | Bacteroidetes | Sphingobacteriia | Sphingobacteriales | Chitinophagaceae | Other |
| | | | 0.20 | | | | | | | | | | | | | | 0.02 | | | | | | | | | | 0.04 | | | | Bacteria | Bacteroidetes | Cytophagia | Cytophagales | Other | Other |
| | | | | | | | | | | | | | | | 0.04 | | | | | | 0.03 | 0.18 | | | | | | | | | Bacteria | Verrucomicrobia | Verrucomicrobiae | Verrucomicrobiales | Other | Other |
| | | | | | | | | | | | | | | | | | 0.05 | | | | | | 0.10 | 0.10 | | | | | | | Bacteria | Chloroflexi | Chloroflexia | Chloroflexales | Chloroflexineae | Chloroflexus |
| | | | | | | | | | | | | | | | | | 0.10 | | | | 0.07 | | 0.05 | | | | | | | | Bacteria | Proteobacteria | Betaproteobacteria | Burkholderiales | Comamonadaceae | Pseudorhodoferax |
| | | | | | | | | | | | | | | | | | 0.05 | 0.11 | | | | | | | | | 0.04 | | | | Bacteria | Bacteroidetes | Sphingobacteriia | Sphingobacteriales | Chitinophagaceae | Flavitalea |
| | | | | | | | | | | | 0.07 | | | | 0.04 | | | 0.07 | | | | | | | | | | | | | Bacteria | Firmicutes | Bacilli | Lactobacillales | Carnobacteriaceae | Other |
| | | | | | | | | | | | | | | | | | 0.02 | | | | 0.07 | | 0.05 | | | | | | | | Bacteria | Planctomycetes | Planctomycetia | Planctomycetales | Planctomycetaceae | Bythopirellula |
| | | | | | | | | | | | | | | | | | | 0.04 | | | | | 0.05 | | | | 0.04 | | | | Bacteria | Firmicutes | Bacilli | Bacillales | Thermoactinomycetaceae | Other |
| | | | | | | | | | | | | | 0.03 | | | 0.06 | | | | | | | | | | | | 0.03 | | | Bacteria | Proteobacteria | Alphaproteobacteria | Rhizobiales | Rhizobiaceae | Ensifer |
| | | | | | | | | | | | | | | | | | 0.02 | | | 0.03 | | | | | | | | | 0.06 | | Bacteria | Proteobacteria | Alphaproteobacteria | Rhizobiales | Rhodobiaceae | Other |
| | | | | | | | | | | | | | | | | | | | | | 0.03 | | | | | 0.04 | | 0.03 | | | Bacteria | Firmicutes | Bacilli | Bacillales | Bacillaceae | Bacillus |
| | | | | | | | | | | | | | 0.03 | | 0.04 | | | 0.04 | | | | | | | | | | | | | Bacteria | Proteobacteria | Alphaproteobacteria | Rhizobiales | Xanthobacteraceae | Labrys |
| | | | | | | | | | | | | | | | 0.04 | | | | | | 0.03 | | | | | | | | 0.03 | | Bacteria | Proteobacteria | Deltaproteobacteria | Desulfobacterales | Desulfobacteraceae | Other |
| | | | | | | | | | | | | | | | | | | | | 0.03 | | | | | | 0.04 | | | 0.03 | | Bacteria | Proteobacteria | Deltaproteobacteria | Myxococcales | Cystobacterineae | Other |
| | | | | | | | | | | | | | | | | | 0.02 | 0.04 | | 0.03 | | | | | | | | | | | Bacteria | Thermotogae | Thermotogae | Thermotogales | Other | Other |
| | | | | | | | | | | | | | | | | 0.03 | 0.02 | | | | | | | | | 0.04 | | | | | Bacteria | Planctomycetes | Planctomycetia | Planctomycetales | Planctomycetaceae | Blastopirellula |
| | | | | | | | | | | | | | 0.02 | | | 0.03 | | | | 0.03 | | | | | | | | | | | Bacteria | Proteobacteria | Alphaproteobacteria | Rhizobiales | unclassified Rhizobiales | Other |
| | | | | | | | | | | | | | | | 0.39 | | | | | | | | | | | | 2.34 | | | | Bacteria | Proteobacteria | Gammaproteobacteria | Xanthomonadales | Xanthomonadaceae | Pseudoxanthomonas |
| | | | | | | 0.46 | | | | | | | | | | 0.08 | | | | | | | | | | | | | | | Bacteria | Firmicutes | Negativicutes | Selenomonadales | Veillonellaceae | Pelosinus |
| | | | | | | | | | | | | | | | | | | | | | | | | | | 0.18 | | 0.07 | | | Bacteria | Actinobacteria | Actinobacteridae | Actinomycetales | Micrococcineae | Leifsonia |
| | | | | | | | | | | | | | | | | | | | | | | | | | | | 0.12 | | 0.06 | | Bacteria | Proteobacteria | Alphaproteobacteria | Rhizobiales | Brucellaceae | Pseudochrobactrum |
| | | | | | | | | | | | | | | | | | 0.02 | | | | | | | | | 0.15 | | | | | Bacteria | Bacteroidetes | Sphingobacteriia | Sphingobacteriales | Chitinophagaceae | Sediminibacterium |
| | | | | | | | | | | | | | | | | | 0.02 | | | | | | | | 0.13 | | | | | | Bacteria | Firmicutes | Clostridia | Clostridiales | Clostridiales incertae sedis | Other |
| | | | | | | | | | | | | | | | 0.07 | | | 0.07 | | | | | | | | | | | | | Bacteria | Verrucomicrobia | Spartobacteria | Unclassified order | Other | Other |
| | | | | | | 0.07 | | | 0.07 | | | | | | | | | | | | | | | | | | | | | | Bacteria | Cyanobacteria | Stigonematales | Unclassified order | Unclassified family | Other |
| | | | | | | | | | | | | | | | | | 0.02 | | | | | | | 0.10 | | | | | | | Bacteria | Chloroflexi | Caldilineae | Caldilineales | Other | Other |
| | | | | | 0.07 | | | | | | | | | | | | 0.05 | | | | | | | | | | | | | | Bacteria | Proteobacteria | Betaproteobacteria | Burkholderiales | Comamonadaceae | Limnohabitans |
| | 0.08 | | | | | | | | | | | | | | | | | | | | | | | | | 0.04 | | | | | Bacteria | Proteobacteria | Deltaproteobacteria | Desulfuromonadales | Other | Other |
| | | | | | | | | | | | | | | | | 0.08 | 0.02 | | | | | | | | | | | | | | Bacteria | Chloroflexi | Thermomicrobia | Sphaerobacterales | Other | Other |
| | | | | | | | | | | | | | | | | | | 0.07 | | | | | | | | | | | 0.03 | | Bacteria | Firmicutes | Clostridia | Clostridiales | Peptococcaceae | Other |
| | | | | | | | | | | | | | | | | 0.03 | | 0.07 | | | | | | | | | | | | | Bacteria | Chloroflexi | Dehalococcoidia | Unclassified order | Other | Other |
| | | | | | | | | | | | | | | | | | 0.05 | | | | | | | | | | | | 0.03 | | Bacteria | Chloroflexi | Ktedonobacteria | Ktedonobacterales | Ktedonobacteraceae | Other |
| | | | | | | | | | | | | | | | | | | | | | | | | | | 0.04 | | | | 0.04 | Bacteria | Proteobacteria | Gammaproteobacteria | Xanthomonadales | Xanthomonadaceae | Dokdonella |
| | | | | | | | | | | | | | | | | | 0.02 | | | | | | 0.05 | | | | | | | | Bacteria | Proteobacteria | Gammaproteobacteria | Enterobacteriales | Enterobacteriaceae | Kluyvera |
| | | | | | | | | | | | | | | | 0.04 | | | | | 0.03 | | | | | | | | | | | Bacteria | Firmicutes | Clostridia | Clostridiales | Syntrophomonadaceae | Other |
| | | | | | | | | | | | | | | | | | | | | | | | | | | 0.04 | | | 0.03 | | Bacteria | Proteobacteria | Gammaproteobacteria | Chromatiales | Other | Other |
| | | | | | | | | | | | | | | | | | | | | 0.03 | | | | | | | | | 0.03 | | Bacteria | Chloroflexi | Caldilineae | Caldilineales | Caldilineaceae | Other |
| | | | | | | | | | | | | | | | | | 0.02 | | | | | | | | | 0.04 | | | | | Bacteria | Proteobacteria | Alphaproteobacteria | Rhizobiales | Xanthobacteraceae | Pseudolabrys |
| | | | | | | | | | | | | | | | | 0.03 | | | | 0.03 | | | | | | | | | | | Bacteria | Bacteroidetes | Sphingobacteriia | Sphingobacteriales | Chitinophagaceae | Segetibacter |
| | | | | | | | | | | | | | | | | 0.03 | | | | 0.03 | | | | | | | | | | | Bacteria | Verrucomicrobia | Verrucomicrobiae | Verrucomicrobiales | Verrucomicrobiaceae | Verrucomicrobium |
| | | | | | | | | | | | | | | | 0.04 | | 0.02 | | | | | | | | | | | | | | Bacteria | Proteobacteria | Deltaproteobacteria | Desulfobacterales | Other | Other |
| | | | | | | | | | | | | | | | | | 0.02 | | | 0.03 | | | | | | | | | | | Bacteria | Chloroflexi | Thermomicrobia | Sphaerobacterales | Sphaerobacteraceae | Other |
| | | | | | | | | | | | | | | | | | | | | | | | | | | 2.11 | | | | | Bacteria | Proteobacteria | Gammaproteobacteria | Enterobacteriales | Enterobacteriaceae | Rosenbergiella |
| | | | | | | | | | | | | | | | | | | | | | | | | | | | 0.97 | | | | Bacteria | Proteobacteria | Alphaproteobacteria | Caulobacterales | Caulobacteraceae | Asticcacaulis |
| | | | | | | | | | | | | | 0.96 | | | | | | | | | | | | | | | | | | Bacteria | Proteobacteria | Gammaproteobacteria | Enterobacteriales | Enterobacteriaceae | Pantoea |
| | | | | | | | | | 0.68 | | | | | | | | | | | | | | | | | | | | | | Bacteria | Bacteroidetes | Sphingobacteriia | Sphingobacteriales | Chitinophagaceae | Filimonas |
| | | | | | | | | | | | | | | | | | | | | 0.46 | | | | | | | | | | | Bacteria | Verrucomicrobia | Verrucomicrobiae | Verrucomicrobiales | Verrucomicrobiaceae | Roseimicrobium |
| | | | | | | | | | | | | | | | | | 0.39 | | | | | | | | | | | | | | Bacteria | Proteobacteria | Gammaproteobacteria | Unclassified order | Unclassified family | Thiohalobacter |
| | | | | | | | | | | | | | 0.36 | | | | | | | | | | | | | | | | | | Bacteria | Proteobacteria | Gammaproteobacteria | Enterobacteriales | Enterobacteriaceae | Kosakonia |
| | | 0.35 | | | | | | | | | | | | | | | | | | | | | | | | | | | | | Bacteria | Firmicutes | Negativicutes | Selenomonadales | Veillonellaceae | Dendrosporobacter |
| | | | | | | | | | | | | | | | | | 0.34 | | | | | | | | | | | | | | Bacteria | Verrucomicrobia | Verrucomicrobiae | Verrucomicrobiales | Verrucomicrobiaceae | Luteolibacter |
| | | | | | | 0.33 | | | | | | | | | | | | | | | | | | | | | | | | | Bacteria | Proteobacteria | Gammaproteobacteria | Enterobacteriales | Enterobacteriaceae | Pseudocitrobacter |
| | | | | | | | | | | | | | | 0.25 | | | | | | | | | | | | | | | | | Bacteria | Bacteroidetes | Bacteroidia | Bacteroidales | Bacteroidaceae | Bacteroides |
| | | | | | | | | | | | | | | | | | | | | | | | | | | | 0.24 | | | | Bacteria | Bacteroidetes | Sphingobacteriia | Sphingobacteriales | Chitinophagaceae | Niabella |
| | | | | | | | | | 0.20 | | | | | | | | | | | | | | | | | | | | | | Bacteria | Proteobacteria | Gammaproteobacteria | Enterobacteriales | Enterobacteriaceae | Providencia |
| | 0.15 | | | | | | | | | | | | | | | | | | | | | | | | | | | | | | Bacteria | Actinobacteria | Actinobacteridae | Actinomycetales | Micrococcineae | Promicromonospora |
| | | | | | | | | | | | | | 0.14 | | | | | | | | | | | | | | | | | | Bacteria | Proteobacteria | Betaproteobacteria | Burkholderiales | Oxalobacteraceae | Oxalicibacterium |
| | | | | | | | | | | | | | | | | | | | | | | | | | 0.13 | | | | | | Bacteria | Proteobacteria | Gammaproteobacteria | Xanthomonadales | Xanthomonadaceae | Other |
| | | | | | | | | | | | | | | | | | | | | | | | | | | | | | 0.12 | | Bacteria | Firmicutes | Clostridia | Clostridiales | Gracilibacteraceae | Gracilibacter |
| | | | | | | | | | | | | | | | | | | | | | | | | | | | | 0.10 | | | Bacteria | Bacteroidetes | Flavobacteriia | Flavobacteriales | Flavobacteriaceae | Elizabethkingia |
| | | | | | | | | | | | | | | | | | | | | | | | 0.10 | | | | | | | | Bacteria | Proteobacteria | Betaproteobacteria | Methylophilales | Methylophilaceae | Methylovorus |
| | | | | | | | | 0.09 | | | | | | | | | | | | | | | | | | | | | | | Bacteria | Actinobacteria | Actinobacteridae | Actinomycetales | Frankineae | Modestobacter |
| | | | | | | | | 0.09 | | | | | | | | | | | | | | | | | | | | | | | Bacteria | Firmicutes | Clostridia | Clostridiales | Clostridiales incertae sedis | Anaerovorax |
| | | | | 0.09 | | | | | | | | | | | | | | | | | | | | | | | | | | | Bacteria | Actinobacteria | Actinobacteridae | Actinomycetales | Micrococcineae | Sanguibacter |
| | | | | | | | | | | 0.08 | | | | | | | | | | | | | | | | | | | | | Bacteria | Proteobacteria | Gammaproteobacteria | Aeromonadales | Aeromonadaceae | Aeromonas |
| | 0.08 | | | | | | | | | | | | | | | | | | | | | | | | | | | | | | Bacteria | Proteobacteria | Betaproteobacteria | Burkholderiales | unclassified Burkholderiales | Mitsuaria |
| | | | | | | | | | | | | | | | | | | | | | | | | | | 0.07 | | | | | Bacteria | Proteobacteria | Alphaproteobacteria | Rhodospirillales | Acetobacteraceae | Roseomonas |
| | | | | | | | | | 0.07 | | | | | | | | | | | | | | | | | | | | | | Bacteria | Firmicutes | Clostridia | Clostridiales | Clostridiales incertae sedis | Thermaerobacter |
| | | | | | | | | | 0.07 | | | | | | | | | | | | | | | | | | | | | | Bacteria | Planctomycetes | Planctomycetia | Planctomycetales | Planctomycetaceae | Aquisphaera |
| | | | | | 0.07 | | | | | | | | | | | | | | | | | | | | | | | | | | Bacteria | Proteobacteria | Deltaproteobacteria | Desulfovibrionales | Other | Other |
| | | | | | | 0.07 | | | | | | | | | | | | | | | | | | | | | | | | | Bacteria | Proteobacteria | Alphaproteobacteria | Rhizobiales | Aurantimonadaceae | Martelella |
| | | | | | | 0.07 | | | | | | | | | | | | | | | | | | | | | | | | | Bacteria | Proteobacteria | Gammaproteobacteria | Pseudomonadales | Moraxellaceae | Alkanindiges |
| | | | | | | | | | | | | | | | | | 0.05 | | | | | | | | | | | | | | Bacteria | Actinobacteria | Rubrobacteridae | Gaiellales | Gaiellaceae | Gaiella |
| | | | | | | | | | | | | | | | | | 0.05 | | | | | | | | | | | | | | Bacteria | Armatimonadetes | Armatimonadia | Armatimonadales | Armatimonadaceae | Armatimonas |
| | | | | | | | | | | | | | | | | | | | | | | | | | | | 0.04 | | | | Bacteria | Actinobacteria | Acidimicrobidae | Acidimicrobiales | Other | Other |
| | | | | | | | | | | | | | | | | | | | | | | | | | | | 0.04 | | | | Bacteria | Actinobacteria | Actinobacteridae | Actinomycetales | Micrococcineae | Arthrobacter |
| | | | | | | | | | | | | | | | | | | | | | | | | | | | 0.04 | | | | Bacteria | Actinobacteria | Rubrobacteridae | Solirubrobacterales | Solirubrobacteraceae | Solirubrobacter |
| | | | | | | | | | | | | | | | | | | | | | | | | | | | 0.04 | | | | Bacteria | Bacteroidetes | Sphingobacteriia | Sphingobacteriales | Chitinophagaceae | Niastella |
| | | | | | | | | | | | | | | | | | | | | | | | | | | | 0.04 | | | | Bacteria | Bacteroidetes | Sphingobacteriia | Sphingobacteriales | Chitinophagaceae | Terrimonas |
| | | | | | | | | | | | | | | | | | | | | | | | | | | | 0.04 | | | | Bacteria | Proteobacteria | Alphaproteobacteria | Rhizobiales | Brucellaceae | Brucella |
| | | | | | | | | | | | | | | | | | | | | | | | | | | | | | | 0.04 | Bacteria | Proteobacteria | Alphaproteobacteria | Rhizobiales | unclassified Rhizobiales | Vasilyevaea |
| | | | | | | | | | | | | 0.04 | | | | | | | | | | | | | | | | | | | Bacteria | Chloroflexi | Thermomicrobia | Thermomicrobiales | Other | Other |
| | | | | | | | | | | | | | | | | | | | | | | | | | | 0.04 | | | | | Bacteria | Bacteroidetes | Cytophagia | Cytophagales | Cytophagaceae | Adhaeribacter |
| | | | | | | | | | | | | | | | | | | | | | | | | | | 0.04 | | | | | Bacteria | Planctomycetes | Planctomycetia | Planctomycetales | Planctomycetaceae | Schlesneria |
| | | | | | | | | | | | | | | | | | | | | | | | | | | 0.04 | | | | | Bacteria | Proteobacteria | Alphaproteobacteria | Rhodospirillales | Acetobacteraceae | Acidiphilium |
| | | | | | | | | | | | | | | | | | | | | | | | | | | 0.04 | | | | | Bacteria | Proteobacteria | Alphaproteobacteria | Sphingomonadales | Sphingomonadaceae | Other |
| | | | | | | | | | | | | | | | | | | 0.04 | | | | | | | | | | | | | Bacteria | Acidobacteria | Acidobacteriia | Acidobacteriales | Acidobacteriaceae | Candidatus Koribacter |
| | | | | | | | | | | | | | | | | | | 0.04 | | | | | | | | | | | | | Bacteria | Proteobacteria | Alphaproteobacteria | Rhizobiales | Hyphomicrobiaceae | Rhodoplanes |
| | | | | | | | | | | | | | | | | | | 0.04 | | | | | | | | | | | | | Bacteria | Proteobacteria | Betaproteobacteria | Burkholderiales | Comamonadaceae | Ramlibacter |
| | | | | | | | | | | | | | | | | | | 0.04 | | | | | | | | | | | | | Bacteria | Proteobacteria | Betaproteobacteria | Burkholderiales | unclassified Burkholderiales | Thiobacter |
| | | | | | | | | | | | | | | | | | | 0.04 | | | | | | | | | | | | | Bacteria | Proteobacteria | Gammaproteobacteria | Enterobacteriales | Enterobacteriaceae | Raoultella |
| | | | | | | | | | | | | | | | 0.04 | | | | | | | | | | | | | | | | Bacteria | Actinobacteria | Actinobacteridae | Actinomycetales | Kineosporiineae | Kineosporia |
| | | | | | | | | | | | | | | | 0.04 | | | | | | | | | | | | | | | | Bacteria | Chloroflexi | Anaerolineae | Anaerolineales | Other | Other |
| | | | | | | | | | | | | | | | 0.04 | | | | | | | | | | | | | | | | Bacteria | Proteobacteria | Alphaproteobacteria | Rhizobiales | unclassified Rhizobiales | Nordella |
| | | | | | | | | | | | | | | | 0.04 | | | | | | | | | | | | | | | | Bacteria | Proteobacteria | Alphaproteobacteria | Rhodothalassiales | Rhodothalassiaceae | Other |
| | | | | | | | | | | | | | | | | | | | | | 0.03 | | | | | | | | | | Bacteria | Gemmatimonadetes | Gemmatimonadetes | Gemmatimonadales | Gemmatimonadaceae | Gemmatimonas |
| | | | | | | | | | | | | | | | | | | | | | 0.03 | | | | | | | | | | Bacteria | Spirochaetes | Spirochaetia | Spirochaetales | Other | Other |
| | | | | | | | | | | | | | | | | | | | | | | | | | | | | 0.03 | | | Bacteria | Proteobacteria | Gammaproteobacteria | unclassified Gammaproteob | Unclassified family | Acidibacter |
| | | | | | | | | | | | | | | | | | | | | 0.03 | | | | | | | | | | | Bacteria | Acidobacteria | Solibacteres | Solibacterales | Solibacteraceae | Candidatus Solibacter |
| | | | | | | | | | | | | | | | | | | | | 0.03 | | | | | | | | | | | Bacteria | Aquificae | Aquificae | Aquificales | Other | Other |
| | | | | | | | | | | | | | | | | | | | | 0.03 | | | | | | | | | | | Bacteria | Chloroflexi | Thermoflexia | Thermoflexales | Other | Other |
| | | | | | | | | | | | | | | | | | | | | 0.03 | | | | | | | | | | | Bacteria | Dictyoglomi | Dictyoglomia | Dictyoglomales | Other | Other |
| | | | | | | | | | | | | | | | | | | | | 0.03 | | | | | | | | | | | Bacteria | Planctomycetes | Phycisphaerae | Phycisphaerales | Phycisphaeraceae | Other |
| | | | | | | | | | | | | | | | | | | | | 0.03 | | | | | | | | | | | Bacteria | Proteobacteria | Alphaproteobacteria | Rhizobiales | Other | Other |
| | | | | | | | | | | | | | | | | | | | | 0.03 | | | | | | | | | | | Bacteria | Proteobacteria | Alphaproteobacteria | Rhizobiales | Rhizobiaceae | Kaistia |
| | | | | | | | | | | | | | | | | | | | | 0.03 | | | | | | | | | | | Bacteria | Proteobacteria | Alphaproteobacteria | Sneathiellales | Other | Other |
| | | | | | | | | | | | | | | | | | | | | 0.03 | | | | | | | | | | | Bacteria | Proteobacteria | Gammaproteobacteria | Enterobacteriales | Enterobacteriaceae | Cronobacter |
| | | | | | | | | | | | | | | | | | | | | 0.03 | | | | | | | | | | | Bacteria | Proteobacteria | Gammaproteobacteria | Pseudomonadales | Other | Other |
| | | | | | | | | | | | | | | | | | | | | | | | | | | | | | 0.03 | | Bacteria | Proteobacteria | Gammaproteobacteria | Enterobacteriales | Enterobacteriaceae | Phaseolibacter |
| | | | | | | | | | | | | | | | | 0.03 | | | | | | | | | | | | | | | Bacteria | Proteobacteria | Gammaproteobacteria | Oceanospirillales | Other | Other |
| | | | | | | | | | | | | | | | | | 0.02 | | | | | | | | | | | | | | Archaea | Euryarchaeota | Halobacteria | Halobacteriales | Halobacteriaceae | Halonotius |
| | | | | | | | | | | | | | | | | | 0.02 | | | | | | | | | | | | | | Bacteria | Actinobacteria | Actinobacteridae | Actinomycetales | Other | Other |
| | | | | | | | | | | | | | | | | | 0.02 | | | | | | | | | | | | | | Bacteria | Actinobacteria | Rubrobacteridae | Solirubrobacterales | Conexibacteraceae | Other |
| | | | | | | | | | | | | | | | | | 0.02 | | | | | | | | | | | | | | Bacteria | Bacteroidetes | Sphingobacteriia | Sphingobacteriales | Chitinophagaceae | Flavisolibacter |
| | | | | | | | | | | | | | | | | | 0.02 | | | | | | | | | | | | | | Bacteria | Deinococcus-Thermus | Deinococci | Deinococcales | Deinococcaceae | Deinococcus |
| | | | | | | | | | | | | | | | | | 0.02 | | | | | | | | | | | | | | Bacteria | Firmicutes | Bacilli | Lactobacillales | Enterococcaceae | Other |
| | | | | | | | | | | | | | | | | | 0.02 | | | | | | | | | | | | | | Bacteria | Firmicutes | Clostridia | Thermoanaerobacterales | Thermoanaerobacterales Fa | Other |
| | | | | | | | | | | | | | | | | | 0.02 | | | | | | | | | | | | | | Bacteria | Firmicutes | Thermolithobacteria | Thermolithobacterales | Thermolithobacteraceae | Other |
| | | | | | | | | | | | | | | | | | 0.02 | | | | | | | | | | | | | | Bacteria | Firmicutes | unclassified Firmicutes sen | Unclassified order | Other | Other |
| | | | | | | | | | | | | | | | | | 0.02 | | | | | | | | | | | | | | Bacteria | Proteobacteria | Alphaproteobacteria | Rhizobiales | Beijerinckiaceae | Methylorosula |
| | | | | | | | | | | | | | | | | | 0.02 | | | | | | | | | | | | | | Bacteria | Proteobacteria | Betaproteobacteria | Rhodocyclales | Rhodocyclaceae | Azoarcus |
| | | | | | | | | | | | | | | | | | 0.02 | | | | | | | | | | | | | | Bacteria | Proteobacteria | Deltaproteobacteria | Desulfuromonadales | Pelobacteraceae | Other |
| | | | | | | | | | | | | | | | | | 0.02 | | | | | | | | | | | | | | Bacteria | Proteobacteria | Deltaproteobacteria | Syntrophobacterales | Other | Other |
| | | | | | | | | | | | | | | | | | 0.02 | | | | | | | | | | | | | | Bacteria | Proteobacteria | Gammaproteobacteria | Enterobacteriales | Enterobacteriaceae | Escherichia |
| | | | | | | | | | | | | | | | | | 0.02 | | | | | | | | | | | | | | Bacteria | Proteobacteria | Gammaproteobacteria | Pseudomonadales | Moraxellaceae | Other |
| | | | | | | | | | | | | | | | | | 0.02 | | | | | | | | | | | | | | Bacteria | unclassified Bacteria | candidate division NC10 | Unclassified order | Unclassified family | Other |

**Supplementary Figure S5: Abundance and distribution of all genera found in soybean root-associated samples by pyrotags.** All root-associated sequences were taxonomically assigned by BLASTN against NCBI 16S rRNA and were classified to genus (>90% similarity to best-hit), family or order (depending on the case for hits <90% similarity to best-hit). Sequences were grouped by sample, site and classification and relative abundances were calculated according to total number of sequences per sample. Color scales indicate the relative abundances, with a gradual transition between green (high abundant) to red (low abundant).

## Wheat root-associated bacteria observed by pyrotags

| Site | | | | | | | | | | | | | | | | | | | | | | | | Taxonomy | | | | | |
|---|---|---|---|---|---|---|---|---|---|---|---|---|---|---|---|---|---|---|---|---|---|---|---|---|---|---|---|---|---|
| Balcarce | | | | | | Corral de Bustos | | | | | | Daireaux | | | | | | Villa Saboya | | | | | | Domain | Phylum | Class | Order | Family | Genus |
| 0.33 | 0.18 | 0.43 | 0.36 | 1.34 | | 0.30 | 0.20 | 0.30 | 1.40 | 0.72 | 0.24 | 18.26 | 0.66 | 0.27 | 0.07 | 0.36 | 0.14 | 10.56 | 23.30 | 0.60 | 13.79 | 0.22 | 6.48 | Bacteria | Verrucomicrobia | Spartobacteria | Unclassified order | Unclassified family | Chthoniobacter |
| | 0.96 | 4.07 | 68.67 | 0.89 | 85.41 | | 0.05 | | 6.76 | 0.36 | 27.02 | 21.90 | 80.73 | 96.78 | 86.96 | 58.20 | 0.07 | 10.64 | 2.06 | 22.93 | 1.07 | 87.55 | 2.66 | Bacteria | Proteobacteria | Gammaproteobacteria | Pseudomonadales | Pseudomonadaceae | Pseudomonas |
| 0.33 | 0.11 | 0.43 | 0.12 | 0.15 | 0.16 | | | 0.10 | 1.26 | 0.24 | | 0.19 | 0.36 | 0.18 | 0.04 | 0.84 | 0.43 | 6.68 | 7.28 | 0.45 | 17.24 | 0.15 | 6.03 | Bacteria | Planctomycetes | Planctomycetia | Planctomycetales | Other | Other |
| 0.11 | 0.32 | | | 0.97 | | 0.30 | 0.25 | 0.45 | 1.55 | 0.48 | 0.24 | 0.06 | 0.24 | | | | 0.22 | 6.09 | 5.70 | | 2.79 | 0.04 | 6.58 | Bacteria | Acidobacteria | unclassified Acidobacteria | Acidobacteria subdivision 4 | Unclassified family | Blastocatella |
| | 0.07 | 0.21 | | 0.15 | | | 0.05 | 0.10 | 0.81 | 0.12 | 0.16 | | | 0.59 | 4.73 | | 0.29 | 7.19 | 4.13 | 62.75 | 0.90 | | 5.02 | Bacteria | Firmicutes | Bacilli | Bacillales | Bacillaceae | Bacillus |
| 52.86 | 0.02 | 0.64 | 2.28 | | 2.46 | | | | 0.11 | | | 0.68 | 0.30 | | | 9.02 | 74.52 | 1.83 | 0.12 | | 0.82 | 0.04 | 0.10 | Bacteria | Planctomycetes | Planctomycetia | Planctomycetales | Planctomycetaceae | Singulisphaera |
| 0.11 | 7.37 | 8.57 | 0.84 | | 0.66 | 0.15 | | | | | 0.97 | 0.43 | | 1.54 | | 1.47 | | 0.29 | 0.24 | 0.60 | 0.08 | | 0.35 | Bacteria | Proteobacteria | Betaproteobacteria | Burkholderiales | Burkholderiaceae | Burkholderia |
| 1.98 | 41.11 | 25.27 | 1.68 | | 7.70 | | | | 1.07 | | 6.13 | 1.11 | 6.68 | | 0.11 | 2.27 | | 0.88 | | 0.30 | | | 0.05 | Bacteria | Proteobacteria | Alphaproteobacteria | Rhizobiales | Rhizobiaceae | Rhizobium |
| 0.22 | | 0.86 | 0.12 | 0.22 | | | | 0.15 | 0.41 | | 0.16 | 0.19 | | 0.05 | | | | | 8.74 | 1.06 | 8.37 | | 5.27 | Bacteria | Acidobacteria | unclassified Acidobacteria | Acidobacteria subdivision 4 | Unclassified family | Other |
| | 0.27 | 0.21 | 5.40 | | | | 0.05 | | 1.00 | | | 0.49 | 0.06 | 0.05 | | 0.04 | | 0.44 | 3.64 | | 5.50 | | 1.56 | Bacteria | Planctomycetes | Planctomycetia | Planctomycetales | Planctomycetaceae | Other |
| 2.31 | | | 0.60 | 1.19 | | | | | 0.04 | | | 0.06 | | | 0.11 | | 1.79 | 5.72 | 0.12 | 1.36 | | 8.71 | 4.67 | Bacteria | Proteobacteria | Gammaproteobacteria | Enterobacteriales | Enterobacteriaceae | Klebsiella |
| | 0.02 | | | | | | | 0.10 | 2.18 | | 0.40 | 0.74 | 0.30 | 0.05 | | 0.18 | | 1.61 | 2.43 | | | 0.11 | 0.15 | Bacteria | Planctomycetes | Phycisphaerae | Phycisphaerales | Other | Other |
| 8.68 | 1.00 | 9.42 | | | 1.48 | 5.51 | 0.50 | | | | 0.08 | 0.19 | 7.65 | | | 0.44 | 8.40 | | | | | | | Bacteria | Actinobacteria | Actinobacteridae | Actinomycetales | Streptosporangiaceae | Microbispora |
| | 1.46 | | | | | | 41.15 | | | | 0.56 | 0.12 | 1.26 | 0.41 | 0.32 | 0.27 | 0.07 | | | | | | | Bacteria | Firmicutes | Bacilli | Bacillales | Paenibacillaceae | Paenibacillus |
| 0.11 | 0.09 | 0.21 | | | | | | | | | | 0.37 | | | | 0.09 | | 0.07 | 2.79 | | 1.81 | | 3.77 | Bacteria | Firmicutes | Clostridia | Clostridiales | Other | Other |
| 0.11 | | 0.21 | | 0.15 | | 0.15 | | 0.05 | 0.33 | | | | | | | 0.09 | | | 2.43 | | | 0.19 | | Bacteria | Proteobacteria | Alphaproteobacteria | Rhodospirillales | Rhodospirillaceae | Other |
| 0.22 | | | 0.36 | | | | | 0.10 | | | | 0.12 | | | | | | | 1.70 | 0.45 | 4.35 | | 3.87 | Bacteria | Proteobacteria | Deltaproteobacteria | Syntrophobacterales | Syntrophaceae | Other |
| 0.44 | | | | 0.30 | | | | | 0.07 | | | 6.05 | | 0.09 | | | | 0.22 | 0.36 | 0.45 | | | | Bacteria | Firmicutes | Bacilli | Bacillales | Other | Other |
| 15.05 | | 38.54 | 4.56 | | | | | | 78.84 | | 14.44 | | | | | | | 0.44 | | | | | 0.05 | Bacteria | Proteobacteria | Alphaproteobacteria | Sphingomonadales | Sphingomonadaceae | Sphingomonas |
| 1.98 | 24.25 | | 0.84 | | | | | | | | | | | | 6.65 | | | 0.07 | | 0.15 | 0.08 | | | Bacteria | Proteobacteria | Alphaproteobacteria | Caulobacterales | Caulobacteraceae | Caulobacter |
| | 0.07 | | 0.12 | 0.37 | | | | | | | | | | | | | | 2.64 | 3.16 | | | 0.04 | 0.15 | Bacteria | Chloroflexi | Thermomicrobia | Sphaerobacterales | Other | Other |
| | | | | | | | | | | | | | 0.06 | | | | 0.22 | 0.44 | 1.09 | | 1.15 | 0.07 | 3.01 | Bacteria | Chloroflexi | Thermomicrobia | Sphaerobacterales | Sphaerobacteraceae | Other |
| 0.77 | 1.41 | 2.78 | 0.36 | | | | | | | | | | | | | 0.18 | | 0.37 | | | 0.16 | | | Bacteria | Proteobacteria | Alphaproteobacteria | Rhizobiales | Bradyrhizobiaceae | Bradyrhizobium |
| 0.22 | | | | 0.30 | | | | | | | 0.24 | | | | 0.14 | 0.04 | | | | 0.15 | | | 4.17 | Bacteria | Chloroflexi | Caldilineae | Caldilineales | Other | Other |
| | 0.57 | 0.64 | | | | | | | | | | | 0.24 | | | | 0.29 | 0.81 | | 0.45 | | | 0.10 | Bacteria | Proteobacteria | Gammaproteobacteria | Thiotrichales | Other | Other |
| | 0.16 | | | | | 0.45 | | | 0.33 | | | 0.12 | 0.06 | | | | | | 0.61 | 1.36 | | | | Archaea | Thaumarchaeota | Nitrososphaerales | Nitrososphaerales | Nitrososphaeraceae | Nitrososphaera |
| | 0.16 | | | 0.52 | | 6.41 | 0.81 | | | | | | | | | | 7.61 | 0.22 | | | | | | Bacteria | Actinobacteria | Actinobacteridae | Actinomycetales | Thermomonosporaceae | Actinoallomurus |
| 5.93 | 2.56 | | 1.44 | | | | | | | | | 0.93 | | | | | | 0.51 | 0.12 | | | | | Bacteria | Proteobacteria | Alphaproteobacteria | Rhizobiales | Phyllobacteriaceae | Mesorhizobium |
| 2.42 | 2.87 | 0.86 | 0.12 | | | | | | | | | | | | | 0.13 | | | | | | | 0.05 | Bacteria | Proteobacteria | Alphaproteobacteria | Rhodospirillales | Rhodospirillaceae | Dongia |
| | 0.05 | | | 0.07 | | | | | | | | 0.19 | | | | | 0.14 | | | | 3.78 | | 0.10 | Bacteria | Verrucomicrobia | Verrucomicrobiae | Verrucomicrobiales | Other | Other |
| | 0.02 | 0.86 | 0.24 | | 0.49 | | | | | | | | | | | 0.04 | 0.65 | | | | | | | Bacteria | Actinobacteria | Rubrobacteridae | Solirubrobacterales | Solirubrobacteraceae | Solirubrobacter |
| 0.22 | | | 0.48 | | 0.82 | | | | | | | | 0.06 | | | 0.67 | | | | | | | 0.05 | Bacteria | Actinobacteria | Rubrobacteridae | Solirubrobacterales | Conexibacteraceae | Conexibacter |
| | | 0.43 | | 0.15 | | | | | | | | | | | | 0.09 | | 0.07 | | | | 0.11 | 0.05 | Bacteria | Verrucomicrobia | Spartobacteria | Unclassified order | Unclassified family | Other |
| 0.11 | 0.14 | 0.21 | | | | | | | 0.07 | 0.24 | 0.08 | | | | | | | | | | | | | Bacteria | Chloroflexi | Chloroflexia | Chloroflexales | Other | Other |
| | | | | | | | | | 0.33 | 97.35 | 0.08 | | | | | | | 0.15 | | | 0.08 | | | Bacteria | Proteobacteria | Betaproteobacteria | Burkholderiales | Burkholderiaceae | Ralstonia |
| | | | | 84.04 | 0.16 | | | | | | | | | | | | | 1.61 | 1.94 | | | | 2.11 | Bacteria | Actinobacteria | Actinobacteridae | Actinomycetales | Streptomycetaceae | Streptomyces |
| | 0.07 | | | 0.07 | | | 56.38 | | | | 7.66 | | | | | 0.04 | | | | | | | | Bacteria | Firmicutes | Negativicutes | Selenomonadales | Other | Other |
| | | | | 0.22 | | 0.30 | | | | | | 25.85 | | | | 0.04 | | | 0.73 | | | | | Bacteria | Chloroflexi | Ktedonobacteria | Ktedonobacterales | Ktedonobacteraceae | Other |
| | | | | | 0.16 | | | | | | | 14.31 | | | | | | 0.37 | | 0.15 | | 0.04 | | Bacteria | Proteobacteria | Gammaproteobacteria | Xanthomonadales | Other | Other |
| | 2.65 | | 6.36 | 0.52 | | | | | | | | 2.71 | | | | 0.04 | | | | | | | | Bacteria | Planctomycetes | Planctomycetia | Planctomycetales | Planctomycetaceae | Aquisphaera |
| | | | | | | | | | | | 0.65 | | | | | | | 8.14 | 0.49 | 0.90 | | | 0.50 | Bacteria | Proteobacteria | Betaproteobacteria | Burkholderiales | Oxalobacteraceae | Duganella |
| | | | | | | | | | 0.55 | | | | | | | 0.04 | | | | | 8.13 | 0.04 | 0.10 | Bacteria | Verrucomicrobia | Verrucomicrobiae | Verrucomicrobiales | Verrucomicrobiaceae | Prosthecobacter |
| | | | | | | | | | | 0.12 | | | | | 0.04 | | | 1.39 | | | 0.08 | | 4.22 | Bacteria | Proteobacteria | Betaproteobacteria | Burkholderiales | Comamonadaceae | Variovorax |
| | | | | | | | | | | | | 0.12 | 0.06 | | | | | 1.61 | 1.58 | | | | 0.55 | Bacteria | Cyanobacteria | Oscillatoriophycideae | Oscillatoriales | Unclassified family | Other |
| 0.33 | 1.71 | 1.28 | | | | | | | | | | 0.12 | | | | 0.13 | | | | | | | | Bacteria | Proteobacteria | Alphaproteobacteria | Rhizobiales | Bradyrhizobiaceae | Tardiphaga |
| | | | | | | | | | | | 0.08 | | | | | 0.04 | | 0.37 | 0.36 | | | | 0.45 | Bacteria | Actinobacteria | Actinobacteridae | Actinomycetales | Other | Other |
| | | | 0.72 | | | | | | | | 30.00 | 0.25 | | | | | | 0.29 | | | | | | Bacteria | Proteobacteria | Gammaproteobacteria | Xanthomonadales | Xanthomonadaceae | Dyella |
| | | | 1.44 | 0.22 | | | | | | | | | | | | | | 0.07 | | | | | 23.86 | Bacteria | Proteobacteria | Gammaproteobacteria | Enterobacteriales | Enterobacteriaceae | Serratia |
| | | 1.93 | | | | | | | | | 2.66 | | | | | | | 1.32 | | | | 2.30 | | Bacteria | Proteobacteria | Betaproteobacteria | Burkholderiales | Oxalobacteraceae | Herbaspirillum |
| | | | 0.12 | | | | | | | | | | | | | | | 4.11 | 0.24 | | | | 0.05 | Bacteria | Proteobacteria | Gammaproteobacteria | Enterobacteriales | Enterobacteriaceae | Pantoea |
| | | | | | | | | | | | | | | | | 0.04 | 0.14 | | 2.31 | | | | 1.56 | Bacteria | Armatimonadetes | Chthonomonadetes | Chthonomonadales | Chthonomonadaceae | Other |
| | | | | | | | | | | | | 0.06 | | | | | | 0.07 | 1.46 | | | | 0.10 | Bacteria | Planctomycetes | Planctomycetia | Planctomycetales | Planctomycetaceae | Bythopirellula |
| | | | | | | | | 0.05 | | | | | | | 0.07 | | | 1.03 | | | | | 0.20 | Bacteria | Proteobacteria | Betaproteobacteria | Burkholderiales | Oxalobacteraceae | Massilia |
| | | | | | | 0.15 | | | | | | 0.12 | | | | | | 0.95 | | | 0.08 | | | Bacteria | Proteobacteria | Alphaproteobacteria | Sphingomonadales | Sphingomonadaceae | Novosphingobium |
| | | | 0.96 | | | | | | | | | 0.06 | 0.12 | | | 0.04 | | | | | | | | Bacteria | Proteobacteria | Alphaproteobacteria | Rhizobiales | Xanthobacteraceae | Pseudolabrys |
| | 0.05 | | | | | | | | 0.04 | | | | | | | | | 0.07 | | 0.60 | | | | Bacteria | Bacteroidetes | Flavobacteriia | Flavobacteriales | Flavobacteriaceae | Chryseobacterium |
| | | | 0.24 | | | | 0.05 | | | | | | | | | | | | | 0.15 | 0.25 | | | Bacteria | Armatimonadetes | Armatimonadia | Armatimonadales | Armatimonadaceae | Armatimonas |
| | 0.05 | | | | | | | | | | | | | | | | | 0.07 | | 0.30 | 0.08 | | | Bacteria | Proteobacteria | Betaproteobacteria | Burkholderiales | Comamonadaceae | Delftia |
| | 0.05 | | | 0.07 | | | | | | | | | | | | 0.09 | 0.22 | | | | | | | Bacteria | Actinobacteria | Actinobacteridae | Actinomycetales | Mycobacteriaceae | Mycobacterium |
| 0.88 | | | | | | 84.35 | | | | | 4.27 | | | | | | | | | | | | | Bacteria | Bacteroidetes | Sphingobacteriia | Sphingobacteriales | Sphingobacteriaceae | Mucilaginibacter |
| | | | | | | | | | | | | | | | | | | 5.72 | | | 5.50 | 0.07 | | Bacteria | Chloroflexi | Caldilineae | Caldilineales | Caldilineaceae | Other |
| | 7.03 | | | | | | | | | | | 0.68 | | | | 0.09 | | | | | | | | Bacteria | Actinobacteria | Actinobacteridae | Actinomycetales | Micromonosporaceae | Dactylosporangium |
| | | 0.21 | | | | | | | | | | | | | | | | | | | 3.45 | | 0.05 | Bacteria | Chloroflexi | Ktedonobacteria | Ktedonobacterales | Other | Other |
| | 0.02 | | | | 0.33 | | | | | | | | | | | | | | 2.91 | | | | | Bacteria | Proteobacteria | Deltaproteobacteria | Myxococcales | Cystobacteraceae | Other |
| | | | 0.12 | | | | | | | | | 0.19 | | | | 1.95 | | | | | | | | Bacteria | Gemmatimonadetes | Gemmatimonadetes | Gemmatimonadales | Gemmatimonadaceae | Other |
| | | | | | | | | | | | | | 0.18 | | 0.04 | | | | | | | | 1.26 | Bacteria | Firmicutes | Bacilli | Bacillales | Listeriaceae | Other |
| | | | | 0.07 | | | | | | | | | | | | | | 0.59 | | | | | 0.65 | Bacteria | Bacteroidetes | Flavobacteriia | Flavobacteriales | Flavobacteriaceae | Flavobacterium |
| | | | | | | | | | 0.33 | | | | | | | | | | 0.85 | | | 0.04 | | Bacteria | Verrucomicrobia | Spartobacteria | Unclassified order | Other | Other |
| | | | | | | | | | | | | | | | | 0.09 | 0.07 | 0.88 | | | | | | Bacteria | Proteobacteria | Alphaproteobacteria | Rickettsiales | Rickettsiales genera incertae | Other |
| | | | | | | | | | 0.04 | | | 0.74 | | | | | | 0.07 | | | | | | Bacteria | Proteobacteria | Gammaproteobacteria | Unclassified order | Unclassified family | Acidibacter |
| | | | 0.48 | | | 0.15 | | | | | | 0.19 | | | | | | | | | | | | Bacteria | Gemmatimonadetes | Gemmatimonadetes | Gemmatimonadales | Gemmatimonadaceae | Gemmatimonas |
| | 0.50 | | | | | | | | | | | 0.12 | | | | | 0.14 | | | | | | | Bacteria | Actinobacteria | Actinobacteridae | Actinomycetales | Micromonosporaceae | Rugosimonospora |
| | | | | | | 0.30 | | | | | | 0.06 | | | | | 0.36 | | | | | | | Bacteria | Actinobacteria | Actinobacteridae | Actinomycetales | Thermomonosporaceae | Actinomadura |
| | | | | | | | | | | | | | | | 0.04 | | | | 0.36 | 0.30 | | | | Bacteria | Planctomycetes | Planctomycetia | Planctomycetales | Planctomycetaceae | Blastopirellula |
| | 0.16 | | | | | | | | | | | | | | | | | 0.15 | | | | | 0.20 | Bacteria | Firmicutes | Erysipelotrichia | Erysipelotrichales | Other | Other |
| | | | | | | | | | | | 0.24 | 0.19 | | | | | 0.07 | | | | | | | Bacteria | Firmicutes | Bacilli | Bacillales | Paenibacillaceae | Cohnella |
| | | | | 0.07 | | | | | 0.26 | | | | | | | | | | | | 0.16 | | | Bacteria | Firmicutes | Clostridia | Clostridiales | Peptococcaceae | Other |
| | | | | | | | 0.05 | | | | | | | | | | | 0.29 | | | | | 0.05 | Bacteria | Proteobacteria | Deltaproteobacteria | Bdellovibrionales | Other | Other |
| | 0.05 | | | | | 0.15 | | | | | | | | | | | | | 0.12 | | | | | Bacteria | Synergistetes | Synergistia | Synergistales | Other | Other |
| | 0.02 | | | | | | | | | | 0.08 | | | | | | | | | | | | 0.20 | Bacteria | Proteobacteria | Deltaproteobacteria | Bdellovibrionales | Bdellovibrionaceae | Other |
| | | | | | | | | 0.05 | 0.15 | | | | | | | | | | | | | 0.04 | | Bacteria | Chloroflexi | Chloroflexia | Chloroflexales | Chloroflexaceae | Chloroflexus |
| | | | | | | | | | 0.04 | | 0.08 | | | | | | 0.07 | | | | | | | Bacteria | Chloroflexi | Anaerolineae | Anaerolineales | Other | Other |
| | 0.02 | | | | | | | | | | | | | | | | | | 0.12 | | | 0.04 | | Bacteria | Firmicutes | Bacilli | Bacillales | Thermoactinomycetaceae | Other |
| | | | | | | | | | | | | 0.19 | | | | 21.01 | | | | | | | | Bacteria | Planctomycetes | Planctomycetia | Planctomycetales | Planctomycetaceae | Gemmata |
| | | | | 6.26 | | | | | | | | | 0.06 | | | | | | | | | | | Bacteria | Proteobacteria | Alphaproteobacteria | Rhizobiales | Methylobacteriaceae | Methylobacterium |
| | | | | | | | | | | | | | | | | | | | | | 4.93 | | 0.05 | Bacteria | Acidobacteria | Solibacteres | Solibacterales | Solibacteraceae | Candidatus Solibacter |
| | | | | | | | | | | | | | | | | | | | | | 4.19 | | 0.20 | Bacteria | Firmicutes | Bacilli | Bacillales | Planococcaceae | Other |
| | | | | | | | | | 0.04 | | | | | | | | | | | | | | 4.07 | Bacteria | Firmicutes | Clostridia | Thermoanaerobacterales | Other | Other |
| | | | | | | | | | | | | | | | | | | | | | 3.20 | | 0.05 | Bacteria | Proteobacteria | Gammaproteobacteria | Enterobacteriales | Enterobacteriaceae | Other |
| | | | | | | | | | | | | | 0.18 | | | | | | 2.79 | | | | | Bacteria | Proteobacteria | Alphaproteobacteria | Rhizobiales | Rhodobiaceae | Other |
| 0.33 | | | | | | | | | | | | | | | | | 2.58 | | | | | | | Bacteria | Proteobacteria | Gammaproteobacteria | Legionellales | Legionellaceae | Legionella |
| | | | | | | | | | | | | | | | | | | | | | 2.63 | | 0.25 | Bacteria | Proteobacteria | Betaproteobacteria | Burkholderiales | Oxalobacteraceae | Undibacterium |
| | | | | | | 0.30 | | | | | | | | | | | | | | | | | 2.01 | Bacteria | Bacteroidetes | Cytophagia | Cytophagales | Other | Other |
| | | | | | | | | | | | | | | | | | | 1.39 | 0.73 | | | | | Archaea | Euryarchaeota | Halobacteria | Halobacteriales | Halobacteriaceae | Halorubrum |
| | | | | | | | | | | | | 0.68 | | | | 1.33 | | | | | | | | Bacteria | Proteobacteria | Alphaproteobacteria | Rhodospirillales | Rhodospirillaceae | Azospirillum |
| | | | | | | | | | | | | | | | | | | 1.47 | | | | | 0.05 | Bacteria | Proteobacteria | Gammaproteobacteria | Aeromonadales | Aeromonadaceae | Aeromonas |
| | | 1.28 | 0.12 | | | | | | | | | | | | | | | | | | | | | Bacteria | Proteobacteria | Alphaproteobacteria | Rhodospirillales | Rhodospirillaceae | Inquilinus |
| | | | | | | | | | | | | | | | | | | 0.15 | | | | | 1.21 | Bacteria | Proteobacteria | Betaproteobacteria | Burkholderiales | Burkholderiaceae | Cupriavidus |
| 1.21 | | | | | | | | | | | | 0.06 | | | | | | | | | | | | Bacteria | Acidobacteria | Acidobacteriia | Acidobacteriales | Acidobacteriaceae | Edaphobacter |
| | | | | | | | | | | | | 0.06 | | | | | | | 0.85 | | | | | Bacteria | Firmicutes | Clostridia | Thermoanaerobacterales | Thermoanaerobacteraceae | Other |
| | | | | | | | | | | | | | | | | | | 0.29 | | 0.60 | | | | Bacteria | Proteobacteria | Gammaproteobacteria | Xanthomonadales | Xanthomonadaceae | Stenotrophomonas |
| | | | | | | 0.60 | | | | | | | | | | 0.22 | | | | | | | | Bacteria | Actinobacteria | Actinobacteridae | Actinomycetales | Microbacteriaceae | Leifsonia |
| | | | | | | | | | | | | | | | 0.36 | | | | | 0.45 | | | | Archaea | Euryarchaeota | Halobacteria | Halobacteriales | Halobacteriaceae | Halonotius |
| | | | | 0.37 | | | | | | 0.36 | | | | | | | | | | | | | | Archaea | Euryarchaeota | Halobacteria | Halobacteriales | Halobacteriaceae | Halolamina |
| 0.55 | | | | | | | | | | | | | | | | | | | | 0.15 | | | | Bacteria | Proteobacteria | Alphaproteobacteria | Sphingomonadales | Sphingomonadaceae | Sphingobium |
| 0.66 | | | | | | | | | | | | | | | 0.04 | | | | | | | | | Bacteria | Proteobacteria | Gammaproteobacteria | Alteromonadales | Alteromonadaceae | Marinobacterium |
| | | | | | | | | | | | | | | | | | | 0.07 | 0.61 | | | | | Bacteria | Proteobacteria | Betaproteobacteria | Burkholderiales | unclassified Burkholderiales | Burkholderiales Genera |
| | | | 0.48 | | 0.16 | | | | | | | | | | | | | | | | | | | Bacteria | Proteobacteria | Deltaproteobacteria | Myxococcales | Polyangiaceae | Chondromyces |
| 0.55 | | | | | | | | | | | | | | | | | | 0.07 | | | | | | Bacteria | Proteobacteria | Alphaproteobacteria | Rhizobiales | Rhizobiaceae | Agrobacterium |
| | 0.02 | | | | | | | | 0.59 | | | | | | | | | | | | | | | Bacteria | Firmicutes | Bacilli | Bacillales | Paenibacillaceae | Other |
| | | | | | | | 0.20 | | | | 0.40 | | | | | | | | | | | | | Bacteria | Actinobacteria | Actinobacteridae | Actinomycetales | Streptosporangiaceae | Streptosporangium |
| | | | | 0.45 | | | 0.15 | | | | | | | | | | | | | | | | | Bacteria | Proteobacteria | Deltaproteobacteria | Desulfobacterales | Desulfobacteraceae | Other |
| | | | | | | | | | | | | | | | | | | 0.51 | | | | | 0.05 | Bacteria | Proteobacteria | Betaproteobacteria | Burkholderiales | Comamonadaceae | Curvibacter |
| | | | 0.12 | | | | | | 0.41 | | | | | | | | | | | | | | | Bacteria | Actinobacteria | Actinobacteridae | Actinomycetales | Geodermatophilaceae | Modestobacter |
| | | | | 0.22 | | | | | | | | | | | | | | | | 0.30 | | | | Archaea | Euryarchaeota | Methanobacteria | Methanobacteriales | Other | Other |
| | | | | | | | | | | | | 0.06 | | | | | | | | 0.45 | | | | Bacteria | Proteobacteria | Gammaproteobacteria | Legionellales | Other | Other |
| | 0.21 | | | | | | | | 0.30 | | | | | | | | | | | | | | | Bacteria | Proteobacteria | Deltaproteobacteria | Myxococcales | Other | Other |
| | | | | | | | | | | | | | | | | | | 0.37 | | | | | 0.05 | Bacteria | Cyanobacteria | Oscillatoriophycideae | Oscillatoriales | Other | Other |
| | 0.14 | 0.21 | | | | | | | | | | | | | | | | | | | | | | Bacteria | Proteobacteria | Alphaproteobacteria | Rhizobiales | Brucellaceae | Ochrobactrum |
| | | | | | | | | | | | | | 0.18 | | | | | 0.15 | | | | | | Bacteria | Proteobacteria | Betaproteobacteria | Burkholderiales | unclassified Burkholderiales | Sphaerotilus |
| | 0.25 | | | | | | | | | | | | | | | | | 0.07 | | | | | | Bacteria | Actinobacteria | Actinobacteridae | Actinomycetales | Streptosporangiaceae | Nonomuraea |
| 0.11 | | | | | | | | | | | | | 0.18 | | | | | | | | | | | Bacteria | Chloroflexi | Dehalococcoidia | Unclassified order | Other | Other |
| | | | | | | | | | | | 0.24 | | | | | | | | | | | 0.04 | | Bacteria | Chloroflexi | Thermoflexia | Thermoflexales | Other | Other |
| | 0.11 | | | | | | | | 0.15 | | | | | | | | | | | | | | | Bacteria | Proteobacteria | Deltaproteobacteria | Myxococcales | Vulgatibacteraceae | Other |
| | | | | | | | | | | | | 0.06 | | | | | | | | | | | 0.15 | Bacteria | Proteobacteria | Alphaproteobacteria | Rhizobiales | Bradyrhizobiaceae | Bosea |
| | | | | | | | | | | | | 0.06 | | | | | | | | | | | 0.15 | Bacteria | Verrucomicrobia | Verrucomicrobiae | Verrucomicrobiales | Verrucomicrobiaceae | Other |
| | | | | | | | | | | | | | 0.06 | | | | | | | 0.15 | | | | Bacteria | Proteobacteria | Gammaproteobacteria | Enterobacteriales | Enterobacteriaceae | Salmonella |
| | | | | 0.15 | | | | | | | | | 0.06 | | | | | | | | | | | Bacteria | Proteobacteria | Gammaproteobacteria | Enterobacteriales | Enterobacteriaceae | Escherichia |
| | | | | | | | | | | | | | | | | | | 0.15 | | | | | 0.05 | Bacteria | Bacteroidetes | Sphingobacteriia | Sphingobacteriales | Chitinophagaceae | Chitinophaga |
| | | | 0.12 | | | | | | 0.07 | | | | | | | | | | | | | | | Bacteria | unclassified Bacteria | Unclassified Class | Unclassified order | Unclassified family | Other |
| | | | | | | | | | | | 0.08 | | | | | | | | | | | 0.11 | | Bacteria | Proteobacteria | Alphaproteobacteria | Rhizobiales | Other | Other |
| | | | | | | | 0.10 | | | | 0.08 | | | | | | | | | | | | | Bacteria | Tenericutes | Mollicutes | Acholeplasmatales | Acholeplasmataceae | Other |
| | | | | | | | | 0.05 | | | | 0.12 | | | | | | | | | | | | Bacteria | Proteobacteria | Alphaproteobacteria | Rhizobiales | unclassified Rhizobiales | Other |
| | 0.11 | | | | | | | | | | | | | | | | | | | | | 0.04 | | Bacteria | Planctomycetes | Planctomycetia | Planctomycetales | Planctomycetaceae | Pirellula |
| | | | | | | | | | | | | | 0.06 | | | | | | | | 0.08 | | | Bacteria | Proteobacteria | Gammaproteobacteria | Enterobacteriales | Other | Other |
| | 0.02 | | | | | | | | | | | | | | 0.11 | | | | | | | | | Bacteria | Actinobacteria | Rubrobacteridae | Solirubrobacterales | Conexibacteraceae | Other |
| | | | | | | | | | | | | | | | | | | 0.07 | | | | | 0.05 | Bacteria | Firmicutes | Bacilli | Bacillales | Bacillaceae | Other |
| | | | | | | | | | | | | | | | | | | 0.07 | | | | | 0.05 | Bacteria | Proteobacteria | Betaproteobacteria | Burkholderiales | Comamonadaceae | Pelomonas |
| | | | | | | | | | | | | | | | | 0.04 | | 0.07 | | | | | | Bacteria | Proteobacteria | Gammaproteobacteria | Thiotrichales | Piscirickettsiaceae | Other |
| | | | | | | | | | | | | | | | | 0.04 | | | | | | | 0.05 | Bacteria | Proteobacteria | Gammaproteobacteria | Chromatiales | Chromatiaceae | Other |
| | | | | | | | | | | | | | | | 0.04 | | | | | | | | 0.05 | Bacteria | Proteobacteria | Gammaproteobacteria | Pseudomonadales | Pseudomonadaceae | Rugamonas |
| | | | | | | | | 98.50 | | | | | | | | | | | | | | | | Bacteria | Actinobacteria | Actinobacteridae | Actinomycetales | Micrococcineae | Curtobacterium |
| | | | | | | | | | | | | | | | | | | | 4.85 | | | | | Bacteria | Planctomycetes | Planctomycetia | Planctomycetales | Planctomycetaceae | Zavarzinella |
| | | | | | | | | | | | | | | | | | | | | | 4.35 | | | Bacteria | Proteobacteria | Deltaproteobacteria | Syntrophobacterales | Syntrophaceae | Desulfomonile |
| | | | | | | | | | | | | | | | | | | | 3.52 | | | | | Bacteria | Actinobacteria | Actinobacteridae | Actinomycetales | Microbacteriaceae | Salinibacterium |
| | | | | | | | | | | | | | | | | | | 3.45 | | | | | | Bacteria | Bacteroidetes | Sphingobacteriia | Sphingobacteriales | Sphingobacteriaceae | Pedobacter |
| | | | | | | | | | | | | | | | | | | 2.64 | | | | | | Archaea | Thaumarchaeota | Nitrososphaerales | Nitrososphaerales | Nitrososphaeraceae | Other |
| | | | | | | | | | | | 2.26 | | | | | | | | | | | | | Bacteria | Proteobacteria | Gammaproteobacteria | Xanthomonadales | Xanthomonadaceae | Rhodanobacter |
| | | | | | | | | | | | | | | | | | | | 1.94 | | | | | Bacteria | Thermotogae | Thermotogae | Thermotogales | Other | Other |
| | | | | | | | | | | | | | | | | | 0.93 | | | | | | | Bacteria | Proteobacteria | Gammaproteobacteria | Alteromonadales | Alteromonadaceae | Other |
| | 0.75 | | | | | | | | | | | | | | | | | | | | | | | Bacteria | Actinobacteria | Actinobacteridae | Actinomycetales | Streptosporangiaceae | Sphaerisporangium |
| | | | | | | | | | | | | | | | | | | 0.73 | | | | | | Bacteria | Proteobacteria | Gammaproteobacteria | Thiotrichales | Piscirickettsiaceae | Thiomicrospira |
| | | | | | | | | | | | | | | | | | | | | 0.60 | | | | Bacteria | Firmicutes | Bacilli | Bacillales | Bacillaceae | Lysinibacillus |
| 0.55 | | | | | | | | | | | | | | | | | | | | | | | | Bacteria | Proteobacteria | Alphaproteobacteria | Rhodospirillales | Acetobacteraceae | Rhodopila |
| | | | | | | | | | | | | | | | | | | | 0.49 | | | | | Bacteria | Chloroflexi | Dehalococcoidia | Unclassified order | Unclassified family | Other |
| | | | | | | | | | | | | | | | | | 0.43 | | | | | | | Bacteria | Armatimonadetes | Chthonomonadetes | Chthonomonadales | Other | Other |
| | | | | | | | | | | | | | | | | | | | | | 0.41 | | | Archaea | Euryarchaeota | Methanomicrobia | Methanosarcinales | Methanosaetaceae | Methanosaeta |
| | | | | | | | | | 0.41 | | | | | | | | | | | | | | | Bacteria | Proteobacteria | Epsilonproteobacteria | Nautiliales | Other | Other |
| | | | | | | | | | | | | 0.37 | | | | | | | | | | | | Bacteria | Proteobacteria | Gammaproteobacteria | Chromatiales | Ectothiorhodospiraceae | Other |
| | | | | | | | | | | | | | | | | | | | 0.36 | | | | | Bacteria | Chloroflexi | Caldilineae | Caldilineales | Caldilineaceae | Caldilinea |
| 0.33 | | | | | | | | | | | | | | | | | | | | | | | | Bacteria | Proteobacteria | Gammaproteobacteria | Pseudomonadales | Moraxellaceae | Acinetobacter |
| | | | | | | | | | | | | | | | | | | | | | 0.33 | | | Bacteria | Proteobacteria | Alphaproteobacteria | Rhizobiales | Hyphomicrobiaceae | Rhodoplanes |
| | | | | | | | | | | | 0.32 | | | | | | | | | | | | | Bacteria | Proteobacteria | Gammaproteobacteria | Xanthomonadales | Xanthomonadaceae | Luteibacter |
| | | | | | | | | | | | | | | | | | | | | 0.30 | | | | Bacteria | Actinobacteria | Actinobacteridae | Actinomycetales | Micrococcaceae | Arthrobacter |
| | | | | | | | | | | | | | | | | | | | | 0.30 | | | | Bacteria | Proteobacteria | Gammaproteobacteria | Xanthomonadales | Xanthomonadaceae | Xanthomonas |
| | | | | 0.30 | | | | | | | | | | | | | | | | | | | | Bacteria | unclassified Bacteria | candidate division NC10 | Unclassified order | Unclassified family | Other |
| | | | | | | 0.30 | | | | | | | | | | | | | | | | | | Bacteria | Firmicutes | Clostridia | Halanaerobiales | Halobacteroidaceae | Natroniella |
| | | | | | | | | | | | | | | | | | | | | | | | 0.25 | Bacteria | Firmicutes | Clostridia | Clostridiales | Symbiobacteriaceae | Other |
| | 0.23 | | | | | | | | | | | | | | | | | | | | | | | Bacteria | Firmicutes | Clostridia | Clostridiales | Clostridiales Family XVII. Inc | Other |
| | 0.23 | | | | | | | | | | | | | | | | | | | | | | | Bacteria | Proteobacteria | Alphaproteobacteria | Rhizobiales | Xanthobacteraceae | Labrys |
| | 0.23 | | | | | | | | | | | | | | | | | | | | | | | Bacteria | Proteobacteria | Deltaproteobacteria | Desulfuromonadales | Pelobacteraceae | Other |
| | | | | | | | | | | | | | | | | | | 0.22 | | | | | | Bacteria | Proteobacteria | Betaproteobacteria | Burkholderiales | Oxalobacteraceae | Pseudoduganella |
| | | | | | | | | | | | | | | | | | | 0.22 | | | | | | Bacteria | Proteobacteria | Gammaproteobacteria | Chromatiales | Other | Other |
| | | 0.21 | | | | | | | | | | | | | | | | | | | | | | Bacteria | Firmicutes | Clostridia | Clostridiales | Syntrophomonadaceae | Other |
| | | | | | | | | | | | | 0.19 | | | | | | | | | | | | Bacteria | Chloroflexi | Ktedonobacteria | Ktedonobacterales | Ktedonobacteraceae | Ktedonobacter |
| | | | | | | | | | | | | | | | | | | | | 0.15 | | | | Archaea | Euryarchaeota | Halobacteria | Halobacteriales | Halobacteriaceae | Halohasta |
| | | | | | | | | | | | | | | | | | | | | 0.15 | | | | Bacteria | Actinobacteria | Thermoleophilia | Thermoleophilales | Thermoleophilaceae | Other |
| | | | | | | | | | | | | | | | | | | | | 0.15 | | | | Bacteria | Chloroflexi | Chloroflexia | Chloroflexales | Oscillochloridaceae | Oscillochloris |
| | | | | | | | | | | | | | | | | | | | | 0.15 | | | | Bacteria | Firmicutes | Clostridia | Halanaerobiales | Halanaerobiaceae | Halanaerobium |
| | | | | | | | | | | | | | | | | | | | | 0.15 | | | | Bacteria | Firmicutes | Negativicutes | Selenomonadales | Veillonellaceae | Megasphaera |
| | | | | | | | | | | | | | | | | | | | | 0.15 | | | | Bacteria | Proteobacteria | Alphaproteobacteria | Rhodospirillales | Other | Other |
| | | | | | | | | | | | | | | | | | | | | 0.15 | | | | Bacteria | Proteobacteria | Betaproteobacteria | Burkholderiales | Alcaligenaceae | Achromobacter |
| | | | | | | | | | | | | | | | | | | | | 0.15 | | | | Bacteria | Proteobacteria | Betaproteobacteria | Burkholderiales | Comamonadaceae | Acidovorax |
| | | | | | | | | | | | | | | | | | | | | | | | 0.15 | Bacteria | Bacteroidetes | Sphingobacteriia | Sphingobacteriales | Chitinophagaceae | Flavisolibacter |
| | | | | | | | | | | | | | | | | | | | | | | | 0.15 | Bacteria | Bacteroidetes | Sphingobacteriia | Sphingobacteriales | Chitinophagaceae | Niastella |
| | | | | | | | | | | | | | | | | | | | | | | | 0.15 | Bacteria | Firmicutes | Bacilli | Lactobacillales | Streptococcaceae | Streptococcus |
| | | | | 0.15 | | | | | | | | | | | | | | | | | | | | Bacteria | unclassified Bacteria | Unclassified Class | Unclassified order | Other | Other |
| | | | | | | 0.15 | | | | | | | | | | | | | | | | | | Bacteria | Chloroflexi | Thermomicrobia | Sphaerobacterales | Sphaerobacteraceae | Sphaerobacter |
| | | | | | | | | | | | | | | | | | 0.14 | | | | | | | Bacteria | Armatimonadetes | Armatimonadia | Armatimonadales | Armatimonadaceae | Other |
| | | | | | | | | | | | | | | | | 0.13 | | | | | | | | Bacteria | Proteobacteria | Alphaproteobacteria | Rhodospirillales | Rhodospirillaceae | Niveispirillum |
| | | | | | | | | | | | | | | | | | | | 0.12 | | | | | Bacteria | Actinobacteria | Actinobacteridae | Actinomycetales | Pseudonocardiaceae | Amycolatopsis |
| | | | | | | | | | | | | | | | | | | | 0.12 | | | | | Bacteria | Firmicutes | Clostridia | Halanaerobiales | Other | Other |
| | | | | | | | | | | | | | | | | | | | 0.12 | | | | | Bacteria | Proteobacteria | Betaproteobacteria | Burkholderiales | unclassified Burkholderiales | Aquabacterium |
| | | | 0.12 | | | | | | | | | | | | | | | | | | | | | Bacteria | Acidobacteria | Acidobacteriia | Acidobacteriales | Acidobacteriaceae | Other |
| | | | | | | | | | | | | | | | | | | | | | | | 0.10 | Bacteria | Verrucomicrobia | Opitutae | Opitutales | Opitutaceae | Alterococcus |
| | | | | | | | | | | | | | | | | 0.09 | | | | | | | | Bacteria | Actinobacteria | Rubrobacteridae | Solirubrobacterales | Solirubrobacteraceae | Other |
| | | | | | | | | | | | | | | | | | | | | | 0.08 | | | Bacteria | Actinobacteria | Rubrobacteridae | Gaiellales | Gaiellaceae | Gaiella |
| | | | | | | | | | | | | | | | | | | | | | 0.08 | | | Bacteria | Firmicutes | Bacilli | Lactobacillales | Carnobacteriaceae | Other |
| | | | | | | | | | | | 0.08 | | | | | | | | | | | | | Bacteria | Actinobacteria | Actinobacteridae | Actinomycetales | Pseudonocardiaceae | Pseudonocardia |
| | | | | | | | | | | | | | | | | | | 0.07 | | | | | | Bacteria | Bacteroidetes | Sphingobacteriia | Sphingobacteriales | Sphingobacteriaceae | Sphingobacterium |
| | | | | | | | | | | | | | | | | | | 0.07 | | | | | | Bacteria | Firmicutes | Clostridia | Clostridiales | Clostridiaceae | Clostridium |
| | | | | | | | | | | | | | | | | | | 0.07 | | | | | | Bacteria | Proteobacteria | Betaproteobacteria | Burkholderiales | Comamonadaceae | Limnohabitans |
| | | | | | | | | | | | | | | | | | | 0.07 | | | | | | Bacteria | Proteobacteria | Betaproteobacteria | Burkholderiales | Comamonadaceae | Polaromonas |
| | | | | | | | | | | | | | | | | | | 0.07 | | | | | | Bacteria | Proteobacteria | Betaproteobacteria | Burkholderiales | Oxalobacteraceae | Collimonas |
| | | | | | | | | | | | | | | | | | | 0.07 | | | | | | Bacteria | Proteobacteria | Betaproteobacteria | Neisseriales | Chromobacteriaceae | Paludibacterium |
| | | | | | | | | | | | | | | | | | | 0.07 | | | | | | Bacteria | Proteobacteria | Deltaproteobacteria | Myxococcales | Labilitrichaceae | Labilithrix |
| | | | | | | | | | | | | | | | | | | 0.07 | | | | | | Bacteria | Proteobacteria | Gammaproteobacteria | Orbales | Orbaceae | Other |
| | | | | | | | | | | | | | | | | | | 0.07 | | | | | | Bacteria | Proteobacteria | Gammaproteobacteria | unclassified Gammaproteob | Unclassified family | Other |
| | | | | | | | | | | | | | | | 0.07 | | | | | | | | | Archaea | Euryarchaeota | Thermoplasmata | Thermoplasmatales | Other | Other |
| | | | | | | | | | | | | 0.06 | | | | | | | | | | | | Bacteria | Actinobacteria | Actinobacteridae | Actinomycetales | Micromonosporaceae | Salinispora |
| | | | | | | | | | | | | 0.06 | | | | | | | | | | | | Bacteria | Proteobacteria | Deltaproteobacteria | Myxococcales | Polyangiaceae | Byssovorax |
| | | | | | | | | | | | | 0.06 | | | | | | | | | | | | Bacteria | Verrucomicrobia | unclassified Verrucomicrobia | Unclassified order | Other | Other |
| | | | | | | | | | | | | | 0.06 | | | | | | | | | | | Archaea | Euryarchaeota | Thermoplasmata | Methanomassiliicoccales | Other | Other |
| | | | | | | | | | | | | | 0.06 | | | | | | | | | | | Bacteria | Actinobacteria | Actinobacteridae | Actinomycetales | Streptomycetaceae | Streptacidiphilus |
| | | | | | | | | | | | | | 0.06 | | | | | | | | | | | Bacteria | Proteobacteria | Betaproteobacteria | Burkholderiales | Comamonadaceae | Caenimonas |
| | | | | | | | | | | | | | | | | | | | | | | | 0.05 | Bacteria | Firmicutes | Bacilli | Bacillales | Planococcaceae | Sporosarcina |
| | | | | | | | | | | | | | | | | | | | | | | | 0.05 | Bacteria | Proteobacteria | Alphaproteobacteria | Rhizobiales | Bradyrhizobiaceae | Afipia |
| | | | | | | | | | | | | | | | | | | | | | | | 0.05 | Bacteria | Proteobacteria | Betaproteobacteria | Burkholderiales | Comamonadaceae | Comamonas |
| | | | | | | | | | | | | | | | | | | | | | | | 0.05 | Bacteria | Synergistetes | Synergistia | Synergistales | Synergistaceae | Other |
| | | | | | | | | | | | | | | | | | | | | | | | 0.05 | Bacteria | unclassified Bacteria | candidate division SR1 | candidate division SR1 bact | Unclassified family | Unclassified genus |
| | | | | | | | | | | | | | | | | 0.04 | | | | | | | | Bacteria | Proteobacteria | Alphaproteobacteria | Rhizobiales | Hyphomicrobiaceae | Rhizomicrobium |
| | | | | | | | | | | | | | | | | | | | | | | 0.04 | | Bacteria | Bacteroidetes | Sphingobacteriia | Sphingobacteriales | Chitinophagaceae | Sediminibacterium |
| | | | | | | | | | 0.04 | | | | | | | | | | | | | | | Bacteria | Acidobacteria | unclassified Acidobacteria | Acidobacteria subdivision 4 | Other | Other |
| | | | | | | | | | 0.04 | | | | | | | | | | | | | | | Bacteria | Actinobacteria | Actinobacteridae | Actinomycetales | Microbacteriaceae | Herbiconiux |
| | | | | | | | | | | | | | | | 0.04 | | | | | | | | | Bacteria | Firmicutes | Clostridia | Clostridiales | Lachnospiraceae | Other |
| | | | | | | | | | | | | | | | 0.04 | | | | | | | | | Bacteria | Proteobacteria | Gammaproteobacteria | Legionellales | Coxiellaceae | Other |
| | | | | | | | | | | | | | | | 0.04 | | | | | | | | | Bacteria | Proteobacteria | Gammaproteobacteria | Methylococcales | Other | Other |
| | 0.02 | | | | | | | | | | | | | | | | | | | | | | | Bacteria | Bacteroidetes | Cytophagia | Cytophagales | Cytophagaceae | Rufibacter |
| | 0.02 | | | | | | | | | | | | | | | | | | | | | | | Bacteria | Firmicutes | unclassified Firmicutes sensu | Unclassified order | Unclassified family | Other |
| | 0.02 | | | | | | | | | | | | | | | | | | | | | | | Bacteria | Proteobacteria | Alphaproteobacteria | Rhizobiales | Xanthobacteraceae | Ancylobacter |

**Supplementary Figure S6: Abundance and distribution of all genera found in wheat root-associated samples by pyrotags.** All root-associated sequences were taxonomically assigned by BLASTN against NCBI 16S rRNA and were classified to genus (>90% similarity to best-hit), family or order (depending on the case for hits <90% similarity to best-hit). Sequences were grouped by sample, site and classification and relative abundances were calculated according to total number of sequences per sample. Color scales indicate the relative abundances, with a gradual transition between green (high abundant) to red (low abundant).

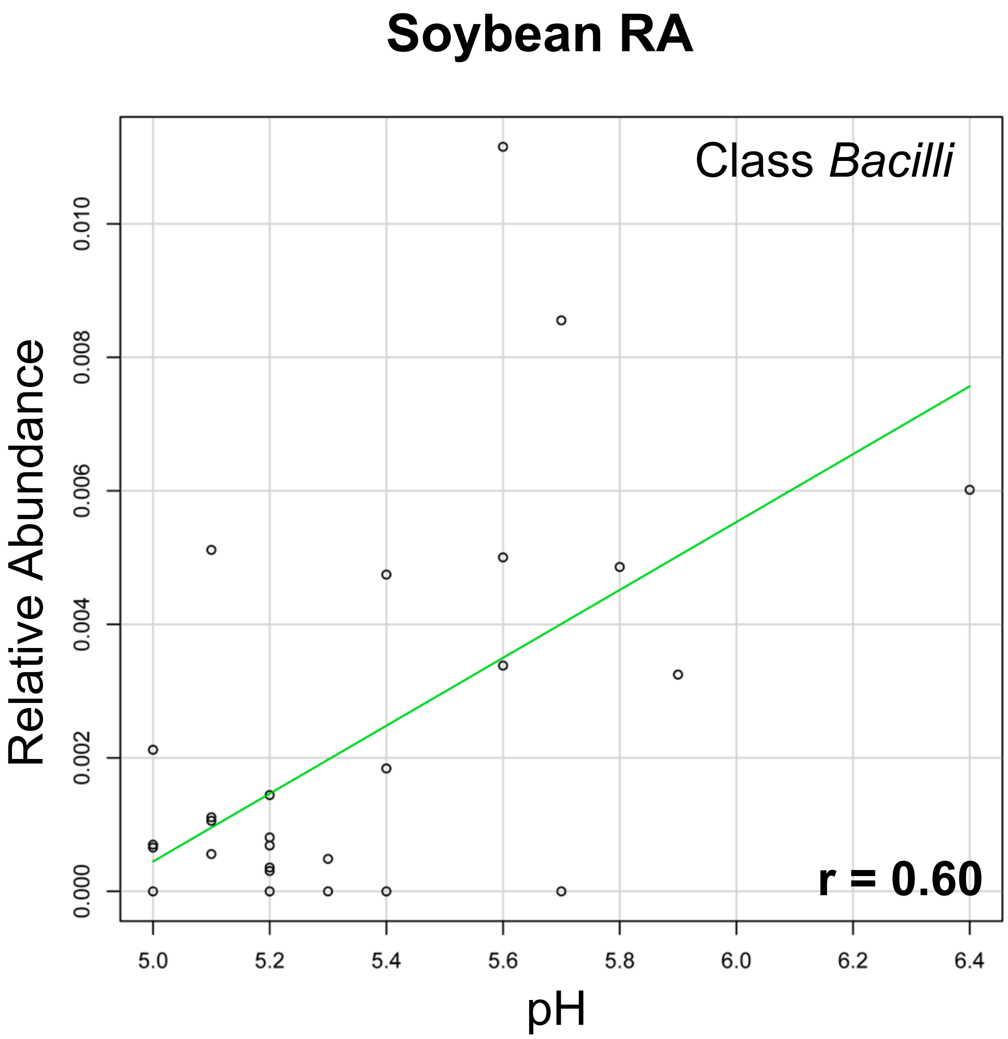


**Supplementary Figure S7: Correlation between taxa abundances and soil characteristics.** Significant Pearson correlations ($p \leq 0.05$) between relative abundances of Bacilli class and pH in soybean root-associated fraction (RA).

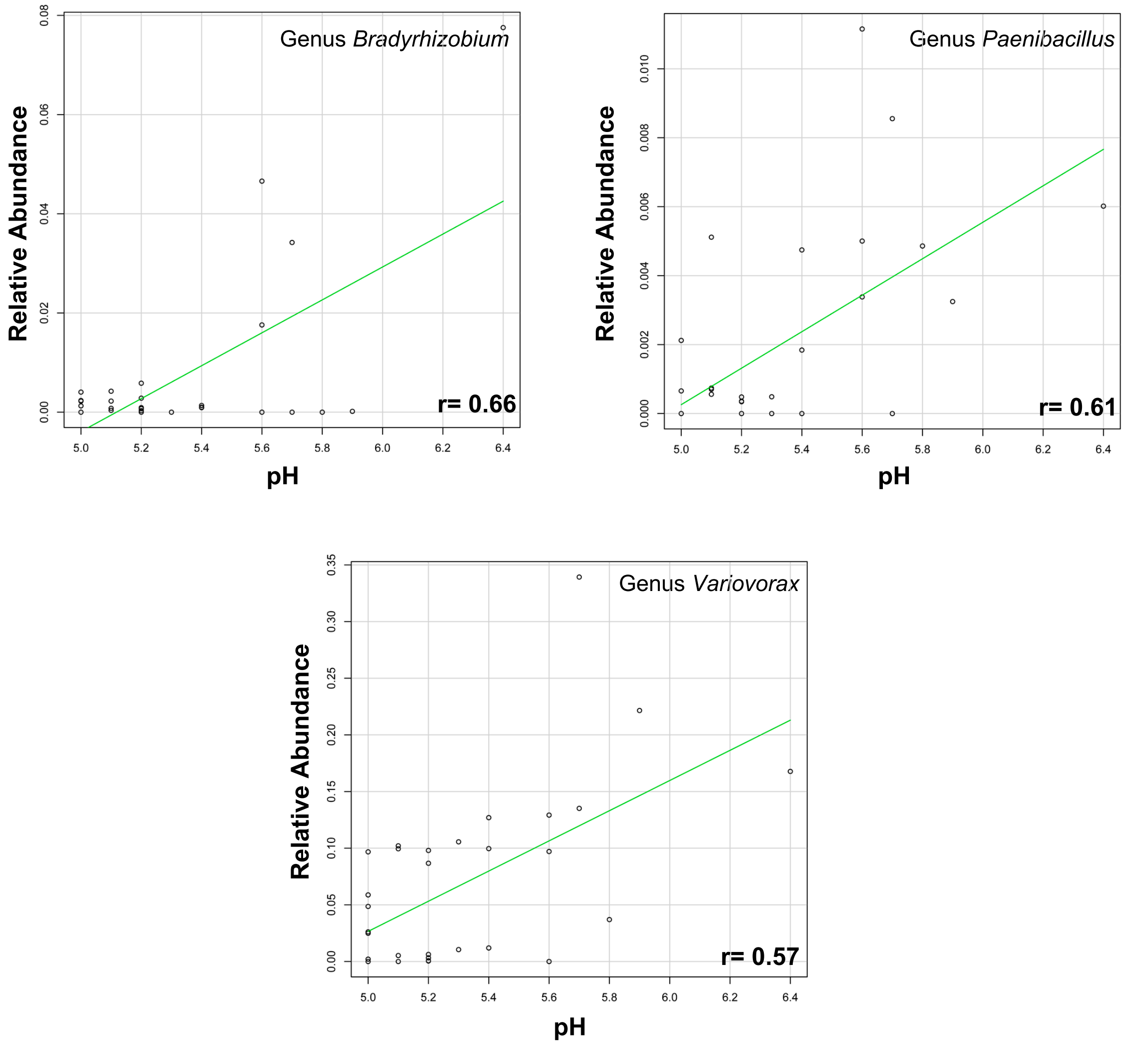


**Supplementary Figure S8: Correlation between taxa abundances and soil characteristics.** Significant Pearson correlations ($p \leq 0.05$) between relative abundances of taxa (Genus level) in soybean root-associated microbiomes and pH.

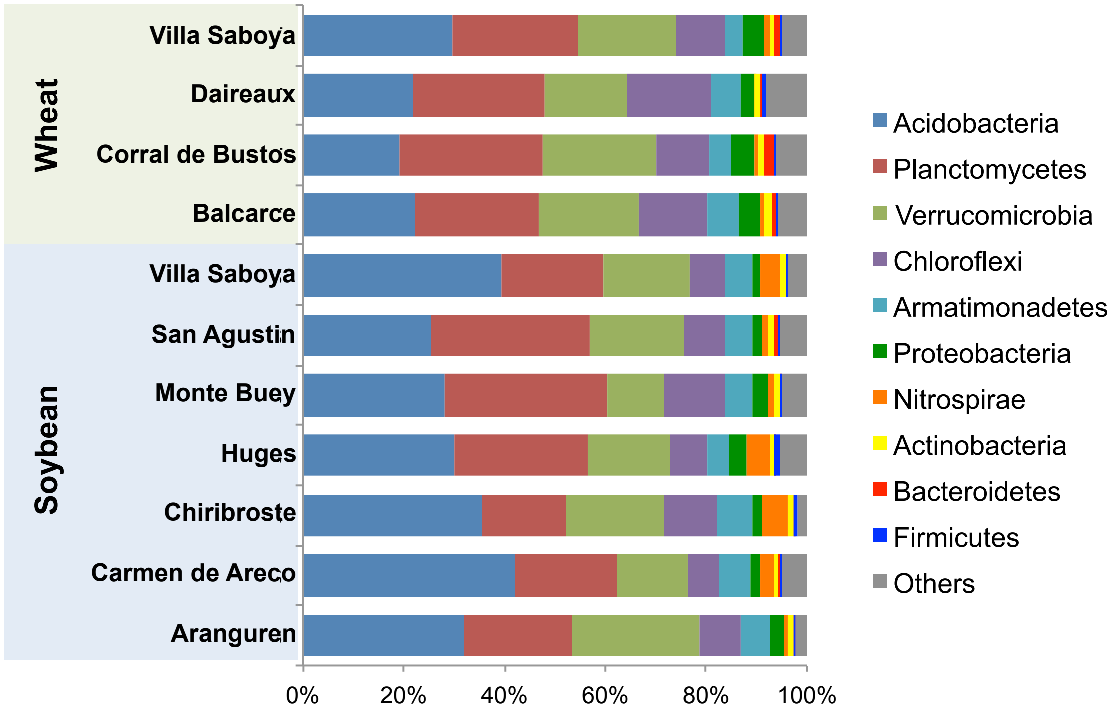


**Supplementary Figure S9: Taxonomic classification of RS pyrotag datasets.** Pyrotag sequences from rhizospheric soil samples were classified at the phylum level. Average values for each site and crop type are shown. An unexpected low abundance of Proteobacteria was observed in all samples and this result is discussed in *supplementary text*.

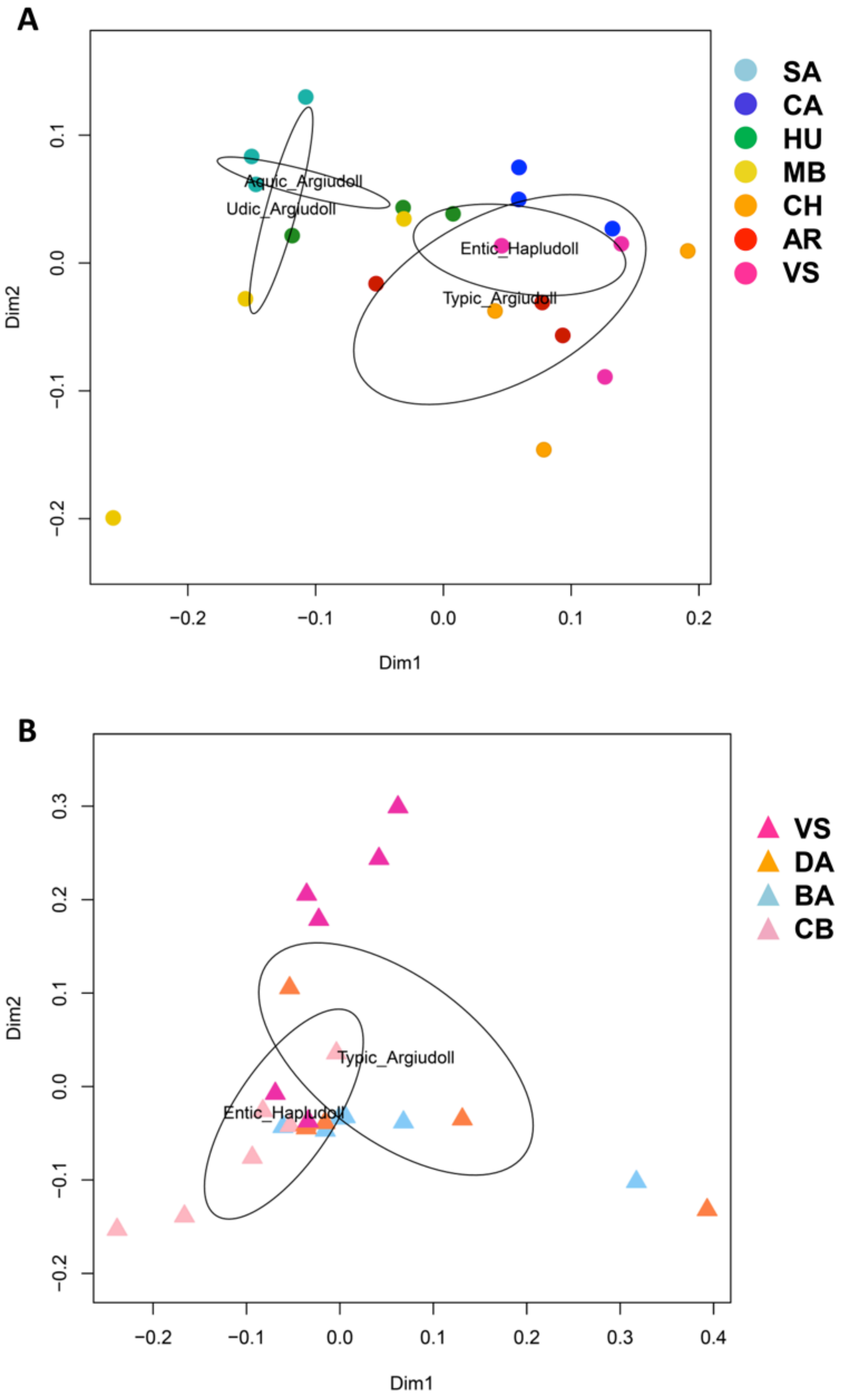


**Supplementary Figure S10: Principal Coordinate Analysis visualization based on weighted UniFrac distances.** Microbial community assemblies of soybean RS samples (A) and wheat RA samples (B) were significantly

structured by soil type. The fitting of factors was done using factorfit() function in “vegan” R package using 100 permutations. Dispersion ellipses represent standard deviation of point scores for each type of soil. AR: Aranguren, CA: Carmen de Areco, CH: Chilibroste, HU: Huges, MB: Monte Buey, SA: San Agustin, VS: Villa Saboya, BA: Balcarce, CB: Corral de Bustos, DA: Daireaux

A

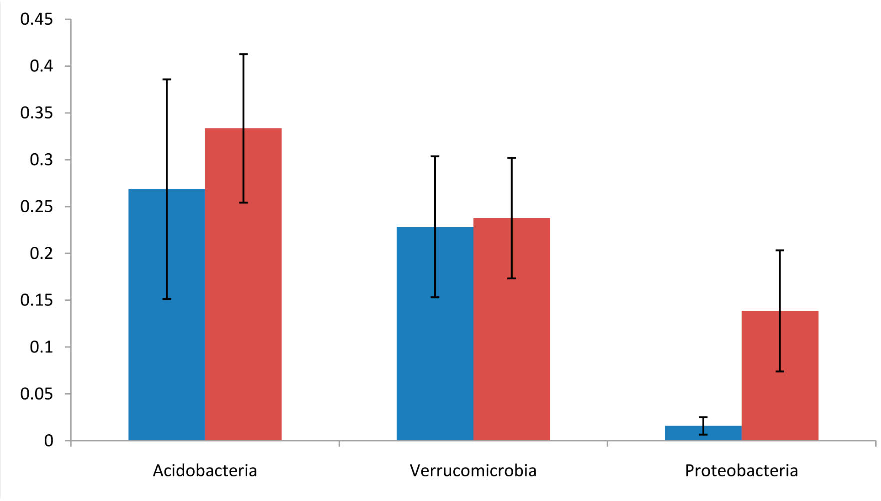


B

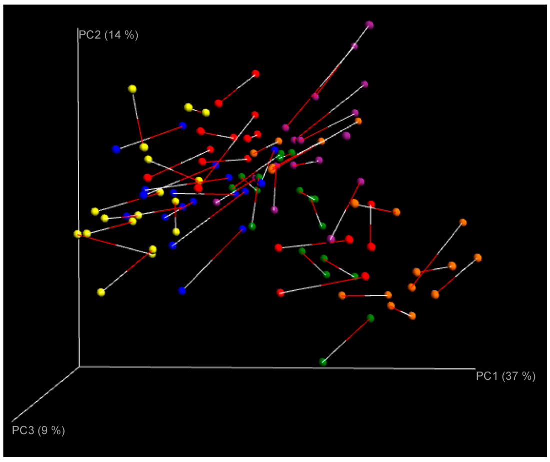


**Supplementary Figure S11: F515-R806 vs. F563-R602 amplification primers.** Differences in taxa abundances for external soil microbiome datasets amplified with 515F- 806R (blue bars) and 563F- 802R (red bars) tagged primers (A). External dataset were compared using Procrustes analyses of Principal Coordinate plots based on weighted UniFrac distances (B). Results indicated that beta diversity conclusions were highly reproducible ($p<0.0001$) regardless the primer sets used for amplification. This figure is discussed in *supplementary text*.

| Location | Crop | Soil classification | pH | Nitrates (ppm) | Phosphorus (ppm) | Organic Matter (%) | Total Nitrogen (%) | Carbon (%) |
|---|---|---|---|---|---|---|---|---|
| **Balcarce (BA)** | Wheat | Typic Argiudoll | 5.097 | 5.186 | 23.114 | 4.026 | 0.192 | 2.413 |
| **Daireaux (DA)** | Wheat | Entic Hapludoll | 4.996 | 5.514 | 17.300 | 1.126 | 0.120 | 0.650 |
| **San Jorge (SJ)** | Wheat | Typic Argiudoll | 6.143 | 2.286 | 33.486 | 2.520 | 0.126 | 1.444 |
| **Corral de Bustos (CB)** | Wheat | Typic Argiudoll | 5.829 | 5.786 | 35.429 | 2.614 | 0.130 | 1.506 |
| | Soybean | | 5.267 | 3.067 | 16.300 | 2.267 | 0.112 | 1.307 |
| **Villa Saboya (VS)** | Wheat | Entic Hapludoll | 5.471 | 2.686 | 7.771 | 1.920 | 0.096 | 1.100 |
| | Soybean | | 5.175 | 18.100 | 6.475 | 1.825 | 0.089 | 1.035 |
| **Monte Buey (MB)** | Wheat | Typic Argiudoll | 5.929 | 2.357 | 75.729 | 3.229 | 0.161 | 1.829 |
| | Soybean | | 5.067 | 4.300 | 171.667 | 2.533 | 0.126 | 1.373 |
| **Aranguren (AR)** | Soybean | Aquic Argiudoll | 5.100 | 6.867 | 24.433 | 4.000 | 0.200 | 2.317 |
| **Carmen de Areco (CA)** | Soybean | Typic Argiudoll | 5.900 | 6.733 | 32.667 | 2.867 | 0.144 | 1.673 |
| **Chilibroste (CH)** | Soybean | Udic Argiudoll | 5.767 | 4.267 | 19.033 | 2.033 | 0.102 | 1.177 |
| **Hughes (HU)** | Soybean | Typic Argiudoll | 5.167 | 6.633 | 11.067 | 3.533 | 0.177 | 2.057 |
| **San Agustin (SA)** | Soybean | Typic Argiudoll | 5.167 | 4.033 | 25.933 | 5.117 | 0.255 | 3.053 |

**Supplementary Table S1: Soil chemical characteristics from sampled sites.** Chemical characteristics (mean) of soils in sampled sites.

(See Supplementary Comma separated file for Supplementary Table S2)

**Supplementary Table S2: List of all isolates obtained in this study organized by crop and isolation strategy.** The table includes also the assigned taxonomy, the results from PGP studies (when available) and the obtained 16S rRNA sequences.

| | Type | Root-Associated bacteria | Rhizospheric soil | | | |
|---|---|---|---|---|---|---|
| | Growth condition | Nutritive broth | Sporulating | Cetrimide | NFB | Total |
| Crop | Site | | | | | |
| Wheat | Balcarce | 73 | 82 | 9 | 94 | 258 |
| | Corral de Bustos | 77 | 64 | 0 | 42 | 183 |
| | Daireaux | 20 | 58 | 0 | 29 | 107 |
| | Monte Buey | 63 | 31 | 0 | 63 | 157 |
| | San Jorge | 18 | 5 | 0 | 45 | 68 |
| | Villa Saboya | 68 | 43 | 63 | 25 | 199 |
| | Subtotal | 319 | 283 | 72 | 298 | 972 |
| Soybean | Aranguren | 38 | 38 | 16 | 29 | 121 |
| | Carmen de Areco | 38 | 38 | 17 | 37 | 130 |
| | Chilibroste | 30 | 38 | 14 | 30 | 112 |
| | Corral de Bustos | 36 | 40 | 11 | 32 | 119 |
| | Hughes | 41 | 37 | 13 | 26 | 117 |
| | Monte Buey | 35 | 37 | 13 | 38 | 123 |
| | San Agustin | 38 | 31 | 13 | 30 | 112 |
| | Villa Saboya | 39 | 42 | 12 | 32 | 125 |
| | Subtotal | 295 | 301 | 109 | 254 | 959 |
| | Total | 614 | 584 | 181 | 552 | 1,931 |

**Supplementary Table S3: Number of isolates obtained by isolation strategy and crop.**

| Soybean Root Asociated | | | | | | |
|---|---|---|---|---|---|---|
| Comparison | | Estimator | Lower | Upper | Statistic | p.Value |
| AR | CA | 0.75 | 0.114 | 0.986 | 1.0422351 | 0.9931536 |
| AR | CH | 0.125 | 0.003 | 0.88 | -1.4745567 | 0.8899356 |
| AR | HU | 0.999 | 0.994 | 1 | 11.5456541 | **0.0000000** |
| AR | MB | 0.562 | 0.062 | 0.962 | 0.2527084 | 1.0000000 |
| AR | SA | 0.906 | 0.163 | 0.998 | 1.7330537 | 0.7292847 |
| AR | VS | 0.844 | 0.151 | 0.994 | 1.4728405 | 0.8908373 |
| CA | CH | 0.001 | 0 | 0.006 | -11.5456541 | **0.0000000** |
| CA | HU | 0.999 | 0.994 | 1 | 11.5456541 | **0.0000000** |
| CA | MB | 0.375 | 0.024 | 0.935 | -0.478899 | 0.9999993 |
| CA | SA | 0.781 | 0.144 | 0.987 | 1.2436594 | 0.9677446 |
| CA | VS | 0.688 | 0.073 | 0.984 | 0.7077098 | 0.9998934 |
| CH | HU | 0.999 | 0.994 | 1 | 11.5456541 | **0.0000000** |
| CH | MB | 0.812 | 0.083 | 0.995 | 1.1302602 | 0.9856282 |
| CH | SA | 0.999 | 0.994 | 1 | 11.5456541 | **0.0000000** |
| CH | VS | 0.969 | 0.285 | 1 | 2.3523145 | 0.2677536 |
| HU | MB | 0.094 | 0.002 | 0.837 | -1.7330537 | 0.7293153 |
| HU | SA | 0.125 | 0.003 | 0.88 | -1.4745567 | 0.8901406 |
| HU | VS | 0.188 | 0.008 | 0.864 | -1.3198596 | 0.9489530 |
| MB | SA | 0.75 | 0.104 | 0.987 | 1.0091398 | 0.9949752 |
| MB | VS | 0.688 | 0.091 | 0.98 | 0.7615348 | 0.9997497 |
| SA | VS | 0.469 | 0.046 | 0.942 | -0.1280535 | 1.0000000 |

| Wheat Root Asociated | | | | | | |
|---|---|---|---|---|---|---|
| Comparison | | Estimator | Lower | Upper | Statistic | p.Value |
| BA | CB | 0.236 | 0.029 | 0.763 | -1.2416262 | 0.5830740 |
| BA | DA | 0.264 | 0.038 | 0.766 | -1.1502104 | 0.6469206 |
| BA | VS | 0.833 | 0.203 | 0.99 | 1.3411983 | 0.5137551 |
| CB | DA | 0.569 | 0.167 | 0.897 | 0.3682761 | 0.9904195 |
| CB | VS | 0.833 | 0.203 | 0.99 | 1.3411983 | 0.5141363 |
| DA | VS | 0.847 | 0.227 | 0.991 | 1.4453134 | 0.4440308 |

| Soybean rhizospheric soil | | | | | | |
|---|---|---|---|---|---|---|
| Comparison | | Estimator | Lower | Upper | Statistic | p.Value |
| AR | CA | 0.889 | 0.071 | 0.999 | 1.3070109 | 0.88221290 |
| AR | CH | 0.444 | 0.019 | 0.971 | -0.1753181 | 1.00000000 |
| AR | HU | 0.833 | 0.08 | 0.997 | 1.1615118 | 0.93819000 |
| AR | MB | 0.889 | 0.071 | 0.999 | 1.3070109 | 0.88222240 |
| AR | SA | 0.889 | 0.071 | 0.999 | 1.3070109 | 0.88249640 |
| AR | VS | 0.556 | 0.029 | 0.981 | 0.1753181 | 1.00000000 |
| CA | CH | 0.222 | 0.004 | 0.95 | -0.8715041 | 0.99015420 |
| CA | HU | 0.333 | 0.006 | 0.976 | -0.4620981 | 0.99995180 |
| CA | MB | 0.333 | 0.006 | 0.976 | -0.4620981 | 0.99995210 |
| CA | SA | 0.444 | 0.019 | 0.971 | -0.1753181 | 1.00000000 |
| CA | VS | 0.111 | 0.001 | 0.929 | -1.3070109 | 0.88259230 |
| CH | HU | 0.999 | 0.993 | 1 | 9.9988297 | **0.00000000** |
| CH | MB | 0.833 | 0.08 | 0.997 | 1.1615118 | 0.93772490 |
| CH | SA | 0.778 | 0.05 | 0.996 | 0.8715041 | 0.99026860 |
| CH | VS | 0.556 | 0.029 | 0.981 | 0.1753181 | 1.00000000 |
| HU | MB | 0.667 | 0.024 | 0.994 | 0.4620981 | 0.99995200 |
| HU | SA | 0.556 | 0.029 | 0.981 | 0.1753181 | 1.00000000 |
| HU | VS | 0.222 | 0.004 | 0.95 | -0.8715041 | 0.99021640 |
| MB | SA | 0.444 | 0.019 | 0.971 | -0.1753181 | 1.00000000 |
| MB | VS | 0.111 | 0.001 | 0.929 | -1.3070109 | 0.88295530 |
| SA | VS | 0.111 | 0.001 | 0.929 | -1.3070109 | 0.88288630 |

**Supplementary Table S4:** Tukey non-parametric Test was used to compare OUT richness between pairs of sampled sites: AR: Aranguren, CA: Carmen de Areco, CH: Chilibroste, HU: Huges, MB: Monte Buey, SA: San Agustin, VS: Villa Saboya, BA: Balcarce, CB: Corral de Bustos, DA: Daireaux.

| P solubilization | | |
|---|---|---|
| Crop | Fraction | |
| | RA | RS |
| Soybean | 54.72 | 20.63 |
| Wheat | 45.28 | 79.37 |
| Soyben+Wheat | 35.93 | 64.07 |

| IAA production | | |
|---|---|---|
| Crop | Fraction | |
| | RA | RS |
| Soybean | 50.85 | 42.53 |
| Wheat | 49.15 | 57.47 |
| Soyben+Wheat | 40.41 | 59.59 |

| ACC deaminase | | |
|---|---|---|
| Crop | Fraction | |
| | RA | RS |
| Soybean | 87.76 | 78.95 |
| Wheat | 12.24 | 21.05 |
| Soyben+Wheat | 46.23 | 53.77 |

| N fixation | | |
|---|---|---|
| Crop | Fraction | |
| | RA | RS |
| Soybean | 53.85 | 54.30 |
| Wheat | 46.15 | 45.70 |
| Soyben+Wheat | 46.26 | 53.74 |

**Supplementary Table S5:** Relative abundance of PGP traits positive isolates by fraction and crop. Results are indicated as percentages (%).

## Scripts used:

Here we list the scripts and commands used to analyze DNA sequencing data. The filename used for each script (in bold and underline) is followed by the content of each script. QIIME mapping file is listed at the end of the list.

## **scripts_and_commands_used.sh**

```
#!/bin/bash


# Quality filtering, denoising, chimera checking and sequence split by barcode

ampliconnoise.py -i ../00_Rawdata/H7M6N7102_run73.sff.txt -o run1_amp_noi.fna -m
mapping_run1_pool1.txt --seqnoise_resolution 25.0 -f --platform titanium
ampliconnoise.py -i ../00_Rawdata/IDC449H01_Run79.sff.txt -o run2_pool1_amp_noi.fna -m
mapping_run2_pool1.txt --seqnoise_resolution 25.0 -f --platform titanium
ampliconnoise.py -i ../00_Rawdata/IDC449H02_Run79.sff.txt -o run2_pool2_amp_noi.fna -m
mapping_run2_pool2.txt --seqnoise_resolution 25.0 -f --platform titanium

# Create Directories to compare each group of samples and for each analysis

mkdir 10_soybean_RA_RS
mkdir 11_wheat_RA_RS
mkdir 12_soybean_RA
mkdir 13_soybean_RS
mkdir 14_wheat_RA
mkdir 15_wheat_RS
mkdir 16_wheat_soybean_RA_RS
mkdir 17_wheat_soybean_RA
mkdir 20_RA_pyro_vs_isol


# Create Mapping files for each QIIME analysis

cat mapping_file.txt | awk -F\t '$16 != "Non"' > 10_soybean_RA_RS/mapping_file.txt
cat mapping_file.txt | awk -F\t '$17 != "Non"' > 11_wheat_RA_RS/mapping_file.txt
cat mapping_file.txt | awk -F\t '$18 != "Non"' > 12_soybean_RA/mapping_file.txt
cat mapping_file.txt | awk -F\t '$19 != "Non"' > 13_soybean_RS/mapping_file.txt
cat mapping_file.txt | awk -F\t '$20 != "Non"' > 14_wheat_RA/mapping_file.txt
cat mapping_file.txt | awk -F\t '$21 != "Non"' > 15_wheat_RS/mapping_file.txt
cat mapping_file.txt | awk -F\t '$22 != "Non"' > 16_wheat_soybean_RA_RS/mapping_file.txt
cat mapping_file.txt | awk -F\t '$23 != "Non"' > 17_wheat_soybean_RA/mapping_file.txt

# Filter full fasta file with selected sequences

filter_fasta.py -f all_sequences_qc_filtered.fna --mapping_fp 10_soybean_RA_RS/mapping_file.txt --
valid_states Folder10:10_soybean_RA_RS -o 10_soybean_RA_RS/seqs.fna
filter_fasta.py -f all_sequences_qc_filtered.fna --mapping_fp 11_wheat_RA_RS/mapping_file.txt --
valid_states Folder11:11_wheat_RA_RS -o 11_wheat_RA_RS/seqs.fna
filter_fasta.py -f all_sequences_qc_filtered.fna --mapping_fp 12_soybean_RA/mapping_file.txt --
valid_states Folder12:12_soybean_RA -o 12_soybean_RA/seqs.fna
filter_fasta.py -f all_sequences_qc_filtered.fna --mapping_fp 13_soybean_RS/mapping_file.txt --
valid_states Folder13:13_soybean_RS -o 13_soybean_RS/seqs.fna
```

```
filter_fasta.py -f all_sequences_qc_filtered.fna --mapping_fp 14_wheat_RA/mapping_file.txt --
valid_states Folder14:14_wheat_RA -o 14_wheat_RA/seqs.fna
filter_fasta.py -f all_sequences_qc_filtered.fna --mapping_fp 15_wheat_RS/mapping_file.txt --
valid_states Folder15:15_wheat_RS -o 15_wheat_RS/seqs.fna
filter_fasta.py -f all_sequences_qc_filtered.fna --mapping_fp 16_wheat_soybean_RA_RS/mapping_file.txt
--valid_states Folder16:16_wheat_soybean_RA_RS -o 16_wheat_soybean_RA_RS/seqs.fna
filter_fasta.py -f all_sequences_qc_filtered.fna --mapping_fp 17_wheat_soybean_RA/mapping_file.txt --
valid_states Folder17:17_wheat_soybean_RA -o 17_wheat_soybean_RA/seqs.fna

# Run QIIME analysis for all samples

for i in
{10_soybean_RA_RS,11_wheat_RA_RS,12_soybean_RA,13_soybean_RS,14_wheat_RA,15_wheat_RS,16_wheat_soybean
_RA_RS,17_wheat_soybean_RA}; do cd $i ; cp ../qiime_custom_pipeline.sh . ; cp
../custom_parameters.txt . ; sh qiime_custom_pipeline.sh 2> error.log; cd ..; done

# Trimming isolates Sanger sequences

blastn -task blastn -query all_seqs_raw.fna -db 16SMicrobial -max_target_seqs 1 -outfmt '6 qseqid
qlen length qseq' | tr -d '-' | awk '$3 >= 180' | awk '{print ">"$1" initial_length="$2"
trimmed_length="$3"\t"$4}' | tr '\t' '\n' | fold > all_isolates_seqs_trimmed.fna

# Classify sequences based on BlastN against NCBI
# (All pyrotags sequences and all isolates sequences, without clustering in OTU, were blasted with
this command.
# Classifications of representative sequences were retrieved from these results)

blastn -task blastn -query isolates_or_pyrotag_sequences.fna -db 16SMicrobial -max_target_seqs 1 -
evalue 1E-10 -outfmt '6 qseqid qstart qend qlen sseqid sstart send slen length pident evalue score
staxids stitle' -num_threads 5 -out sequences_vs_NCBI_16S.blastn

# Comparison between pyrotags sequences from Root-Associated samples (soybean 67,373 / wheat 38,870)
vs. all isolates sequences obtained (2007: 960 from soybean, 1047 from wheat).

makeblastdb -in all_isolates_both_crops.fna -dbtype nucl -parse_seqids

blastn -task blastn -query 12_soybean_RA/seqs.fna -db all_isolates_both_crops.fna -max_target_seqs 1
-evalue 1E-10 -outfmt '6 qseqid qstart qend qlen sseqid sstart send slen length pident evalue score'
-max_target_seqs 1 -num_threads 5 -out
20_RA_pyro_vs_isol/soybean_RA_vs_all_isolates_both_crops.blastn
blastn -task blastn -query 14_wheat_RA/seqs.fna -db all_isolates_both_crops.fna -max_target_seqs 1 -
evalue 1E-10 -outfmt '6 qseqid qstart qend qlen sseqid sstart send slen length pident evalue score' -
max_target_seqs 1 -num_threads 5 -out 20_RA_pyro_vs_isol/wheat_RA_vs_all_isolates_both_crops.blastn

OVER97WHEAT=`cat 20_RA_pyro_vs_isol/soybean_RA_vs_all_isolates_both_crops.blastn | awk '$9/$4 >= 0.8
&& $10 >= 97' | wc -l`
COUNTWHEAT=`cut -f 1 20_RA_pyro_vs_isol/soybean_RA_vs_all_isolates_both_crops.blastn | sort -u | wc -
l`
PERCOVER97=$(bc <<<"scale=3;$OVER97WHEAT/$COUNTWHEAT")

echo "Number of Wheat RA pyrotag sequences with over 97% similarity to best hit in isolates:
"$OVER97WHEAT
echo "Total number Wheat RA pyrotag sequences analyzed: "$COUNTWHEAT
echo "Percentage Wheat RA pyrotags with hits over 97% similarity to an isolate= 0"$PERCOVER97

OVER97SOYBEAN=`cat 20_RA_pyro_vs_isol/wheat_RA_vs_all_isolates_both_crops.blastn | awk '$9/$4 >= 0.8
&& $10 >= 97' | wc -l`
COUNTSOYBEAN=`cut -f 1 20_RA_pyro_vs_isol/wheat_RA_vs_all_isolates_both_crops.blastn | sort -u | wc -
l`
```

```
PERCOVER97=$(bc <<<"scale=3;$OVER97SOYBEAN/$COUNTSOYBEAN")

echo "Number of Soybean RA pyrotag sequences with over 97% similarity to best hit in isolates: 
"$OVER97SOYBEAN
echo "Total number Soybean RA pyrotag sequences analyzed: "$COUNTSOYBEAN
echo "Percentage Soybean RA pyrotags with hits over 97% similarity to an isolate= 0"$PERCOVER97

# Calculate histograms for the distributions of weigthed Unifrac distances

	# 1) Retrieve the weigthed unifrac distances matrices from each of the 100 repetitions, keep only the upper triangle (not including zeros at the diagonal) and put all the information in a single file
for i in {12_soybean_RA,13_soybean_RS,14_wheat_RA,15_wheat_RS}; do for j in {0..99}; do NUMSAMP=`grep -v '#' ${i}/mapping_*.txt | wc -l`; cat ${i}/97_percent_similarity/60_beta_diversity/weighted_unifrac/rare_dm/weighted_unifrac_rarefaction_400_${j}.txt | cut -f 2- | tail -n $NUMSAMP | awk '{for (i=1;i<=NF;i++) printf "%s%s",(i>NR)?$i:"",FS="\t"; print""}' | tr '\t' '\n' | grep '.' | sort -n >> ${i}/values_WU_distances.txt; done; done
	# 2) Make histograms with values obtained from the 100 repetitions
for i in {12_soybean_RA,13_soybean_RS,14_wheat_RA,15_wheat_RS}; do awk -f histograms.awk 0 1 0.01 ${i}/histogram_WU_distances.txt ${i}/values_WU_distances.txt; done
	# 3) Resulting tables are imported to Excel, abundances are transformed into relative values and plotted
```

## qiime_custom_pipeline.sh

```
#!/bin/bash

DIR="$( cd "$( dirname "$0" )" && pwd )"

LEVELS=97        #here other similarity levels may be selected

cd $DIR

echo -e "Step 0 - Determine MIN, MAX and NUM_SAMPLES values"
MIN=`cat seqs.fna | grep '>' | cut -f 1 -d '_' | sort | uniq -c | awk '{print $1}' | sort -n | head -
n 1`
MAX=`cat seqs.fna | grep '>' | cut -f 1 -d '_' | sort | uniq -c | awk '{print $1}' | sort -n | tail -
n 1`
NUM_SAMPLES=`cat seqs.fna | grep '>' | cut -f 1 -d '_' | sort | uniq -c | wc -l`

echo -e "\t\tMIN: $MIN"
echo -e "\t\tMAX: $MAX"
echo -e "\t\tNUM_SAMPLES: $NUM_SAMPLES"

# ### Cluster sequences in OTU and chose the representative sequence
echo -e "Step 1 - Cluster sequences in OTU and chose the representative sequences"
for i in $LEVELS
do
        echo -e "\tSIMILARITY: $i"
        SIM=${i}_percent_similarity
        mkdir -p $SIM/10_pick_otus/
        echo -e "\t\tStep 1.1 - Pick OTUs"
        pick_otus.py -i seqs.fna -s 0.${i} -m uclust -o $SIM/10_pick_otus/ -A
        echo -e "\t\tStep 1.2 - Pick representative set"
        pick_rep_set.py -i $SIM/10_pick_otus/seqs_otus.txt -m most_abundant -f seqs.fna -o
$SIM/10_pick_otus/seqs_rep_set.fasta
done

# ### Align representative sequences and generate phylogenetic analysis
echo -e "Step 2 - Align representative sequences and generate phylogenetic analysis"
for i in $LEVELS
do
        echo -e "\tSIMILARITY: $i"
        SIM=${i}_percent_similarity
        mkdir -p $SIM/20_make_phylogeny/
        echo -e "\t\tStep 2.1 - Align seqs"
        align_seqs.py -i $SIM/10_pick_otus/seqs_rep_set.fasta -p 0.75 -m pynast -a uclust -e 150 -o
$SIM/20_make_phylogeny/pynast_fasttree_method/
        echo -e "\t\tStep 2.2 - Filter alignment"
        filter_alignment.py -i
$SIM/20_make_phylogeny/pynast_fasttree_method/seqs_rep_set_aligned.fasta -o
$SIM/20_make_phylogeny/pynast_fasttree_method/
        echo -e "\t\tStep 2.3 - Make phylogeny"
        make_phylogeny.py -i
$SIM/20_make_phylogeny/pynast_fasttree_method/seqs_rep_set_aligned_pfiltered.fasta -o
$SIM/20_make_phylogeny/pynast_fasttree_method/seqs_rep_set_aligned_pfiltered.tre
done

# ### Assign taxonomy (This is only exploratory and was done to have an initial overview of the
taxonomic distribution)
echo -e "Step 3 - Assign taxonomy"
```

```
for i in $LEVELS
do
	echo -e "\tSIMILARITY: $i"
	SIM=${i}_percent_similarity
	mkdir -p $SIM/30_assign_taxonomy/rdp_method/
	echo -e "\t\tStep 3.1 - Assign taxonomy"
	assign_taxonomy.py -m rdp -c 0.7 --rdp_max_memory=10000 -i
$SIM/10_pick_otus/seqs_rep_set.fasta -o $SIM/30_assign_taxonomy/rdp_method/
done

# ### Create OTU tables
echo -e "Step 4 - Create OTU tables"
STEP=$((($MAX-$MIN)/10))  # Typically this calculation would be used to calculate the step. Here we
used 200 as a fix number
for i in $LEVELS
do
	echo -e "\tSIMILARITY: $i"
	SIM=${i}_percent_similarity
	mkdir -p $SIM/40_otu_tables/rdp_method/
	echo -e "\t\tStep 4.1 - Make OTU table"
	make_otu_table.py -i $SIM/10_pick_otus/seqs_otus.txt -t
$SIM/30_assign_taxonomy/rdp_method/seqs_rep_set_tax_assignments.txt -o
$SIM/40_otu_tables/rdp_method/seqs_otu_table.biom
	echo -e "\t\tStep 4.2 - Convert biom"
	biom convert --table-type "OTU table" --process-obs-metadata "taxonomy" --to-tsv --header-
key "taxonomy" -i $SIM/40_otu_tables/rdp_method/seqs_otu_table.biom -o
$SIM/40_otu_tables/rdp_method/seqs_otu_table.txt
	echo -e "\t\tStep 4.3 - Multiple rarefactions"
#	Normally we would use the following commnand:
#	multiple_rarefactions.py -m $MIN -x $MAX -s $STEP -n 10 -i
$SIM/40_otu_tables/rdp_method/seqs_otu_table.biom -o $SIM/40_otu_tables/rdp_method/rarefied/
#	In this work we used the following:
	multiple_rarefactions.py -m 200 -x 800 -s 200 -n 10 -i
$SIM/40_otu_tables/rdp_method/seqs_otu_table.biom -o $SIM/40_otu_tables/rdp_method/rarefied/
	if [ $i -eq 97 ]
	then
		echo -e "\t\tStep 4.8 - Summarize taxa and make plots"
		for j in {relative,absolute}
		do
			echo -e "\t\t\t$j"
			[[ "$j" == "absolute" ]] && IS_ABSOLUTE="-a" || IS_ABSOLUTE=""
			echo -e "\t\t\t\tIS_ABSOLUTE: ${IS_ABSOLUTE}"
			echo -e "\t\t\t\tStep 582 - Summarize taxa"
			summarize_taxa.py -i $SIM/40_otu_tables/rdp_method/seqs_otu_table.biom -o
$SIM/40_otu_tables/rdp_method/summarized_plots_${j}/ $IS_ABSOLUTE
			echo -e "\t\t\t\tStep 584 - Plot taxa summary"
			plot_taxa_summary.py -i
$SIM/40_otu_tables/rdp_method/summarized_plots_${j}/seqs_otu_table_L2.txt,$SIM/40_otu_tables/rdp_meth
od/summarized_plots_${j}/seqs_otu_table_L3.txt,$SIM/40_otu_tables/rdp_method/summarized_plots_${j}/se
qs_otu_table_L4.txt,$SIM/40_otu_tables/rdp_method/summarized_plots_${j}/seqs_otu_table_L5.txt,$SIM/40
_otu_tables/rdp_method/summarized_plots_${j}/seqs_otu_table_L6.txt -o
$SIM/40_otu_tables/rdp_method/summarized_plots_${j}/taxa_summary_plots/ $ONE_SAMPLE
		done
	fi
done

# ### Calculate alpha diversity metrics
echo -e "Step 5 - Calculate alpha diversity metrics"
for i in $LEVELS
```

```
do
	echo -e "\tSIMILARITY: $i"
	SIM=${i}_percent_similarity
	echo -e "\t\tStep 5.1 - Alpha diversity on rarefied otu table"
	alpha_diversity.py -t
$SIM/20_make_phylogeny/pynast_fasttree_method/seqs_rep_set_aligned_pfiltered.tre -m
chao1,dominance,equitability,observed_species,shannon,PD_whole_tree -i
$SIM/40_otu_tables/rdp_method/rarefied/ -o $SIM/50_alpha_diversity/10_on_rarefied_otu_table/
	echo -e "\t\tStep 5.2 - Collate data"
	collate_alpha.py -i $SIM/50_alpha_diversity/10_on_rarefied_otu_table/ -o
$SIM/50_alpha_diversity/20_collate_data/
	echo -e "\t\tStep 5.3 - Make rarefaction plots"
	make_rarefaction_plots.py -d 50 -g png -k white -m mapping_file.txt -i
$SIM/50_alpha_diversity/20_collate_data/ -o $SIM/50_alpha_diversity/30_rarefaction_plots/
done

# ### Calculate beta diversity metrics if there are more than 5 samples in the dataset
if [ $NUM_SAMPLES -gt 5 ]
then
	echo -e "Step 6 - Calculate beta diversity metrics"
#	SUBSET=$(($MIN*0.75)) For large and evenly distributed datasets we would use 75% of the
sequences in the smaller sample
#	In this work we used 400:
	SUBSET=400
	echo -e "\tSUBSET: $SUBSET"
	for i in $LEVELS
	do
		echo -e "\tSIMILARITY: $i"
		SIM=${i}_percent_similarity
		mkdir -p $SIM/60_beta_diversity/
		echo -e "\t\tStep 72 - Jackknifed beta diversity"
		jackknifed_beta_diversity.py -e $SUBSET -m mapping_file.txt -f -p
../custom_parameters.txt -t
$SIM/20_make_phylogeny/pynast_fasttree_method/seqs_rep_set_aligned_pfiltered.tre -i
$SIM/40_otu_tables/rdp_method/seqs_otu_table.biom -o $SIM/60_beta_diversity/
	done
fi
```

## custom_parameters.txt

```
# qiime_parameters.txt
# WARNING: DO NOT EDIT OR DELETE Qiime/qiime_parameters.txt. Users should copy this file and edit
copies of it.
# split libraries parameters
split_libraries:min-seq-length	180
split_libraries:max-seq-length	400
split_libraries:trim-seq-length
split_libraries:min-qual-score
split_libraries:keep-primer
split_libraries:keep-barcode
split_libraries:max-ambig
split_libraries:max-homopolymer	6
split_libraries:max-primer-mismatch	4
split_libraries:barcode-type	29
split_libraries:max-barcode-errors	3
split_libraries:start-numbering-at
split_libraries:remove_unassigned True
split_libraries:disable_bc_correction
split_libraries:qual_score_window 50
split_libraries:discard_bad_windows
split_libraries:disable_primers
split_libraries:reverse_primers	truncate_only
split_libraries:record_qual_scores

# OTU picker parameters
pick_otus:otu_picking_method	uclust
pick_otus:clustering_algorithm	furthest
pick_otus:max_cdhit_memory	400
pick_otus:refseqs_fp
pick_otus:blast_db
pick_otus:similarity	0.97
pick_otus:max_e_value	1e-10
pick_otus:prefix_prefilter_length
pick_otus:trie_prefilter
pick_otus:prefix_length
pick_otus:suffix_length
pick_otus:optimal_uclust
pick_otus:exact_uclust
pick_otus:user_sort
pick_otus:suppress_presort_by_abundance_uclust
pick_otus:suppress_new_clusters
pick_otus:suppress_uclust_stable_sort
pick_otus:max_accepts
pick_otus:max_rejects
pick_otus:word_length
pick_otus:stepwords
pick_otus:suppress_uclust_prefilter_exact_match

# Parallel options
parallel:jobs_to_start	1
parallel:retain_temp_files	False
parallel:seconds_to_sleep 60

# Representative set picker parameters
pick_rep_set:rep_set_picking_method	first
pick_rep_set:sort_by	otu
```

```
# Multiple sequence alignment parameters
align_seqs:template_fp
align_seqs:alignment_method	pynast
align_seqs:pairwise_alignment_method	uclust
align_seqs:blast_db
align_seqs:min_length	150
align_seqs:min_percent_id	75.0

# Alignment filtering (prior to tree-building) parameters
filter_alignment:lane_mask_fp
filter_alignment:allowed_gap_frac	0.999999
filter_alignment:remove_outliers	False
filter_alignment:threshold	3.0

# Taxonomy assignment parameters
assign_taxonomy:id_to_taxonomy_fp
assign_taxonomy:reference_seqs_fp
assign_taxonomy:assignment_method	rdp
assign_taxonomy:blast_db
assign_taxonomy:confidence	0.8
assign_taxonomy:e_value	0.001

# Phylogenetic tree building parameters
make_phylogeny:tree_method	fasttree
make_phylogeny:root_method	tree_method_default

# Beta diversity parameters
beta_diversity:metrics	weighted_unifrac,unweighted_unifrac

# Make 3D plot parameters
make_3d_plots:custom_axes
make_3d_plots:ellipsoid_smoothness	1

# Rarefaction parameters
multiple_rarefactions:num-reps	10
multiple_rarefactions:depth
multiple_rarefactions:lineages_included	False

# Even-depth rarefaction parameters
multiple_rarefactions_even_depth:num_reps	100

# Alpha diversity parameters
alpha_diversity:metrics	PD_whole_tree,chao1,observed_species

# Make rarefaction plots parameters
make_rarefaction_plots:imagetype	png
make_rarefaction_plots:resolution	75
make_rarefaction_plots:background_color	white
make_rarefaction_plots:prefs_path

# Collate alpha
collate_alpha:example_path

# OTU category significance
otu_category_significance:test
otu_category_significance:filter	3
otu_category_significance:threshold
otu_category_significance:otu_include_fp
```

## histograms.awk

```
# Bin data for a histogram
# Modifying Tara Murphy's code
# usage: for data values between 0 and 10, with steps of 0.5
# gawk -f histbin.awk 0 10 0.5 data.out data.in

BEGIN {
  m = ARGV[1]       # minimum value of x axis
  ARGV[1] = ""
  M = ARGV[2]       # maximum value of x axis
  ARGV[2] = ""
  w = ARGV[3]       # width of each step
  ARGV[3] = ""
  file = ARGV[4]    # output file for plotting
  ARGV[4] = ""

  b = (M-m)/w       # number of steps

  print "number of steps = "b
  print "width of each step  = "w
  print

  # set up arrays
  for (i = 1 ; i <= b; ++i) {
    n[i] = m+(i*w)        # upper bound of bin
    c[i] = n[i] - (w)   # centre of bin
    f[i] = 0              # frequency count
  }
}

{
  # bins the data
  for (i = 1; i <= b; ++i)
    if ($1 <= n[i]) {
         ++f[i]
         break
    }
}

END {
  # print results to screen
  # and to a file for plotting
  print "bin(centre) = freq"

  for (i = 1; i <= b; ++i) {
    if (f[i] > 0) {
      print "bin("c[i]")", "=", f[i]
      print c[i]"\t"f[i] > file
    }
    else {
      print "bin("c[i]")", "=", 0
      print c[i]"\t"0 > file
    }
  }
}
```

**<u>QIIME Mapping File in comma separated values (mapping_file.csv):</u>**

*(A “>” character was added in between lines to facilitate copy and paste from the online PDF document. In brief: copy and paste the following text in a text editor, eliminate spaces and change of line, convert “>” character into change of line. Optionally, comma can be converted into TAB)*

```
#SampleID,BarcodeSequence,LinkerPrimerSequence,ReversePrimer,RunPool,SRAAccession,UsedSamples,Site,Cr
op,PlotID,Stage,Source,454xMIDxID,NumericID,SequencedAfterQC,Folder10,Folder11,Folder12,Folder13,Fold
er14,Folder15,Folder16,Folder17,Description
>
BA2.0.Su.T.31,AGCGTCGTCT,CACGACGTTGTAAAACGACGTGCCAGCMGCCGCGGTAA,CAGGAAACAGCTATGACCGGACTACVSGGGTATCTAA
T,run1pool1,SRR3330055,Used,Balcarce,Wheat,2,Tillering,Rhizospheric_Soil,MID31,31,1326,Non,Non,Non,No
n,Non,15_wheat_RS,Non,Non,BA2.0.Su.T.31
>
BA2.1.Su.T.A.1,ACGAGTGCGT,CACGACGTTGTAAAACGACGTGCCAGCMGCCGCGGTAA,CAGGAAACAGCTATGACCGGACTACVSGGGTATCTA
AT,run1pool1,SRR3330055,NotUsed,Balcarce,Wheat,2,Tillering,Rhizospheric_Soil,MID1,1,953,Non,Non,Non,N
on,Non,Non,Non,Non,BA2.1.Su.T.A.1
>
BA3.0.Su.T.2,ACGCTCGACA,CACGACGTTGTAAAACGACGTGCCAGCMGCCGCGGTAA,CAGGAAACAGCTATGACCGGACTACVSGGGTATCTAAT
,run1pool1,SRR3330055,Used,Balcarce,Wheat,3,Tillering,Rhizospheric_Soil,MID2,2,984,Non,Non,Non,Non,No
n,15_wheat_RS,Non,Non,BA3.0.Su.T.2
>
BA3.1.Su.T.3,AGACGCACTC,CACGACGTTGTAAAACGACGTGCCAGCMGCCGCGGTAA,CAGGAAACAGCTATGACCGGACTACVSGGGTATCTAAT
,run1pool1,SRR3330055,Used,Balcarce,Wheat,3,Tillering,Rhizospheric_Soil,MID3,3,1995,Non,11_wheat_RA_R
S,Non,Non,Non,15_wheat_RS,16_wheat_soybean_RA_RS,Non,BA3.1.Su.T.3
>
BA4.0.Su.T.4,AGCACTGTAG,CACGACGTTGTAAAACGACGTGCCAGCMGCCGCGGTAA,CAGGAAACAGCTATGACCGGACTACVSGGGTATCTAAT
,run1pool1,SRR3330055,Used,Balcarce,Wheat,4,Tillering,Rhizospheric_Soil,MID4,4,1899,Non,11_wheat_RA_R
S,Non,Non,Non,15_wheat_RS,16_wheat_soybean_RA_RS,Non,BA4.0.Su.T.4
>
BA4.1.Su.T.5,ATCAGACACG,CACGACGTTGTAAAACGACGTGCCAGCMGCCGCGGTAA,CAGGAAACAGCTATGACCGGACTACVSGGGTATCTAAT
,run1pool1,SRR3330055,Used,Balcarce,Wheat,4,Tillering,Rhizospheric_Soil,MID5,5,1995,Non,11_wheat_RA_R
S,Non,Non,Non,15_wheat_RS,16_wheat_soybean_RA_RS,Non,BA4.1.Su.T.5
>
BA5.1.0.Su.T.6,ATATCGCGAG,CACGACGTTGTAAAACGACGTGCCAGCMGCCGCGGTAA,CAGGAAACAGCTATGACCGGACTACVSGGGTATCTA
AT,run1pool1,SRR3330055,NotUsed,Balcarce,Wheat,5,Tillering,Rhizospheric_Soil,MID6,6,1813,Non,Non,Non,
Non,Non,Non,Non,Non,BA5.1.0.Su.T.6
>
BA5.1.1.Su.T.7,CGTGTCTCTA,CACGACGTTGTAAAACGACGTGCCAGCMGCCGCGGTAA,CAGGAAACAGCTATGACCGGACTACVSGGGTATCTA
AT,run1pool1,SRR3330055,Used,Balcarce,Wheat,5,Tillering,Rhizospheric_Soil,MID7,7,1733,Non,11_wheat_RA
_RS,Non,Non,Non,15_wheat_RS,16_wheat_soybean_RA_RS,Non,BA5.1.1.Su.T.7
>
BA5.2.1.Su.T.8,CTCGCGTGTC,CACGACGTTGTAAAACGACGTGCCAGCMGCCGCGGTAA,CAGGAAACAGCTATGACCGGACTACVSGGGTATCTA
AT,run1pool1,SRR3330055,NotUsed,Balcarce,Wheat,5,Tillering,Rhizospheric_Soil,MID8,8,1733,Non,Non,Non,
Non,Non,Non,Non,Non,BA5.2.1.Su.T.8
>
BA5.3.0.Su.T.10,TCTCTATGCG,CACGACGTTGTAAAACGACGTGCCAGCMGCCGCGGTAA,CAGGAAACAGCTATGACCGGACTACVSGGGTATCT
AAT,run1pool1,SRR3330055,NotUsed,Balcarce,Wheat,5,Tillering,Rhizospheric_Soil,MID10,10,1204,Non,Non,N
on,Non,Non,Non,Non,Non,BA5.3.0.Su.T.10
>
BA5.3.1.Su.T.11,TGATACGTCT,CACGACGTTGTAAAACGACGTGCCAGCMGCCGCGGTAA,CAGGAAACAGCTATGACCGGACTACVSGGGTATCT
AAT,run1pool1,SRR3330055,NotUsed,Balcarce,Wheat,5,Tillering,Rhizospheric_Soil,MID11,11,524,Non,Non,No
n,Non,Non,Non,Non,Non,BA5.3.1.Su.T.11
>
```

BA5.2.0.Su.T.33,ATAGAGTACT,CACGACGTTGTAAAACGACGTGCCAGCMGCCGCGGTAA,CAGGAAACAGCTATGACCGGACTACVSGGGTATCT
AAT,run1pool1,SRR3330055,Used,Balcarce,Wheat,5,Tillering,Rhizospheric_Soil,MID33,33,3425,Non,11_wheat
_RA_RS,Non,Non,Non,15_wheat_RS,16_wheat_soybean_RA_RS,Non,BA5.2.0.Su.T.33
>
BA2.0.Su.TF.36,CGACGTGACT,CACGACGTTGTAAAACGACGTGCCAGCMGCCGCGGTAA,CAGGAAACAGCTATGACCGGACTACVSGGGTATCTA
AT,run1pool1,SRR3330055,NotUsed,Balcarce,Wheat,2,Flowering,Rhizospheric_Soil,MID36,36,1745,Non,Non,No
n,Non,Non,Non,Non,Non,BA2.0.Su.TF.36
>
BA2.1.Su.TF.13,CATAGTAGTG,CACGACGTTGTAAAACGACGTGCCAGCMGCCGCGGTAA,CAGGAAACAGCTATGACCGGACTACVSGGGTATCTA
AT,run1pool1,SRR3330055,NotUsed,Balcarce,Wheat,2,Flowering,Rhizospheric_Soil,MID13,13,811,Non,Non,Non
,Non,Non,Non,Non,Non,BA2.1.Su.TF.13
>
BA3.0.Su.TF.14,CGAGAGATAC,CACGACGTTGTAAAACGACGTGCCAGCMGCCGCGGTAA,CAGGAAACAGCTATGACCGGACTACVSGGGTATCTA
AT,run1pool1,SRR3330055,NotUsed,Balcarce,Wheat,3,Flowering,Rhizospheric_Soil,MID14,14,1540,Non,Non,No
n,Non,Non,Non,Non,Non,BA3.0.Su.TF.14
>
BA3.1.Su.TF.15,ATACGACGTA,CACGACGTTGTAAAACGACGTGCCAGCMGCCGCGGTAA,CAGGAAACAGCTATGACCGGACTACVSGGGTATCTA
AT,run1pool1,SRR3330055,NotUsed,Balcarce,Wheat,3,Flowering,Rhizospheric_Soil,MID15,15,1586,Non,Non,No
n,Non,Non,Non,Non,Non,BA3.1.Su.TF.15
>
BA4.0.Su.TF.16,TCACGTACTA,CACGACGTTGTAAAACGACGTGCCAGCMGCCGCGGTAA,CAGGAAACAGCTATGACCGGACTACVSGGGTATCTA
AT,run1pool1,SRR3330055,NotUsed,Balcarce,Wheat,4,Flowering,Rhizospheric_Soil,MID16,16,1863,Non,Non,No
n,Non,Non,Non,Non,Non,BA4.0.Su.TF.16
>
BA4.1.Su.TF.17,CGTCTAGTAC,CACGACGTTGTAAAACGACGTGCCAGCMGCCGCGGTAA,CAGGAAACAGCTATGACCGGACTACVSGGGTATCTA
AT,run1pool1,SRR3330055,NotUsed,Balcarce,Wheat,4,Flowering,Rhizospheric_Soil,MID17,17,1405,Non,Non,No
n,Non,Non,Non,Non,Non,BA4.1.Su.TF.17
>
BA5.1.0.Su.TF.18,TCTACGTAGC,CACGACGTTGTAAAACGACGTGCCAGCMGCCGCGGTAA,CAGGAAACAGCTATGACCGGACTACVSGGGTATC
TAAT,run1pool1,SRR3330055,NotUsed,Balcarce,Wheat,5,Flowering,Rhizospheric_Soil,MID18,18,1561,Non,Non,
Non,Non,Non,Non,Non,Non,BA5.1.0.Su.TF.18
>
BA5.1.1.Su.TF.19,TGTACTACTC,CACGACGTTGTAAAACGACGTGCCAGCMGCCGCGGTAA,CAGGAAACAGCTATGACCGGACTACVSGGGTATC
TAAT,run1pool1,SRR3330055,NotUsed,Balcarce,Wheat,5,Flowering,Rhizospheric_Soil,MID19,19,879,Non,Non,N
on,Non,Non,Non,Non,Non,BA5.1.1.Su.TF.19
>
BA5.2.0.Su.TF.20,ACGACTACAG,CACGACGTTGTAAAACGACGTGCCAGCMGCCGCGGTAA,CAGGAAACAGCTATGACCGGACTACVSGGGTATC
TAAT,run1pool1,SRR3330055,NotUsed,Balcarce,Wheat,5,Flowering,Rhizospheric_Soil,MID20,20,1261,Non,Non,
Non,Non,Non,Non,Non,Non,BA5.2.0.Su.TF.20
>
BA5.2.1.Su.TF.21,CGTAGACTAG,CACGACGTTGTAAAACGACGTGCCAGCMGCCGCGGTAA,CAGGAAACAGCTATGACCGGACTACVSGGGTATC
TAAT,run1pool1,SRR3330055,NotUsed,Balcarce,Wheat,5,Flowering,Rhizospheric_Soil,MID21,21,1524,Non,Non,
Non,Non,Non,Non,Non,Non,BA5.2.1.Su.TF.21
>
BA5.3.0.Su.TF.22,TACGAGTATG,CACGACGTTGTAAAACGACGTGCCAGCMGCCGCGGTAA,CAGGAAACAGCTATGACCGGACTACVSGGGTATC
TAAT,run1pool1,SRR3330055,NotUsed,Balcarce,Wheat,5,Flowering,Rhizospheric_Soil,MID22,22,1393,Non,Non,
Non,Non,Non,Non,Non,Non,BA5.3.0.Su.TF.22
>
BA5.3.1.Su.TF.23,TACTCTCGTG,CACGACGTTGTAAAACGACGTGCCAGCMGCCGCGGTAA,CAGGAAACAGCTATGACCGGACTACVSGGGTATC
TAAT,run1pool1,SRR3330055,NotUsed,Balcarce,Wheat,5,Flowering,Rhizospheric_Soil,MID23,23,1758,Non,Non,
Non,Non,Non,Non,Non,Non,BA5.3.1.Su.TF.23
>
MB2.0.Su.T.A.38,TACACGTGAT,CACGACGTTGTAAAACGACGTGCCAGCMGCCGCGGTAA,CAGGAAACAGCTATGACCGGACTACVSGGGTATCT
AAT,run1pool1,SRR3330055,Used,MonteBuey,Wheat,2,Tillering,Rhizospheric_Soil,MID38,38,1586,Non,Non,Non
,Non,Non,15_wheat_RS,Non,Non,MB2.0.Su.T.A.38
>
MB2.1.Su.T.24,TAGAGACGAG,CACGACGTTGTAAAACGACGTGCCAGCMGCCGCGGTAA,CAGGAAACAGCTATGACCGGACTACVSGGGTATCTAA
T,run1pool1,SRR3330055,Used,MonteBuey,Wheat,2,Tillering,Rhizospheric_Soil,MID24,24,1415,Non,Non,Non,N
on,Non,15_wheat_RS,Non,Non,MB2.1.Su.T.24

>
MB3.0.Su.T.25,TCGTCGCTCG,CACGACGTTGTAAAACGACGTGCCAGCMGCCGCGGTAA,CAGGAAACAGCTATGACCGGACTACVSGGGTATCTAA
T,run1pool1,SRR3330055,Used,MonteBuey,Wheat,3,Tillering,Rhizospheric_Soil,MID25,25,1447,Non,Non,Non,N
on,Non,15_wheat_RS,Non,Non,MB3.0.Su.T.25
>
MB3.1.Su.T.26,ACATACGCGT,CACGACGTTGTAAAACGACGTGCCAGCMGCCGCGGTAA,CAGGAAACAGCTATGACCGGACTACVSGGGTATCTAA
T,run1pool1,SRR3330055,Used,MonteBuey,Wheat,3,Tillering,Rhizospheric_Soil,MID26,26,1352,Non,Non,Non,N
on,Non,15_wheat_RS,Non,Non,MB3.1.Su.T.26
>
MB4.1.Su.T.28,ACTACTATGT,CACGACGTTGTAAAACGACGTGCCAGCMGCCGCGGTAA,CAGGAAACAGCTATGACCGGACTACVSGGGTATCTAA
T,run1pool1,SRR3330055,Used,MonteBuey,Wheat,4,Tillering,Rhizospheric_Soil,MID28,28,1042,Non,Non,Non,N
on,Non,15_wheat_RS,Non,Non,MB4.1.Su.T.28
>
MB5.0.Su.T.29,ACTGTACAGT,CACGACGTTGTAAAACGACGTGCCAGCMGCCGCGGTAA,CAGGAAACAGCTATGACCGGACTACVSGGGTATCTAA
T,run1pool1,SRR3330055,Used,MonteBuey,Wheat,5,Tillering,Rhizospheric_Soil,MID29,29,722,Non,Non,Non,No
n,Non,15_wheat_RS,Non,Non,MB5.0.Su.T.29
>
MB5.1.Su.T.30,AGACTATACT,CACGACGTTGTAAAACGACGTGCCAGCMGCCGCGGTAA,CAGGAAACAGCTATGACCGGACTACVSGGGTATCTAA
T,run1pool1,SRR3330055,Used,MonteBuey,Wheat,5,Tillering,Rhizospheric_Soil,MID30,30,6311,Non,Non,Non,N
on,Non,15_wheat_RS,Non,Non,MB5.1.Su.T.30
>
VS2.0.Su.T.A.37,TACACACACT,CACGACGTTGTAAAACGACGTGCCAGCMGCCGCGGTAA,CAGGAAACAGCTATGACCGGACTACVSGGGTATCT
AAT,run1pool1,SRR3330055,Used,VillaSaboya,Wheat,2,Tillering,Rhizospheric_Soil,MID37,37,1415,Non,Non,N
on,Non,Non,15_wheat_RS,Non,Non,VS2.0.Su.T.A.37
>
VS2.1.Su.T.40,TACGCTGTCT,CACGACGTTGTAAAACGACGTGCCAGCMGCCGCGGTAA,CAGGAAACAGCTATGACCGGACTACVSGGGTATCTAA
T,run1pool1,SRR3330055,Used,VillaSaboya,Wheat,2,Tillering,Rhizospheric_Soil,MID40,40,1184,Non,11_whea
t_RA_RS,Non,Non,Non,15_wheat_RS,16_wheat_soybean_RA_RS,Non,VS2.1.Su.T.40
>
VS3.0.Su.T.41,TAGTGTAGAT,CACGACGTTGTAAAACGACGTGCCAGCMGCCGCGGTAA,CAGGAAACAGCTATGACCGGACTACVSGGGTATCTAA
T,run1pool1,SRR3330055,Used,VillaSaboya,Wheat,3,Tillering,Rhizospheric_Soil,MID41,41,1469,Non,11_whea
t_RA_RS,Non,Non,Non,15_wheat_RS,16_wheat_soybean_RA_RS,Non,VS3.0.Su.T.41
>
VS3.1.Su.T.42,TCGATCACGT,CACGACGTTGTAAAACGACGTGCCAGCMGCCGCGGTAA,CAGGAAACAGCTATGACCGGACTACVSGGGTATCTAA
T,run1pool1,SRR3330055,Used,VillaSaboya,Wheat,3,Tillering,Rhizospheric_Soil,MID42,42,1429,Non,11_whea
t_RA_RS,Non,Non,Non,15_wheat_RS,16_wheat_soybean_RA_RS,Non,VS3.1.Su.T.42
>
VS4.1.Su.T.44,TCTAGCGACT,CACGACGTTGTAAAACGACGTGCCAGCMGCCGCGGTAA,CAGGAAACAGCTATGACCGGACTACVSGGGTATCTAA
T,run1pool1,SRR3330055,NotUsed,VillaSaboya,Wheat,4,Tillering,Rhizospheric_Soil,MID44,44,583,Non,Non,N
on,Non,Non,Non,Non,Non,VS4.1.Su.T.44
>
VS5.0.Su.T.45,TCTATACTAT,CACGACGTTGTAAAACGACGTGCCAGCMGCCGCGGTAA,CAGGAAACAGCTATGACCGGACTACVSGGGTATCTAA
T,run1pool1,SRR3330055,Used,VillaSaboya,Wheat,5,Tillering,Rhizospheric_Soil,MID45,45,812,Non,Non,Non,
Non,Non,15_wheat_RS,Non,Non,VS5.0.Su.T.45
>
VS5.1.Su.T.46,TGACGTATGT,CACGACGTTGTAAAACGACGTGCCAGCMGCCGCGGTAA,CAGGAAACAGCTATGACCGGACTACVSGGGTATCTAA
T,run1pool1,SRR3330055,Used,VillaSaboya,Wheat,5,Tillering,Rhizospheric_Soil,MID46,46,1637,Non,11_whea
t_RA_RS,Non,Non,Non,15_wheat_RS,16_wheat_soybean_RA_RS,Non,VS5.1.Su.T.46
>
CB2.0.Su.T.A.32,AGTACGCTAT,CACGACGTTGTAAAACGACGTGCCAGCMGCCGCGGTAA,CAGGAAACAGCTATGACCGGACTACVSGGGTATCT
AAT,run1pool1,SRR3330055,Used,CorraldeBustos,Wheat,2,Tillering,Rhizospheric_Soil,MID32,32,1563,Non,11
_wheat_RA_RS,Non,Non,Non,15_wheat_RS,16_wheat_soybean_RA_RS,Non,CB2.0.Su.T.A.32
>
CB3.0.Su.T.47,TGTGAGTAGT,CACGACGTTGTAAAACGACGTGCCAGCMGCCGCGGTAA,CAGGAAACAGCTATGACCGGACTACVSGGGTATCTAA
T,run1pool1,SRR3330055,Used,CorraldeBustos,Wheat,3,Tillering,Rhizospheric_Soil,MID47,47,1028,Non,Non,
Non,Non,Non,15_wheat_RS,Non,Non,CB3.0.Su.T.47
>

CB4.0.Su.T.48,ACAGTATATA,CACGACGTTGTAAAACGACGTGCCAGCMGCCGCGGTAA,CAGGAAACAGCTATGACCGGACTACVSGGGTATCTAAT,run1pool1,SRR3330055,Used,CorraldeBustos,Wheat,4,Tillering,Rhizospheric_Soil,MID48,48,1314,Non,11_wheat_RA_RS,Non,Non,Non,15_wheat_RS,16_wheat_soybean_RA_RS,Non,CB4.0.Su.T.48
>
CB5.0.Su.T.49,ACGCGATCGA,CACGACGTTGTAAAACGACGTGCCAGCMGCCGCGGTAA,CAGGAAACAGCTATGACCGGACTACVSGGGTATCTAAT,run1pool1,SRR3330055,Used,CorraldeBustos,Wheat,5,Tillering,Rhizospheric_Soil,MID49,49,773,Non,11_wheat_RA_RS,Non,Non,Non,15_wheat_RS,16_wheat_soybean_RA_RS,Non,CB5.0.Su.T.49
>
CB2.1.Su.T.50,ACTAGCAGTA,CACGACGTTGTAAAACGACGTGCCAGCMGCCGCGGTAA,CAGGAAACAGCTATGACCGGACTACVSGGGTATCTAAT,run1pool1,SRR3330055,Used,CorraldeBustos,Wheat,2,Tillering,Rhizospheric_Soil,MID50,50,1837,Non,11_wheat_RA_RS,Non,Non,Non,15_wheat_RS,16_wheat_soybean_RA_RS,Non,CB2.1.Su.T.50
>
CB3.1.Su.T.51,AGCTCACGTA,CACGACGTTGTAAAACGACGTGCCAGCMGCCGCGGTAA,CAGGAAACAGCTATGACCGGACTACVSGGGTATCTAAT,run1pool1,SRR3330055,Used,CorraldeBustos,Wheat,3,Tillering,Rhizospheric_Soil,MID51,51,1296,Non,11_wheat_RA_RS,Non,Non,Non,15_wheat_RS,16_wheat_soybean_RA_RS,Non,CB3.1.Su.T.51
>
CB4.1.Su.T.52,AGTATACATA,CACGACGTTGTAAAACGACGTGCCAGCMGCCGCGGTAA,CAGGAAACAGCTATGACCGGACTACVSGGGTATCTAAT,run1pool1,SRR3330055,Used,CorraldeBustos,Wheat,4,Tillering,Rhizospheric_Soil,MID52,52,1053,Non,11_wheat_RA_RS,Non,Non,Non,15_wheat_RS,16_wheat_soybean_RA_RS,Non,CB4.1.Su.T.52
>
CB5.1.Su.T.53,AGTCGAGAGA,CACGACGTTGTAAAACGACGTGCCAGCMGCCGCGGTAA,CAGGAAACAGCTATGACCGGACTACVSGGGTATCTAAT,run1pool1,SRR3330055,NotUsed,CorraldeBustos,Wheat,5,Tillering,Rhizospheric_Soil,MID53,53,666,Non,Non,Non,Non,Non,Non,Non,Non,CB5.1.Su.T.53
>
SJ5.1.1.Su.T.A.35,CAGTAGACGT,CACGACGTTGTAAAACGACGTGCCAGCMGCCGCGGTAA,CAGGAAACAGCTATGACCGGACTACVSGGGTATCTAAT,run1pool1,SRR3330055,NotUsed,SanJorge,Wheat,5,Tillering,Rhizospheric_Soil,MID35,35,1316,Non,Non,Non,Non,Non,Non,Non,Non,SJ5.1.1.Su.T.A.35
>
SJ2.0.Su.T.A.54,AGTGCTACGA,CACGACGTTGTAAAACGACGTGCCAGCMGCCGCGGTAA,CAGGAAACAGCTATGACCGGACTACVSGGGTATCTAAT,run1pool1,SRR3330055,Used,SanJorge,Wheat,2,Tillering,Rhizospheric_Soil,MID54,54,1614,Non,Non,Non,Non,Non,15_wheat_RS,Non,Non,SJ2.0.Su.T.A.54
>
SJ3.0.Su.T.55,CGATCGTATA,CACGACGTTGTAAAACGACGTGCCAGCMGCCGCGGTAA,CAGGAAACAGCTATGACCGGACTACVSGGGTATCTAAT,run1pool1,SRR3330055,Used,SanJorge,Wheat,3,Tillering,Rhizospheric_Soil,MID55,55,1277,Non,Non,Non,Non,Non,15_wheat_RS,Non,Non,SJ3.0.Su.T.55
>
SJ4.0.Su.T.56,CGCAGTACGA,CACGACGTTGTAAAACGACGTGCCAGCMGCCGCGGTAA,CAGGAAACAGCTATGACCGGACTACVSGGGTATCTAAT,run1pool1,SRR3330055,Used,SanJorge,Wheat,4,Tillering,Rhizospheric_Soil,MID56,56,1044,Non,Non,Non,Non,Non,15_wheat_RS,Non,Non,SJ4.0.Su.T.56
>
SJ5.2.0.Su.T.A.57,CGCGTATACA,CACGACGTTGTAAAACGACGTGCCAGCMGCCGCGGTAA,CAGGAAACAGCTATGACCGGACTACVSGGGTATCTAAT,run1pool1,SRR3330055,NotUsed,SanJorge,Wheat,5,Tillering,Rhizospheric_Soil,MID57,57,935,Non,Non,Non,Non,Non,Non,Non,Non,SJ5.2.0.Su.T.A.57
>
SJ2.1.Su.T.58,CGTACAGTCA,CACGACGTTGTAAAACGACGTGCCAGCMGCCGCGGTAA,CAGGAAACAGCTATGACCGGACTACVSGGGTATCTAAT,run1pool1,SRR3330055,Used,SanJorge,Wheat,2,Tillering,Rhizospheric_Soil,MID58,58,1460,Non,Non,Non,Non,Non,15_wheat_RS,Non,Non,SJ2.1.Su.T.58
>
SJ3.1.Su.T.59,CGTACTCAGA,CACGACGTTGTAAAACGACGTGCCAGCMGCCGCGGTAA,CAGGAAACAGCTATGACCGGACTACVSGGGTATCTAAT,run1pool1,SRR3330055,Used,SanJorge,Wheat,3,Tillering,Rhizospheric_Soil,MID59,59,1077,Non,Non,Non,Non,Non,15_wheat_RS,Non,Non,SJ3.1.Su.T.59
>
SJ4.1.Su.T.60,CTACGCTCTA,CACGACGTTGTAAAACGACGTGCCAGCMGCCGCGGTAA,CAGGAAACAGCTATGACCGGACTACVSGGGTATCTAAT,run1pool1,SRR3330055,Used,SanJorge,Wheat,4,Tillering,Rhizospheric_Soil,MID60,60,876,Non,Non,Non,Non,Non,15_wheat_RS,Non,Non,SJ4.1.Su.T.60
>
SJ5.1.0.Su.T.61,CTATAGCGTA,CACGACGTTGTAAAACGACGTGCCAGCMGCCGCGGTAA,CAGGAAACAGCTATGACCGGACTACVSGGGTATCTAAT,run1pool1,SRR3330055,NotUsed,SanJorge,Wheat,5,Tillering,Rhizospheric_Soil,MID61,61,1034,Non,Non,Non,Non,Non,Non,Non,Non,SJ5.1.0.Su.T.61

>
SJ5.2.1.Su.T.62,TACGTCATCA,CACGACGTTGTAAAACGACGTGCCAGCMGCCGCGGTAA,CAGGAAACAGCTATGACCGGACTACVSGGGTATCTAAT,run1pool1,SRR3330055,Used,SanJorge,Wheat,5,Tillering,Rhizospheric_Soil,MID62,62,844,Non,Non,Non,Non,Non,15_wheat_RS,Non,Non,SJ5.2.1.Su.T.62
>
SJ5.3.0.Su.T.63,TAGTCGCATA,CACGACGTTGTAAAACGACGTGCCAGCMGCCGCGGTAA,CAGGAAACAGCTATGACCGGACTACVSGGGTATCTAAT,run1pool1,SRR3330055,NotUsed,SanJorge,Wheat,5,Tillering,Rhizospheric_Soil,MID63,63,1147,Non,Non,Non,Non,Non,Non,Non,Non,SJ5.3.0.Su.T.63
>
SJ5.3.1.Su.T.64,TATATATACA,CACGACGTTGTAAAACGACGTGCCAGCMGCCGCGGTAA,CAGGAAACAGCTATGACCGGACTACVSGGGTATCTAAT,run1pool1,SRR3330055,NotUsed,SanJorge,Wheat,5,Tillering,Rhizospheric_Soil,MID64,64,1505,Non,Non,Non,Non,Non,Non,Non,Non,SJ5.3.1.Su.T.64
>
DA2.0.Su.T.A.34,CACGCTACGT,CACGACGTTGTAAAACGACGTGCCAGCMGCCGCGGTAA,CAGGAAACAGCTATGACCGGACTACVSGGGTATCTAAT,run1pool1,SRR3330055,Used,Daireaux,Wheat,2,Tillering,Rhizospheric_Soil,MID34,34,1081,Non,Non,Non,Non,Non,15_wheat_RS,Non,Non,DA2.0.Su.T.A.34
>
DA2.1.Su.T.65,TATGCTAGTA,CACGACGTTGTAAAACGACGTGCCAGCMGCCGCGGTAA,CAGGAAACAGCTATGACCGGACTACVSGGGTATCTAAT,run1pool1,SRR3330055,Used,Daireaux,Wheat,2,Tillering,Rhizospheric_Soil,MID65,65,1444,Non,11_wheat_RA_RS,Non,Non,Non,15_wheat_RS,16_wheat_soybean_RA_RS,Non,DA2.1.Su.T.65
>
DA3.0.Su.T.66,TCACGCGAGA,CACGACGTTGTAAAACGACGTGCCAGCMGCCGCGGTAA,CAGGAAACAGCTATGACCGGACTACVSGGGTATCTAAT,run1pool1,SRR3330055,Used,Daireaux,Wheat,3,Tillering,Rhizospheric_Soil,MID66,66,918,Non,Non,Non,Non,Non,15_wheat_RS,Non,Non,DA3.0.Su.T.66
>
DA3.1.Su.T.67,TCGATAGTGA,CACGACGTTGTAAAACGACGTGCCAGCMGCCGCGGTAA,CAGGAAACAGCTATGACCGGACTACVSGGGTATCTAAT,run1pool1,SRR3330055,Used,Daireaux,Wheat,3,Tillering,Rhizospheric_Soil,MID67,67,1189,Non,11_wheat_RA_RS,Non,Non,Non,15_wheat_RS,16_wheat_soybean_RA_RS,Non,DA3.1.Su.T.67
>
DA4.0.Su.T.68,TCGCTGCGTA,CACGACGTTGTAAAACGACGTGCCAGCMGCCGCGGTAA,CAGGAAACAGCTATGACCGGACTACVSGGGTATCTAAT,run1pool1,SRR3330055,Used,Daireaux,Wheat,4,Tillering,Rhizospheric_Soil,MID68,68,1325,Non,11_wheat_RA_RS,Non,Non,Non,15_wheat_RS,16_wheat_soybean_RA_RS,Non,DA4.0.Su.T.68
>
DA4.1.Su.T.69,TCTGACGTCA,CACGACGTTGTAAAACGACGTGCCAGCMGCCGCGGTAA,CAGGAAACAGCTATGACCGGACTACVSGGGTATCTAAT,run1pool1,SRR3330055,NotUsed,Daireaux,Wheat,4,Tillering,Rhizospheric_Soil,MID69,69,959,Non,Non,Non,Non,Non,Non,Non,Non,DA4.1.Su.T.69
>
DA5.0.Su.T.70,TGAGTCAGTA,CACGACGTTGTAAAACGACGTGCCAGCMGCCGCGGTAA,CAGGAAACAGCTATGACCGGACTACVSGGGTATCTAAT,run1pool1,SRR3330055,Used,Daireaux,Wheat,5,Tillering,Rhizospheric_Soil,MID70,70,1534,Non,11_wheat_RA_RS,Non,Non,Non,15_wheat_RS,16_wheat_soybean_RA_RS,Non,DA5.0.Su.T.70
>
DA5.1.Su.T.71,TGTAGTGTGA,CACGACGTTGTAAAACGACGTGCCAGCMGCCGCGGTAA,CAGGAAACAGCTATGACCGGACTACVSGGGTATCTAAT,run1pool1,SRR3330055,Used,Daireaux,Wheat,5,Tillering,Rhizospheric_Soil,MID71,71,1721,Non,11_wheat_RA_RS,Non,Non,Non,15_wheat_RS,16_wheat_soybean_RA_RS,Non,DA5.1.Su.T.71
>
AR2.0.Su.S.49,ATACGACGTA,CACGACGTTGTAAAACGACGTGCCAGCMGCCGCGGTAA,CAGGAAACAGCTATGACCGGACTACVSGGGTATCTAAT,run2pool1,SRR3330056,NotUsed,Aranguren,Soybean,2,Vegetative,Rhizospheric_Soil,MID15,49,301,Non,Non,Non,Non,Non,Non,Non,Non,AR2.0.Su.S.49
>
AR2a11.1.Su.S.50,ACGAGTGCGT,CACGACGTTGTAAAACGACGTGCCAGCMGCCGCGGTAA,CAGGAAACAGCTATGACCGGACTACVSGGGTATCTAAT,run2pool1,SRR3330056,Used,Aranguren,Soybean,2,Vegetative,Rhizospheric_Soil,MID1,50,742,10_soybean_RA_RS,Non,Non,13_soybean_RS,Non,Non,16_wheat_soybean_RA_RS,Non,AR2a11.1.Su.S.50
>
AR3.0.Su.S.51,ACGCTCGACA,CACGACGTTGTAAAACGACGTGCCAGCMGCCGCGGTAA,CAGGAAACAGCTATGACCGGACTACVSGGGTATCTAAT,run2pool1,SRR3330056,Used,Aranguren,Soybean,3,Vegetative,Rhizospheric_Soil,MID2,51,589,10_soybean_RA_RS,Non,Non,13_soybean_RS,Non,Non,16_wheat_soybean_RA_RS,Non,AR3.0.Su.S.51
>

AR3a11.1.Su.S.52,TACGAGTATG,CACGACGTTGTAAAACGACGTGCCAGCMGCCGCGGTAA,CAGGAAACAGCTATGACCGGACTACVSGGGTATCTAAT,run2pool1,SRR3330056,Used,Aranguren,Soybean,3,Vegetative,Rhizospheric_Soil,MID22,52,592,10_soybean_RA_RS,Non,Non,13_soybean_RS,Non,Non,16_wheat_soybean_RA_RS,Non,AR3a11.1.Su.S.52

>

CA2.0.Su.S.53,AGACGCACTC,CACGACGTTGTAAAACGACGTGCCAGCMGCCGCGGTAA,CAGGAAACAGCTATGACCGGACTACVSGGGTATCTAAT,run2pool1,SRR3330056,Used,CarmendeAreco,Soybean,2,Vegetative,Rhizospheric_Soil,MID3,53,660,10_soybean_RA_RS,Non,Non,13_soybean_RS,Non,Non,16_wheat_soybean_RA_RS,Non,CA2.0.Su.S.53

>

CA2a11.1.Su.S.54,AGCACTGTAG,CACGACGTTGTAAAACGACGTGCCAGCMGCCGCGGTAA,CAGGAAACAGCTATGACCGGACTACVSGGGTATCTAAT,run2pool1,SRR3330056,Used,CarmendeAreco,Soybean,2,Vegetative,Rhizospheric_Soil,MID4,54,737,10_soybean_RA_RS,Non,Non,13_soybean_RS,Non,Non,16_wheat_soybean_RA_RS,Non,CA2a11.1.Su.S.54

>

CA3.0.Su.S.55,TCACGTACTA,CACGACGTTGTAAAACGACGTGCCAGCMGCCGCGGTAA,CAGGAAACAGCTATGACCGGACTACVSGGGTATCTAAT,run2pool1,SRR3330056,NotUsed,CarmendeAreco,Soybean,3,Vegetative,Rhizospheric_Soil,MID16,55,594,Non,Non,Non,Non,Non,Non,Non,Non,CA3.0.Su.S.55

>

CA3a11.1.Su.S.56,ATCAGACACG,CACGACGTTGTAAAACGACGTGCCAGCMGCCGCGGTAA,CAGGAAACAGCTATGACCGGACTACVSGGGTATCTAAT,run2pool1,SRR3330056,Used,CarmendeAreco,Soybean,3,Vegetative,Rhizospheric_Soil,MID5,56,872,10_soybean_RA_RS,Non,Non,13_soybean_RS,Non,Non,16_wheat_soybean_RA_RS,Non,CA3a11.1.Su.S.56

>

CB2.0.Su.S.57,ATATCGCGAG,CACGACGTTGTAAAACGACGTGCCAGCMGCCGCGGTAA,CAGGAAACAGCTATGACCGGACTACVSGGGTATCTAAT,run2pool1,SRR3330056,Used,CorraldeBustos,Soybean,2,Vegetative,Rhizospheric_Soil,MID6,57,379,Non,Non,Non,Non,Non,Non,Non,Non,CB2.0.Su.S.57

>

CB2a5.1.Su.S.58,CGTGTCTCTA,CACGACGTTGTAAAACGACGTGCCAGCMGCCGCGGTAA,CAGGAAACAGCTATGACCGGACTACVSGGGTATCTAAT,run2pool1,SRR3330056,NotUsed,CorraldeBustos,Soybean,2,Vegetative,Rhizospheric_Soil,MID7,58,219,Non,Non,Non,Non,Non,Non,Non,Non,CB2a5.1.Su.S.58

>

CB3.0.Su.S.59,CTCGCGTGTC,CACGACGTTGTAAAACGACGTGCCAGCMGCCGCGGTAA,CAGGAAACAGCTATGACCGGACTACVSGGGTATCTAAT,run2pool1,SRR3330056,Used,CorraldeBustos,Soybean,3,Vegetative,Rhizospheric_Soil,MID8,59,255,Non,Non,Non,Non,Non,Non,Non,Non,CB3.0.Su.S.59

>

CB3a11.1.Su.S.60,CGTCTAGTAC,CACGACGTTGTAAAACGACGTGCCAGCMGCCGCGGTAA,CAGGAAACAGCTATGACCGGACTACVSGGGTATCTAAT,run2pool1,SRR3330056,Used,CorraldeBustos,Soybean,3,Vegetative,Rhizospheric_Soil,MID17,60,926,Non,Non,Non,Non,Non,Non,Non,Non,CB3a11.1.Su.S.60

>

CH2.0.Su.S.61,CGACACTATC,CACGACGTTGTAAAACGACGTGCCAGCMGCCGCGGTAA,CAGGAAACAGCTATGACCGGACTACVSGGGTATCTAAT,run2pool1,SRR3330056,Used,Chilibroste,Soybean,2,Vegetative,Rhizospheric_Soil,MID92,61,933,10_soybean_RA_RS,Non,Non,13_soybean_RS,Non,Non,16_wheat_soybean_RA_RS,Non,CH2.0.Su.S.61

>

CH2a11.1.Su.S.62,TACTCTCGTG,CACGACGTTGTAAAACGACGTGCCAGCMGCCGCGGTAA,CAGGAAACAGCTATGACCGGACTACVSGGGTATCTAAT,run2pool1,SRR3330056,Used,Chilibroste,Soybean,2,Vegetative,Rhizospheric_Soil,MID23,62,624,10_soybean_RA_RS,Non,Non,13_soybean_RS,Non,Non,16_wheat_soybean_RA_RS,Non,CH2a11.1.Su.S.62

>

CH3.0.Su.S.63,TCTCTATGCG,CACGACGTTGTAAAACGACGTGCCAGCMGCCGCGGTAA,CAGGAAACAGCTATGACCGGACTACVSGGGTATCTAAT,run2pool1,SRR3330056,Used,Chilibroste,Soybean,3,Vegetative,Rhizospheric_Soil,MID10,63,860,10_soybean_RA_RS,Non,Non,13_soybean_RS,Non,Non,16_wheat_soybean_RA_RS,Non,CH3.0.Su.S.63

>

CH3a11.1.Su.S.64,TAGAGACGAG,CACGACGTTGTAAAACGACGTGCCAGCMGCCGCGGTAA,CAGGAAACAGCTATGACCGGACTACVSGGGTATCTAAT,run2pool1,SRR3330056,NotUsed,Chilibroste,Soybean,3,Vegetative,Rhizospheric_Soil,MID24,64,570,Non,Non,Non,Non,Non,Non,Non,Non,CH3a11.1.Su.S.64

>

HU2.0.Su.S.65,TCGTCGCTCG,CACGACGTTGTAAAACGACGTGCCAGCMGCCGCGGTAA,CAGGAAACAGCTATGACCGGACTACVSGGGTATCTAAT,run2pool1,SRR3330056,NotUsed,Huges,Soybean,2,Vegetative,Rhizospheric_Soil,MID25,65,99,Non,Non,Non,Non,Non,Non,Non,Non,HU2.0.Su.S.65

>

HU2a11.1.Su.S.66,ACATACGCGT,CACGACGTTGTAAAACGACGTGCCAGCMGCCGCGGTAA,CAGGAAACAGCTATGACCGGACTACVSGGGTATCTAAT,run2pool1,SRR3330056,Used,Huges,Soybean,2,Vegetative,Rhizospheric_Soil,MID26,66,620,10_soybean_RA_RS,Non,Non,13_soybean_RS,Non,Non,16_wheat_soybean_RA_RS,Non,HU2a11.1.Su.S.66

>
HU3.0.Su.S.67,ACGCGAGTAT,CACGACGTTGTAAAACGACGTGCCAGCMGCCGCGGTAA,CAGGAAACAGCTATGACCGGACTACVSGGGTATCTAAT,run2pool1,SRR3330056,Used,Huges,Soybean,3,Vegetative,Rhizospheric_Soil,MID27,67,547,10_soybean_RA_RS,Non,Non,13_soybean_RS,Non,Non,16_wheat_soybean_RA_RS,Non,HU3.0.Su.S.67
>
HU3a11.1.Su.S.68,ACTACTATGT,CACGACGTTGTAAAACGACGTGCCAGCMGCCGCGGTAA,CAGGAAACAGCTATGACCGGACTACVSGGGTATCTAAT,run2pool1,SRR3330056,Used,Huges,Soybean,3,Vegetative,Rhizospheric_Soil,MID28,68,663,10_soybean_RA_RS,Non,Non,13_soybean_RS,Non,Non,16_wheat_soybean_RA_RS,Non,HU3a11.1.Su.S.68
>
MB2.0.Su.S.69,TCTACGTAGC,CACGACGTTGTAAAACGACGTGCCAGCMGCCGCGGTAA,CAGGAAACAGCTATGACCGGACTACVSGGGTATCTAAT,run2pool1,SRR3330056,Used,MonteBuey,Soybean,2,Vegetative,Rhizospheric_Soil,MID18,69,1140,10_soybean_RA_RS,Non,Non,13_soybean_RS,Non,Non,16_wheat_soybean_RA_RS,Non,MB2.0.Su.S.69
>
MB2a11.1.Su.S.70,ACTGTACAGT,CACGACGTTGTAAAACGACGTGCCAGCMGCCGCGGTAA,CAGGAAACAGCTATGACCGGACTACVSGGGTATCTAAT,run2pool1,SRR3330056,Used,MonteBuey,Soybean,2,Vegetative,Rhizospheric_Soil,MID29,70,652,10_soybean_RA_RS,Non,Non,13_soybean_RS,Non,Non,16_wheat_soybean_RA_RS,Non,MB2a11.1.Su.S.70
>
MB3.0.Su.S.71,AGACTATACT,CACGACGTTGTAAAACGACGTGCCAGCMGCCGCGGTAA,CAGGAAACAGCTATGACCGGACTACVSGGGTATCTAAT,run2pool1,SRR3330056,Used,MonteBuey,Soybean,3,Vegetative,Rhizospheric_Soil,MID30,71,459,10_soybean_RA_RS,Non,Non,13_soybean_RS,Non,Non,16_wheat_soybean_RA_RS,Non,MB3.0.Su.S.71
>
SA2.0.Su.S.73,AGTACGCTAT,CACGACGTTGTAAAACGACGTGCCAGCMGCCGCGGTAA,CAGGAAACAGCTATGACCGGACTACVSGGGTATCTAAT,run2pool1,SRR3330056,NotUsed,SanAgustin,Soybean,2,Vegetative,Rhizospheric_Soil,MID32,73,245,Non,Non,Non,Non,Non,Non,Non,Non,SA2.0.Su.S.73
>
SA2a11.1.Su.S.74,TGTACTACTC,CACGACGTTGTAAAACGACGTGCCAGCMGCCGCGGTAA,CAGGAAACAGCTATGACCGGACTACVSGGGTATCTAAT,run2pool1,SRR3330056,Used,SanAgustin,Soybean,2,Vegetative,Rhizospheric_Soil,MID19,74,470,10_soybean_RA_RS,Non,Non,13_soybean_RS,Non,Non,16_wheat_soybean_RA_RS,Non,SA2a11.1.Su.S.74
>
SA3.0.Su.S.75,ATAGAGTACT,CACGACGTTGTAAAACGACGTGCCAGCMGCCGCGGTAA,CAGGAAACAGCTATGACCGGACTACVSGGGTATCTAAT,run2pool1,SRR3330056,Used,SanAgustin,Soybean,3,Vegetative,Rhizospheric_Soil,MID33,75,884,10_soybean_RA_RS,Non,Non,13_soybean_RS,Non,Non,16_wheat_soybean_RA_RS,Non,SA3.0.Su.S.75
>
SA3a11.1.Su.S.76,CACGCTACGT,CACGACGTTGTAAAACGACGTGCCAGCMGCCGCGGTAA,CAGGAAACAGCTATGACCGGACTACVSGGGTATCTAAT,run2pool1,SRR3330056,Used,SanAgustin,Soybean,3,Vegetative,Rhizospheric_Soil,MID34,76,501,10_soybean_RA_RS,Non,Non,13_soybean_RS,Non,Non,16_wheat_soybean_RA_RS,Non,SA3a11.1.Su.S.76
>
VS2.0.Su.S.77,ACGACTACAG,CACGACGTTGTAAAACGACGTGCCAGCMGCCGCGGTAA,CAGGAAACAGCTATGACCGGACTACVSGGGTATCTAAT,run2pool1,SRR3330056,Used,VillaSaboya,Soybean,2,Vegetative,Rhizospheric_Soil,MID20,77,999,10_soybean_RA_RS,Non,Non,13_soybean_RS,Non,Non,16_wheat_soybean_RA_RS,Non,VS2.0.Su.S.77
>
VS2a11.1.Su.S.78,CAGTAGACGT,CACGACGTTGTAAAACGACGTGCCAGCMGCCGCGGTAA,CAGGAAACAGCTATGACCGGACTACVSGGGTATCTAAT,run2pool1,SRR3330056,Used,VillaSaboya,Soybean,2,Vegetative,Rhizospheric_Soil,MID35,78,599,10_soybean_RA_RS,Non,Non,13_soybean_RS,Non,Non,16_wheat_soybean_RA_RS,Non,VS2a11.1.Su.S.78
>
VS3.0.Su.S.79,CGTAGACTAG,CACGACGTTGTAAAACGACGTGCCAGCMGCCGCGGTAA,CAGGAAACAGCTATGACCGGACTACVSGGGTATCTAAT,run2pool1,SRR3330056,NotUsed,VillaSaboya,Soybean,3,Vegetative,Rhizospheric_Soil,MID21,79,370,Non,Non,Non,Non,Non,Non,Non,Non,VS3.0.Su.S.79
>
VS3a11.1.Su.S.80,CGACGTGACT,CACGACGTTGTAAAACGACGTGCCAGCMGCCGCGGTAA,CAGGAAACAGCTATGACCGGACTACVSGGGTATCTAAT,run2pool1,SRR3330056,Used,VillaSaboya,Soybean,3,Vegetative,Rhizospheric_Soil,MID36,80,495,10_soybean_RA_RS,Non,Non,13_soybean_RS,Non,Non,16_wheat_soybean_RA_RS,Non,VS3a11.1.Su.S.80
>
AR2.0.En.S.49,AGAGCGTCAC,CACGACGTTGTAAAACGACGTGCCAGCMGCCGCGGTAA,CAGGAAACAGCTATGACCGGACTACVSGGGTATCTAAT,run2pool1,SRR3330056,Used,Aranguren,Soybean,2,Vegetative,Root_Associated,MID81,49,1786,10_soybean_RA_RS,Non,12_soybean_RA,Non,Non,Non,16_wheat_soybean_RA_RS,17_wheat_soybean_RA,AR2.0.En.S.49
>

AR2a11.1.En.S.50,AGCGACTAGC,CACGACGTTGTAAAACGACGTGCCAGCMGCCGCGGTAA,CAGGAAACAGCTATGACCGGACTACVSGGGTATC
TAAT,run2pool1,SRR3330056,Used,Aranguren,Soybean,2,Vegetative,Root_Associated,MID82,50,1329,10_soybea
n_RA_RS,Non,12_soybean_RA,Non,Non,Non,16_wheat_soybean_RA_RS,17_wheat_soybean_RA,AR2a11.1.En.S.50
>
AR3.0.En.S.51,AGTAGTGATC,CACGACGTTGTAAAACGACGTGCCAGCMGCCGCGGTAA,CAGGAAACAGCTATGACCGGACTACVSGGGTATCTAA
T,run2pool1,SRR3330056,Used,Aranguren,Soybean,3,Vegetative,Root_Associated,MID83,51,1414,10_soybean_R
A_RS,Non,12_soybean_RA,Non,Non,Non,16_wheat_soybean_RA_RS,17_wheat_soybean_RA,AR3.0.En.S.51
>
AR3a11.1.En.S.52,CGAGACGCGC,CACGACGTTGTAAAACGACGTGCCAGCMGCCGCGGTAA,CAGGAAACAGCTATGACCGGACTACVSGGGTATC
TAAT,run2pool1,SRR3330056,Used,Aranguren,Soybean,3,Vegetative,Root_Associated,MID93,52,494,Non,Non,12
_soybean_RA,Non,Non,Non,Non,17_wheat_soybean_RA,AR3a11.1.En.S.52
>
CA2.0.En.S.53,AGTGACACAC,CACGACGTTGTAAAACGACGTGCCAGCMGCCGCGGTAA,CAGGAAACAGCTATGACCGGACTACVSGGGTATCTAA
T,run2pool1,SRR3330056,Used,CarmendeAreco,Soybean,2,Vegetative,Root_Associated,MID84,53,1169,10_soybe
an_RA_RS,Non,12_soybean_RA,Non,Non,Non,16_wheat_soybean_RA_RS,17_wheat_soybean_RA,CA2.0.En.S.53
>
CA2a11.1.En.S.54,AGTGTATGTC,CACGACGTTGTAAAACGACGTGCCAGCMGCCGCGGTAA,CAGGAAACAGCTATGACCGGACTACVSGGGTATC
TAAT,run2pool1,SRR3330056,Used,CarmendeAreco,Soybean,2,Vegetative,Root_Associated,MID85,54,1478,10_so
ybean_RA_RS,Non,12_soybean_RA,Non,Non,Non,16_wheat_soybean_RA_RS,17_wheat_soybean_RA,CA2a11.1.En.S.54
>
CA3.0.En.S.55,ATAGATAGAC,CACGACGTTGTAAAACGACGTGCCAGCMGCCGCGGTAA,CAGGAAACAGCTATGACCGGACTACVSGGGTATCTAA
T,run2pool1,SRR3330056,Used,CarmendeAreco,Soybean,3,Vegetative,Root_Associated,MID86,55,1524,10_soybe
an_RA_RS,Non,12_soybean_RA,Non,Non,Non,16_wheat_soybean_RA_RS,17_wheat_soybean_RA,CA3.0.En.S.55
>
CA3a11.1.En.S.56,ATATAGTCGC,CACGACGTTGTAAAACGACGTGCCAGCMGCCGCGGTAA,CAGGAAACAGCTATGACCGGACTACVSGGGTATC
TAAT,run2pool1,SRR3330056,Used,CarmendeAreco,Soybean,3,Vegetative,Root_Associated,MID87,56,1496,Non,N
on,12_soybean_RA,Non,Non,Non,Non,17_wheat_soybean_RA,CA3a11.1.En.S.56
>
CB2.0.En.S.57,ATCTACTGAC,CACGACGTTGTAAAACGACGTGCCAGCMGCCGCGGTAA,CAGGAAACAGCTATGACCGGACTACVSGGGTATCTAA
T,run2pool1,SRR3330056,Used,CorraldeBustos,Soybean,2,Vegetative,Root_Associated,MID88,57,1086,Non,Non
,12_soybean_RA,Non,Non,Non,Non,17_wheat_soybean_RA,CB2.0.En.S.57
>
CB2a5.1.En.S.58,CACGTAGATC,CACGACGTTGTAAAACGACGTGCCAGCMGCCGCGGTAA,CAGGAAACAGCTATGACCGGACTACVSGGGTATCT
AAT,run2pool1,SRR3330056,Used,CorraldeBustos,Soybean,2,Vegetative,Root_Associated,MID89,58,1475,Non,N
on,12_soybean_RA,Non,Non,Non,Non,17_wheat_soybean_RA,CB2a5.1.En.S.58
>
CB3.0.En.S.59,CACGTGTCGC,CACGACGTTGTAAAACGACGTGCCAGCMGCCGCGGTAA,CAGGAAACAGCTATGACCGGACTACVSGGGTATCTAA
T,run2pool1,SRR3330056,Used,CorraldeBustos,Soybean,3,Vegetative,Root_Associated,MID90,59,1275,Non,Non
,12_soybean_RA,Non,Non,Non,Non,17_wheat_soybean_RA,CB3.0.En.S.59
>
CB3a11.1.En.S.60,CATAGTAGTG,CACGACGTTGTAAAACGACGTGCCAGCMGCCGCGGTAA,CAGGAAACAGCTATGACCGGACTACVSGGGTATC
TAAT,run2pool1,SRR3330056,NotUsed,CorraldeBustos,Soybean,3,Vegetative,Root_Associated,MID13,60,303,No
n,Non,Non,Non,Non,Non,Non,Non,CB3a11.1.En.S.60
>
CH2.0.En.S.61,CATACTCTAC,CACGACGTTGTAAAACGACGTGCCAGCMGCCGCGGTAA,CAGGAAACAGCTATGACCGGACTACVSGGGTATCTAA
T,run2pool1,SRR3330056,Used,Chilibroste,Soybean,2,Vegetative,Root_Associated,MID91,61,1399,10_soybean
_RA_RS,Non,12_soybean_RA,Non,Non,Non,16_wheat_soybean_RA_RS,17_wheat_soybean_RA,CH2.0.En.S.61
>
MB3a11.1.En.S.72,TGATACGTCT,CACGACGTTGTAAAACGACGTGCCAGCMGCCGCGGTAA,CAGGAAACAGCTATGACCGGACTACVSGGGTATC
TAAT,run2pool1,SRR3330056,Used,MonteBuey,Soybean,3,Vegetative,Root_Associated,MID11,72,565,Non,Non,12
_soybean_RA,Non,Non,Non,Non,17_wheat_soybean_RA,MB3a11.1.En.S.72
>
SA3.0.En.S.75,CGAGAGATAC,CACGACGTTGTAAAACGACGTGCCAGCMGCCGCGGTAA,CAGGAAACAGCTATGACCGGACTACVSGGGTATCTAA
T,run2pool1,SRR3330056,Used,SanAgustin,Soybean,3,Vegetative,Root_Associated,MID14,75,785,Non,Non,12_s
oybean_RA,Non,Non,Non,Non,17_wheat_soybean_RA,SA3.0.En.S.75
>
BA3.1.En.TF.8,AGCTCACGTA,CACGACGTTGTAAAACGACGTGCCAGCMGCCGCGGTAA,CAGGAAACAGCTATGACCGGACTACVSGGGTATCTAA
T,run2pool1,SRR3330056,NotUsed,Balcarce,Wheat,3,Flowering,Root_Associated,MID51,8,696,Non,Non,Non,Non
,Non,Non,Non,Non,BA3.1.En.TF.8

>
BA4.0.En.T.9,AGTATACATA,CACGACGTTGTAAAACGACGTGCCAGCMGCCGCGGTAA,CAGGAAACAGCTATGACCGGACTACVSGGGTATCTAAT
,run2pool1,SRR3330056,NotUsed,Balcarce,Wheat,4,Tillering,Root_Associated,MID52,9,112,Non,Non,Non,Non,
Non,Non,Non,Non,BA4.0.En.T.9
>
BA4.0.En.TF.10,AGTCGAGAGA,CACGACGTTGTAAAACGACGTGCCAGCMGCCGCGGTAA,CAGGAAACAGCTATGACCGGACTACVSGGGTATCTA
AT,run2pool1,SRR3330056,NotUsed,Balcarce,Wheat,4,Flowering,Root_Associated,MID53,10,462,Non,Non,Non,N
on,Non,Non,Non,Non,BA4.0.En.TF.10
>
BA4.1.En.T.11,AGTGCTACGA,CACGACGTTGTAAAACGACGTGCCAGCMGCCGCGGTAA,CAGGAAACAGCTATGACCGGACTACVSGGGTATCTAA
T,run2pool1,SRR3330056,Used,Balcarce,Wheat,4,Tillering,Root_Associated,MID54,11,468,Non,11_wheat_RA_R
S,Non,Non,14_wheat_RA,Non,16_wheat_soybean_RA_RS,17_wheat_soybean_RA,BA4.1.En.T.11
>
BA4.1.En.TF.12,CGATCGTATA,CACGACGTTGTAAAACGACGTGCCAGCMGCCGCGGTAA,CAGGAAACAGCTATGACCGGACTACVSGGGTATCTA
AT,run2pool1,SRR3330056,NotUsed,Balcarce,Wheat,4,Flowering,Root_Associated,MID55,12,182,Non,Non,Non,N
on,Non,Non,Non,Non,BA4.1.En.TF.12
>
BA5.1.0.En.T.13,CGCAGTACGA,CACGACGTTGTAAAACGACGTGCCAGCMGCCGCGGTAA,CAGGAAACAGCTATGACCGGACTACVSGGGTATCT
AAT,run2pool1,SRR3330056,NotUsed,Balcarce,Wheat,5,Tillering,Root_Associated,MID56,13,95,Non,Non,Non,N
on,Non,Non,Non,Non,BA5.1.0.En.T.13
>
BA5.1.0.En.TF.14,CGCGTATACA,CACGACGTTGTAAAACGACGTGCCAGCMGCCGCGGTAA,CAGGAAACAGCTATGACCGGACTACVSGGGTATC
TAAT,run2pool1,SRR3330056,NotUsed,Balcarce,Wheat,5,Flowering,Root_Associated,MID57,14,269,Non,Non,Non
,Non,Non,Non,Non,Non,BA5.1.0.En.TF.14
>
BA5.1.1.En.T.15,CGTACAGTCA,CACGACGTTGTAAAACGACGTGCCAGCMGCCGCGGTAA,CAGGAAACAGCTATGACCGGACTACVSGGGTATCT
AAT,run2pool1,SRR3330056,Used,Balcarce,Wheat,5,Tillering,Root_Associated,MID58,15,833,Non,11_wheat_RA
_RS,Non,Non,14_wheat_RA,Non,16_wheat_soybean_RA_RS,17_wheat_soybean_RA,BA5.1.1.En.T.15
>
BA5.1.1.En.TF.16,CGTACTCAGA,CACGACGTTGTAAAACGACGTGCCAGCMGCCGCGGTAA,CAGGAAACAGCTATGACCGGACTACVSGGGTATC
TAAT,run2pool1,SRR3330056,NotUsed,Balcarce,Wheat,5,Flowering,Root_Associated,MID59,16,1412,Non,Non,No
n,Non,Non,Non,Non,Non,BA5.1.1.En.TF.16
>
BA5.2.1.En.T.19,CTACGCTCTA,CACGACGTTGTAAAACGACGTGCCAGCMGCCGCGGTAA,CAGGAAACAGCTATGACCGGACTACVSGGGTATCT
AAT,run2pool1,SRR3330056,NotUsed,Balcarce,Wheat,5,Tillering,Root_Associated,MID60,19,432,Non,Non,Non,
Non,Non,Non,Non,Non,BA5.2.1.En.T.19
>
BA5.2.1.En.TF.20,CTATAGCGTA,CACGACGTTGTAAAACGACGTGCCAGCMGCCGCGGTAA,CAGGAAACAGCTATGACCGGACTACVSGGGTATC
TAAT,run2pool1,SRR3330056,NotUsed,Balcarce,Wheat,5,Flowering,Root_Associated,MID61,20,984,Non,Non,Non
,Non,Non,Non,Non,Non,BA5.2.1.En.TF.20
>
BA5.3.0.En.T.21,TACGTCATCA,CACGACGTTGTAAAACGACGTGCCAGCMGCCGCGGTAA,CAGGAAACAGCTATGACCGGACTACVSGGGTATCT
AAT,run2pool1,SRR3330056,Used,Balcarce,Wheat,5,Tillering,Root_Associated,MID62,21,610,Non,11_wheat_RA
_RS,Non,Non,14_wheat_RA,Non,16_wheat_soybean_RA_RS,17_wheat_soybean_RA,BA5.3.0.En.T.21
>
BA5.3.0.En.TF.22,TAGTCGCATA,CACGACGTTGTAAAACGACGTGCCAGCMGCCGCGGTAA,CAGGAAACAGCTATGACCGGACTACVSGGGTATC
TAAT,run2pool1,SRR3330056,NotUsed,Balcarce,Wheat,5,Flowering,Root_Associated,MID63,22,1023,Non,Non,No
n,Non,Non,Non,Non,Non,BA5.3.0.En.TF.22
>
BA5.3.1.En.T.23,TATATATACA,CACGACGTTGTAAAACGACGTGCCAGCMGCCGCGGTAA,CAGGAAACAGCTATGACCGGACTACVSGGGTATCT
AAT,run2pool1,SRR3330056,NotUsed,Balcarce,Wheat,5,Tillering,Root_Associated,MID64,23,261,Non,Non,Non,
Non,Non,Non,Non,Non,BA5.3.1.En.T.23
>
CB2.0.En.T.25,TATGCTAGTA,CACGACGTTGTAAAACGACGTGCCAGCMGCCGCGGTAA,CAGGAAACAGCTATGACCGGACTACVSGGGTATCTAA
T,run2pool1,SRR3330056,NotUsed,CorraldeBustos,Wheat,2,Tillering,Root_Associated,MID65,25,474,Non,Non,
Non,Non,Non,Non,Non,Non,CB2.0.En.T.25
>

CB2.1.En.T.26,TCACGCGAGA,CACGACGTTGTAAAACGACGTGCCAGCMGCCGCGGTAA,CAGGAAACAGCTATGACCGGACTACVSGGGTATCTAA
T,run2pool1,SRR3330056,NotUsed,CorraldeBustos,Wheat,2,Tillering,Root_Associated,MID66,26,430,Non,Non,
Non,Non,Non,Non,Non,Non,CB2.1.En.T.26
>
CB3.0.En.T.27,TCGATAGTGA,CACGACGTTGTAAAACGACGTGCCAGCMGCCGCGGTAA,CAGGAAACAGCTATGACCGGACTACVSGGGTATCTAA
T,run2pool1,SRR3330056,Used,CorraldeBustos,Wheat,3,Tillering,Root_Associated,MID67,27,671,Non,11_whea
t_RA_RS,Non,Non,14_wheat_RA,Non,16_wheat_soybean_RA_RS,17_wheat_soybean_RA,CB3.0.En.T.27
>
BA2.0.En.T.1,TCTATACTAT,CACGACGTTGTAAAACGACGTGCCAGCMGCCGCGGTAA,CAGGAAACAGCTATGACCGGACTACVSGGGTATCTAAT
,run2pool2,SRR3330057,NotUsed,Balcarce,Wheat,2,Tillering,Root_Associated,MID45,1,308,Non,Non,Non,Non,
Non,Non,Non,Non,BA2.0.En.T.1
>
BA2.0.En.TF.2,TGACGTATGT,CACGACGTTGTAAAACGACGTGCCAGCMGCCGCGGTAA,CAGGAAACAGCTATGACCGGACTACVSGGGTATCTAA
T,run2pool2,SRR3330057,NotUsed,Balcarce,Wheat,2,Flowering,Root_Associated,MID46,2,495,Non,Non,Non,Non
,Non,Non,Non,Non,BA2.0.En.TF.2
>
BA2.1.En.T.3,TGTGAGTAGT,CACGACGTTGTAAAACGACGTGCCAGCMGCCGCGGTAA,CAGGAAACAGCTATGACCGGACTACVSGGGTATCTAAT
,run2pool2,SRR3330057,Used,Balcarce,Wheat,2,Tillering,Root_Associated,MID47,3,916,Non,11_wheat_RA_RS,
Non,Non,14_wheat_RA,Non,16_wheat_soybean_RA_RS,17_wheat_soybean_RA,BA2.1.En.T.3
>
BA2.1.En.TF.4,ACAGTATATA,CACGACGTTGTAAAACGACGTGCCAGCMGCCGCGGTAA,CAGGAAACAGCTATGACCGGACTACVSGGGTATCTAA
T,run2pool2,SRR3330057,NotUsed,Balcarce,Wheat,2,Flowering,Root_Associated,MID48,4,1679,Non,Non,Non,No
n,Non,Non,Non,Non,BA2.1.En.TF.4
>
BA3.0.En.T.5,ACGCGATCGA,CACGACGTTGTAAAACGACGTGCCAGCMGCCGCGGTAA,CAGGAAACAGCTATGACCGGACTACVSGGGTATCTAAT
,run2pool2,SRR3330057,NotUsed,Balcarce,Wheat,3,Tillering,Root_Associated,MID49,5,1550,Non,Non,Non,Non
,Non,Non,Non,Non,BA3.0.En.T.5
>
BA3.0.En.TF.6,ACTAGCAGTA,CACGACGTTGTAAAACGACGTGCCAGCMGCCGCGGTAA,CAGGAAACAGCTATGACCGGACTACVSGGGTATCTAA
T,run2pool2,SRR3330057,NotUsed,Balcarce,Wheat,3,Flowering,Root_Associated,MID50,6,1740,Non,Non,Non,No
n,Non,Non,Non,Non,BA3.0.En.TF.6
>
BA3.1.En.T.7,ACGACTACAG,CACGACGTTGTAAAACGACGTGCCAGCMGCCGCGGTAA,CAGGAAACAGCTATGACCGGACTACVSGGGTATCTAAT
,run2pool2,SRR3330057,Used,Balcarce,Wheat,3,Tillering,Root_Associated,MID20,7,4384,Non,11_wheat_RA_RS
,Non,Non,14_wheat_RA,Non,16_wheat_soybean_RA_RS,17_wheat_soybean_RA,BA3.1.En.T.7
>
BA5.2.0.En.T.17,CGTAGACTAG,CACGACGTTGTAAAACGACGTGCCAGCMGCCGCGGTAA,CAGGAAACAGCTATGACCGGACTACVSGGGTATCT
AAT,run2pool2,SRR3330057,Used,Balcarce,Wheat,5,Tillering,Root_Associated,MID21,17,1343,Non,11_wheat_R
A_RS,Non,Non,14_wheat_RA,Non,16_wheat_soybean_RA_RS,17_wheat_soybean_RA,BA5.2.0.En.T.17
>
BA5.2.0.En.TF.18,AGACTATACT,CACGACGTTGTAAAACGACGTGCCAGCMGCCGCGGTAA,CAGGAAACAGCTATGACCGGACTACVSGGGTATC
TAAT,run2pool2,SRR3330057,NotUsed,Balcarce,Wheat,5,Flowering,Root_Associated,MID30,18,1344,Non,Non,No
n,Non,Non,Non,Non,Non,BA5.2.0.En.TF.18
>
BA5.3.1.En.TF.24,AGCGTCGTCT,CACGACGTTGTAAAACGACGTGCCAGCMGCCGCGGTAA,CAGGAAACAGCTATGACCGGACTACVSGGGTATC
TAAT,run2pool2,SRR3330057,NotUsed,Balcarce,Wheat,5,Flowering,Root_Associated,MID31,24,924,Non,Non,Non
,Non,Non,Non,Non,Non,BA5.3.1.En.TF.24
>
CB3.1.En.T.28,TCGCTGCGTA,CACGACGTTGTAAAACGACGTGCCAGCMGCCGCGGTAA,CAGGAAACAGCTATGACCGGACTACVSGGGTATCTAA
T,run2pool2,SRR3330057,Used,CorraldeBustos,Wheat,3,Tillering,Root_Associated,MID68,28,1987,Non,11_whe
at_RA_RS,Non,Non,14_wheat_RA,Non,16_wheat_soybean_RA_RS,17_wheat_soybean_RA,CB3.1.En.T.28
>
CB4.0.En.T.29,TCTGACGTCA,CACGACGTTGTAAAACGACGTGCCAGCMGCCGCGGTAA,CAGGAAACAGCTATGACCGGACTACVSGGGTATCTAA
T,run2pool2,SRR3330057,Used,CorraldeBustos,Wheat,4,Tillering,Root_Associated,MID69,29,1995,Non,11_whe
at_RA_RS,Non,Non,14_wheat_RA,Non,16_wheat_soybean_RA_RS,17_wheat_soybean_RA,CB4.0.En.T.29
>
CB4.1.En.T.30,TGAGTCAGTA,CACGACGTTGTAAAACGACGTGCCAGCMGCCGCGGTAA,CAGGAAACAGCTATGACCGGACTACVSGGGTATCTAA
T,run2pool2,SRR3330057,Used,CorraldeBustos,Wheat,4,Tillering,Root_Associated,MID70,30,2838,Non,11_whe
at_RA_RS,Non,Non,14_wheat_RA,Non,16_wheat_soybean_RA_RS,17_wheat_soybean_RA,CB4.1.En.T.30

>
CB5.0.En.T.31,TGTAGTGTGA,CACGACGTTGTAAAACGACGTGCCAGCMGCCGCGGTAA,CAGGAAACAGCTATGACCGGACTACVSGGGTATCTAA
T,run2pool2,SRR3330057,Used,CorraldeBustos,Wheat,5,Tillering,Root_Associated,MID71,31,832,Non,11_whea
t_RA_RS,Non,Non,14_wheat_RA,Non,16_wheat_soybean_RA_RS,17_wheat_soybean_RA,CB5.0.En.T.31
>
CB5.1.En.T.32,TGTCACACGA,CACGACGTTGTAAAACGACGTGCCAGCMGCCGCGGTAA,CAGGAAACAGCTATGACCGGACTACVSGGGTATCTAA
T,run2pool2,SRR3330057,Used,CorraldeBustos,Wheat,5,Tillering,Root_Associated,MID72,32,1240,Non,11_whe
at_RA_RS,Non,Non,14_wheat_RA,Non,16_wheat_soybean_RA_RS,17_wheat_soybean_RA,CB5.1.En.T.32
>
DA2.0.En.T.33,TGTCGTCGCA,CACGACGTTGTAAAACGACGTGCCAGCMGCCGCGGTAA,CAGGAAACAGCTATGACCGGACTACVSGGGTATCTAA
T,run2pool2,SRR3330057,Used,Daireaux,Wheat,2,Tillering,Root_Associated,MID73,33,1622,Non,11_wheat_RA_
RS,Non,Non,14_wheat_RA,Non,16_wheat_soybean_RA_RS,17_wheat_soybean_RA,DA2.0.En.T.33
>
DA2.1.En.T.34,ACACATACGC,CACGACGTTGTAAAACGACGTGCCAGCMGCCGCGGTAA,CAGGAAACAGCTATGACCGGACTACVSGGGTATCTAA
T,run2pool2,SRR3330057,NotUsed,Daireaux,Wheat,2,Tillering,Root_Associated,MID74,34,1639,Non,Non,Non,N
on,Non,Non,Non,Non,DA2.1.En.T.34
>
DA3.0.En.T.35,ACAGTCGTGC,CACGACGTTGTAAAACGACGTGCCAGCMGCCGCGGTAA,CAGGAAACAGCTATGACCGGACTACVSGGGTATCTAA
T,run2pool2,SRR3330057,NotUsed,Daireaux,Wheat,3,Tillering,Root_Associated,MID75,35,1810,Non,Non,Non,N
on,Non,Non,Non,Non,DA3.0.En.T.35
>
DA3.1.En.T.36,ACATGACGAC,CACGACGTTGTAAAACGACGTGCCAGCMGCCGCGGTAA,CAGGAAACAGCTATGACCGGACTACVSGGGTATCTAA
T,run2pool2,SRR3330057,Used,Daireaux,Wheat,3,Tillering,Root_Associated,MID76,36,1665,Non,11_wheat_RA_
RS,Non,Non,14_wheat_RA,Non,16_wheat_soybean_RA_RS,17_wheat_soybean_RA,DA3.1.En.T.36
>
DA4.0.En.T.37,ACGACAGCTC,CACGACGTTGTAAAACGACGTGCCAGCMGCCGCGGTAA,CAGGAAACAGCTATGACCGGACTACVSGGGTATCTAA
T,run2pool2,SRR3330057,Used,Daireaux,Wheat,4,Tillering,Root_Associated,MID77,37,2211,Non,11_wheat_RA_
RS,Non,Non,14_wheat_RA,Non,16_wheat_soybean_RA_RS,17_wheat_soybean_RA,DA4.0.En.T.37
>
DA4.1.En.T.38,TACGAGTATG,CACGACGTTGTAAAACGACGTGCCAGCMGCCGCGGTAA,CAGGAAACAGCTATGACCGGACTACVSGGGTATCTAA
T,run2pool2,SRR3330057,Used,Daireaux,Wheat,4,Tillering,Root_Associated,MID22,38,2814,Non,11_wheat_RA_
RS,Non,Non,14_wheat_RA,Non,16_wheat_soybean_RA_RS,17_wheat_soybean_RA,DA4.1.En.T.38
>
DA5.0.En.T.39,ACGTCTCATC,CACGACGTTGTAAAACGACGTGCCAGCMGCCGCGGTAA,CAGGAAACAGCTATGACCGGACTACVSGGGTATCTAA
T,run2pool2,SRR3330057,Used,Daireaux,Wheat,5,Tillering,Root_Associated,MID78,39,2252,Non,11_wheat_RA_
RS,Non,Non,14_wheat_RA,Non,16_wheat_soybean_RA_RS,17_wheat_soybean_RA,DA5.0.En.T.39
>
DA5.1.En.T.40,ACTCATCTAC,CACGACGTTGTAAAACGACGTGCCAGCMGCCGCGGTAA,CAGGAAACAGCTATGACCGGACTACVSGGGTATCTAA
T,run2pool2,SRR3330057,Used,Daireaux,Wheat,5,Tillering,Root_Associated,MID79,40,1393,Non,11_wheat_RA_
RS,Non,Non,14_wheat_RA,Non,16_wheat_soybean_RA_RS,17_wheat_soybean_RA,DA5.1.En.T.40
>
VS2.0.En.T.41,TACTCTCGTG,CACGACGTTGTAAAACGACGTGCCAGCMGCCGCGGTAA,CAGGAAACAGCTATGACCGGACTACVSGGGTATCTAA
T,run2pool2,SRR3330057,Used,VillaSaboya,Wheat,2,Tillering,Root_Associated,MID23,41,1379,Non,11_wheat_
RA_RS,Non,Non,14_wheat_RA,Non,16_wheat_soybean_RA_RS,17_wheat_soybean_RA,VS2.0.En.T.41
>
VS2.1.En.T.42,ACGCGAGTAT,CACGACGTTGTAAAACGACGTGCCAGCMGCCGCGGTAA,CAGGAAACAGCTATGACCGGACTACVSGGGTATCTAA
T,run2pool2,SRR3330057,Used,VillaSaboya,Wheat,2,Tillering,Root_Associated,MID27,42,825,Non,11_wheat_R
A_RS,Non,Non,14_wheat_RA,Non,16_wheat_soybean_RA_RS,17_wheat_soybean_RA,VS2.1.En.T.42
>
VS3.0.En.T.43,ACTACTATGT,CACGACGTTGTAAAACGACGTGCCAGCMGCCGCGGTAA,CAGGAAACAGCTATGACCGGACTACVSGGGTATCTAA
T,run2pool2,SRR3330057,Used,VillaSaboya,Wheat,3,Tillering,Root_Associated,MID28,43,356,Non,Non,Non,No
n,Non,Non,Non,17_wheat_soybean_RA,VS3.0.En.T.43
>
VS3.1.En.T.44,CGAGAGATAC,CACGACGTTGTAAAACGACGTGCCAGCMGCCGCGGTAA,CAGGAAACAGCTATGACCGGACTACVSGGGTATCTAA
T,run2pool2,SRR3330057,Used,VillaSaboya,Wheat,3,Tillering,Root_Associated,MID14,44,663,Non,11_wheat_R
A_RS,Non,Non,14_wheat_RA,Non,16_wheat_soybean_RA_RS,17_wheat_soybean_RA,VS3.1.En.T.44
>

VS4.0.En.T.45,TCGTCGCTCG,CACGACGTTGTAAAACGACGTGCCAGCMGCCGCGGTAA,CAGGAAACAGCTATGACCGGACTACVSGGGTATCTAA
T,run2pool2,SRR3330057,Used,VillaSaboya,Wheat,4,Tillering,Root_Associated,MID25,45,1219,Non,11_wheat_
RA_RS,Non,Non,14_wheat_RA,Non,16_wheat_soybean_RA_RS,17_wheat_soybean_RA,VS4.0.En.T.45
>
VS4.1.En.T.46,ACTGTACAGT,CACGACGTTGTAAAACGACGTGCCAGCMGCCGCGGTAA,CAGGAAACAGCTATGACCGGACTACVSGGGTATCTAA
T,run2pool2,SRR3330057,NotUsed,VillaSaboya,Wheat,4,Tillering,Root_Associated,MID29,46,485,Non,Non,Non
,Non,Non,Non,Non,Non,VS4.1.En.T.46
>
VS5.0.En.T.47,ACTCGCGCAC,CACGACGTTGTAAAACGACGTGCCAGCMGCCGCGGTAA,CAGGAAACAGCTATGACCGGACTACVSGGGTATCTAA
T,run2pool2,SRR3330057,Used,VillaSaboya,Wheat,5,Tillering,Root_Associated,MID80,47,2699,Non,11_wheat_
RA_RS,Non,Non,14_wheat_RA,Non,16_wheat_soybean_RA_RS,17_wheat_soybean_RA,VS5.0.En.T.47
>
VS5.1.En.T.48,TAGAGACGAG,CACGACGTTGTAAAACGACGTGCCAGCMGCCGCGGTAA,CAGGAAACAGCTATGACCGGACTACVSGGGTATCTAA
T,run2pool2,SRR3330057,Used,VillaSaboya,Wheat,5,Tillering,Root_Associated,MID24,48,2011,Non,11_wheat_
RA_RS,Non,Non,14_wheat_RA,Non,16_wheat_soybean_RA_RS,17_wheat_soybean_RA,VS5.1.En.T.48
>
BA2.1.Su.T.B.1,TACAGATCGT,CACGACGTTGTAAAACGACGTGCCAGCMGCCGCGGTAA,CAGGAAACAGCTATGACCGGACTACVSGGGTATCTA
AT,run2pool2,SRR3330057,Used,Balcarce,Wheat,2,Tillering,Rhizospheric_Soil,MID39,1,1144,Non,11_wheat_R
A_RS,Non,Non,Non,Non,16_wheat_soybean_RA_RS,Non,BA2.1.Su.T.B.1
>
MB4.0.Su.T.B.27,TCGATCACGT,CACGACGTTGTAAAACGACGTGCCAGCMGCCGCGGTAA,CAGGAAACAGCTATGACCGGACTACVSGGGTATCT
AAT,run2pool2,SRR3330057,Used,MonteBuey,Wheat,4,Tillering,Rhizospheric_Soil,MID42,27,2094,Non,Non,Non
,Non,Non,Non,Non,Non,MB4.0.Su.T.B.27
>
CB2.0.Su.T.B.32,TACGCTGTCT,CACGACGTTGTAAAACGACGTGCCAGCMGCCGCGGTAA,CAGGAAACAGCTATGACCGGACTACVSGGGTATCT
AAT,run2pool2,SRR3330057,NotUsed,CorraldeBustos,Wheat,2,Tillering,Rhizospheric_Soil,MID40,32,771,Non,
Non,Non,Non,Non,Non,Non,Non,CB2.0.Su.T.B.32
>
DA2.0.Su.T.B.34,ACATACGCGT,CACGACGTTGTAAAACGACGTGCCAGCMGCCGCGGTAA,CAGGAAACAGCTATGACCGGACTACVSGGGTATCT
AAT,run2pool2,SRR3330057,Used,Daireaux,Wheat,2,Tillering,Rhizospheric_Soil,MID26,34,3574,Non,11_wheat
_RA_RS,Non,Non,Non,Non,16_wheat_soybean_RA_RS,Non,DA2.0.Su.T.B.34
>
VS2.0.Su.T.B.37,TCGCACTAGT,CACGACGTTGTAAAACGACGTGCCAGCMGCCGCGGTAA,CAGGAAACAGCTATGACCGGACTACVSGGGTATCT
AAT,run2pool2,SRR3330057,Used,VillaSaboya,Wheat,2,Tillering,Rhizospheric_Soil,MID43,37,2263,Non,11_wh
eat_RA_RS,Non,Non,Non,Non,16_wheat_soybean_RA_RS,Non,VS2.0.Su.T.B.37
>
MB2.0.Su.T.B.38,TAGTGTAGAT,CACGACGTTGTAAAACGACGTGCCAGCMGCCGCGGTAA,CAGGAAACAGCTATGACCGGACTACVSGGGTATCT
AAT,run2pool2,SRR3330057,Used,MonteBuey,Wheat,2,Tillering,Rhizospheric_Soil,MID41,38,2104,Non,Non,Non
,Non,Non,Non,Non,Non,MB2.0.Su.T.B.38
>
VS4.0.Su.T.B.43,TCTAGCGACT,CACGACGTTGTAAAACGACGTGCCAGCMGCCGCGGTAA,CAGGAAACAGCTATGACCGGACTACVSGGGTATCT
AAT,run2pool2,SRR3330057,Used,VillaSaboya,Wheat,4,Tillering,Rhizospheric_Soil,MID44,43,1774,Non,11_wh
eat_RA_RS,Non,Non,Non,15_wheat_RS,16_wheat_soybean_RA_RS,Non,VS4.0.Su.T.B.43
>
SJ2.0.Su.T.B.54,TACACGTGAT,CACGACGTTGTAAAACGACGTGCCAGCMGCCGCGGTAA,CAGGAAACAGCTATGACCGGACTACVSGGGTATCT
AAT,run2pool2,SRR3330057,NotUsed,SanJorge,Wheat,2,Tillering,Rhizospheric_Soil,MID38,54,1874,Non,Non,N
on,Non,Non,Non,Non,Non,SJ2.0.Su.T.B.54
>
SJ5.2.0.Su.T.B.81,TACACACACT,CACGACGTTGTAAAACGACGTGCCAGCMGCCGCGGTAA,CAGGAAACAGCTATGACCGGACTACVSGGGTAT
CTAAT,run2pool2,SRR3330057,NotUsed,SanJorge,Wheat,5,Tillering,Rhizospheric_Soil,MID37,81,864,Non,Non,
Non,Non,Non,Non,Non,Non,SJ5.2.0.Su.T.B.81
>
SJ5.1.1.Su.T.B.82,TGATACGTCT,CACGACGTTGTAAAACGACGTGCCAGCMGCCGCGGTAA,CAGGAAACAGCTATGACCGGACTACVSGGGTAT
CTAAT,run2pool2,SRR3330057,NotUsed,SanJorge,Wheat,5,Tillering,Rhizospheric_Soil,MID11,82,435,Non,Non,
Non,Non,Non,Non,Non,Non,SJ5.1.1.Su.T.B.82
>
CH2all.1.En.S.62,ACGAGTGCGT,CACGACGTTGTAAAACGACGTGCCAGCMGCCGCGGTAA,CAGGAAACAGCTATGACCGGACTACVSGGGTATC
TAAT,run2pool2,SRR3330057,Used,Chilibroste,Soybean,2,Vegetative,Root_Associated,MID1,62,2675,10_soybe
an_RA_RS,Non,12_soybean_RA,Non,Non,Non,16_wheat_soybean_RA_RS,17_wheat_soybean_RA,CH2all.1.En.S.62

>
CH3.0.En.S.63,ACGCTCGACA,CACGACGTTGTAAAACGACGTGCCAGCMGCCGCGGTAA,CAGGAAACAGCTATGACCGGACTACVSGGGTATCTAA
T,run2pool2,SRR3330057,Used,Chilibroste,Soybean,3,Vegetative,Root_Associated,MID2,63,5850,10_soybean_
RA_RS,Non,12_soybean_RA,Non,Non,Non,16_wheat_soybean_RA_RS,17_wheat_soybean_RA,CH3.0.En.S.63
>
CH3a11.1.En.S.64,AGACGCACTC,CACGACGTTGTAAAACGACGTGCCAGCMGCCGCGGTAA,CAGGAAACAGCTATGACCGGACTACVSGGGTATC
TAAT,run2pool2,SRR3330057,Used,Chilibroste,Soybean,3,Vegetative,Root_Associated,MID3,64,3177,Non,Non,
12_soybean_RA,Non,Non,Non,Non,17_wheat_soybean_RA,CH3a11.1.En.S.64
>
HU2.0.En.S.65,AGCACTGTAG,CACGACGTTGTAAAACGACGTGCCAGCMGCCGCGGTAA,CAGGAAACAGCTATGACCGGACTACVSGGGTATCTAA
T,run2pool2,SRR3330057,Used,Huges,Soybean,2,Vegetative,Root_Associated,MID4,65,2837,10_soybean_RA_RS,
Non,12_soybean_RA,Non,Non,Non,16_wheat_soybean_RA_RS,17_wheat_soybean_RA,HU2.0.En.S.65
>
HU2a11.1.En.S.66,ATCAGACACG,CACGACGTTGTAAAACGACGTGCCAGCMGCCGCGGTAA,CAGGAAACAGCTATGACCGGACTACVSGGGTATC
TAAT,run2pool2,SRR3330057,Used,Huges,Soybean,2,Vegetative,Root_Associated,MID5,66,3577,10_soybean_RA_
RS,Non,12_soybean_RA,Non,Non,Non,16_wheat_soybean_RA_RS,17_wheat_soybean_RA,HU2a11.1.En.S.66
>
HU3.0.En.S.67,ATATCGCGAG,CACGACGTTGTAAAACGACGTGCCAGCMGCCGCGGTAA,CAGGAAACAGCTATGACCGGACTACVSGGGTATCTAA
T,run2pool2,SRR3330057,Used,Huges,Soybean,3,Vegetative,Root_Associated,MID6,67,4159,10_soybean_RA_RS,
Non,12_soybean_RA,Non,Non,Non,16_wheat_soybean_RA_RS,17_wheat_soybean_RA,HU3.0.En.S.67
>
HU3a11.1.En.S.68,CGTGTCTCTA,CACGACGTTGTAAAACGACGTGCCAGCMGCCGCGGTAA,CAGGAAACAGCTATGACCGGACTACVSGGGTATC
TAAT,run2pool2,SRR3330057,Used,Huges,Soybean,3,Vegetative,Root_Associated,MID7,68,2795,Non,Non,12_soy
bean_RA,Non,Non,Non,Non,17_wheat_soybean_RA,HU3a11.1.En.S.68
>
MB2.0.En.S.69,CTCGCGTGTC,CACGACGTTGTAAAACGACGTGCCAGCMGCCGCGGTAA,CAGGAAACAGCTATGACCGGACTACVSGGGTATCTAA
T,run2pool2,SRR3330057,Used,MonteBuey,Soybean,2,Vegetative,Root_Associated,MID8,69,2174,10_soybean_RA
_RS,Non,12_soybean_RA,Non,Non,Non,16_wheat_soybean_RA_RS,17_wheat_soybean_RA,MB2.0.En.S.69
>
MB3.0.En.S.71,TCTCTATGCG,CACGACGTTGTAAAACGACGTGCCAGCMGCCGCGGTAA,CAGGAAACAGCTATGACCGGACTACVSGGGTATCTAA
T,run2pool2,SRR3330057,Used,MonteBuey,Soybean,3,Vegetative,Root_Associated,MID10,71,2858,10_soybean_R
A_RS,Non,12_soybean_RA,Non,Non,Non,16_wheat_soybean_RA_RS,17_wheat_soybean_RA,MB3.0.En.S.71
>
SA3a11.1.En.S.76,ATACGACGTA,CACGACGTTGTAAAACGACGTGCCAGCMGCCGCGGTAA,CAGGAAACAGCTATGACCGGACTACVSGGGTATC
TAAT,run2pool2,SRR3330057,Used,SanAgustin,Soybean,3,Vegetative,Root_Associated,MID15,76,2703,10_soybe
an_RA_RS,Non,12_soybean_RA,Non,Non,Non,16_wheat_soybean_RA_RS,17_wheat_soybean_RA,SA3a11.1.En.S.76
>
VS2.0.En.S.77,TCACGTACTA,CACGACGTTGTAAAACGACGTGCCAGCMGCCGCGGTAA,CAGGAAACAGCTATGACCGGACTACVSGGGTATCTAA
T,run2pool2,SRR3330057,Used,VillaSaboya,Soybean,2,Vegetative,Root_Associated,MID16,77,2475,Non,Non,12
_soybean_RA,Non,Non,Non,Non,17_wheat_soybean_RA,VS2.0.En.S.77
>
VS2a11.1.En.S.78,CGTCTAGTAC,CACGACGTTGTAAAACGACGTGCCAGCMGCCGCGGTAA,CAGGAAACAGCTATGACCGGACTACVSGGGTATC
TAAT,run2pool2,SRR3330057,Used,VillaSaboya,Soybean,2,Vegetative,Root_Associated,MID17,78,2908,10_soyb
ean_RA_RS,Non,12_soybean_RA,Non,Non,Non,16_wheat_soybean_RA_RS,17_wheat_soybean_RA,VS2a11.1.En.S.78
>
VS3.0.En.S.79,TCTACGTAGC,CACGACGTTGTAAAACGACGTGCCAGCMGCCGCGGTAA,CAGGAAACAGCTATGACCGGACTACVSGGGTATCTAA
T,run2pool2,SRR3330057,Used,VillaSaboya,Soybean,3,Vegetative,Root_Associated,MID18,79,3269,10_soybean
_RA_RS,Non,12_soybean_RA,Non,Non,Non,16_wheat_soybean_RA_RS,17_wheat_soybean_RA,VS3.0.En.S.79
>
VS3a11.1.En.S.80,TGTACTACTC,CACGACGTTGTAAAACGACGTGCCAGCMGCCGCGGTAA,CAGGAAACAGCTATGACCGGACTACVSGGGTATC
TAAT,run2pool2,SRR3330057,Used,VillaSaboya,Soybean,3,Vegetative,Root_Associated,MID19,80,2541,10_soyb
ean_RA_RS,Non,12_soybean_RA,Non,Non,Non,16_wheat_soybean_RA_RS,17_wheat_soybean_RA,VS3a11.1.En.S.80
>
SA2a11.1.En.S.74,CATAGTAGTG,CACGACGTTGTAAAACGACGTGCCAGCMGCCGCGGTAA,CAGGAAACAGCTATGACCGGACTACVSGGGTATC
TAAT,run2pool2,SRR3330057,Used,SanAgustin,Soybean,2,Vegetative,Root_Associated,MID13,74,1000,10_soybe
an_RA_RS,Non,12_soybean_RA,Non,Non,Non,16_wheat_soybean_RA_RS,17_wheat_soybean_RA,SA2a11.1.En.S.74
>

MB2a11.1.En.S.70,CGACACTATC,CACGACGTTGTAAAACGACGTGCCAGCMGCCGCGGTAA,CAGGAAACAGCTATGACCGGACTACVSGGGTATCTAAT,run2pool2,SRR3330057,Used,MonteBuey,Soybean,2,Vegetative,Root_Associated,MID92,70,3046,10_soybean_RA_RS,Non,12_soybean_RA,Non,Non,Non,16_wheat_soybean_RA_RS,17_wheat_soybean_RA,MB2a11.1.En.S.70

>

SA2.0.En.S.73,CGAGACGCGC,CACGACGTTGTAAAACGACGTGCCAGCMGCCGCGGTAA,CAGGAAACAGCTATGACCGGACTACVSGGGTATCTAAT,run2pool2,SRR3330057,Used,SanAgustin,Soybean,2,Vegetative,Root_Associated,MID93,73,2054,10_soybean_RA_RS,Non,12_soybean_RA,Non,Non,Non,16_wheat_soybean_RA_RS,17_wheat_soybean_RA,SA2.0.En.S.73
